# An integrative analysis of TFBS-clustered regions reveals new transcriptional regulation models on the accessible chromatin landscape

**DOI:** 10.1038/srep08465

**Published:** 2015-02-16

**Authors:** Hebing Chen, Hao Li, Feng Liu, Xiaofei Zheng, Shengqi Wang, Xiaochen Bo, Wenjie Shu

**Affiliations:** 1 Department of Biotechnology, Beijing Institute of Radiation Medicine, Beijing 100850, China; 2 Department of Biochemistry and Molecular Biology, Beijing Institute of Radiation Medicine, Beijing 100850, China

## Abstract

DNase I hypersensitive sites (DHSs) define the accessible chromatin landscape and have revolutionised the discovery of distinct *cis*-regulatory elements in diverse organisms. Here, we report the first comprehensive map of human transcription factor binding site (TFBS)-clustered regions using Gaussian kernel density estimation based on genome-wide mapping of the TFBSs in 133 human cell and tissue types. Approximately 1.6 million distinct TFBS-clustered regions, collectively spanning 27.7% of the human genome, were discovered. The TFBS complexity assigned to each TFBS-clustered region was highly correlated with genomic location, cell selectivity, evolutionary conservation, sequence features, and functional roles. An integrative analysis of these regions using ENCODE data revealed transcription factor occupancy, transcriptional activity, histone modification, DNA methylation, and chromatin structures that varied based on TFBS complexity. Furthermore, we found that we could recreate lineage-branching relationships by simple clustering of the TFBS-clustered regions from terminally differentiated cells. Based on these findings, a model of transcriptional regulation determined by TFBS complexity is proposed.

Sequence-specific transcription factors (TFs) interact with *cis*-regulatory elements encoded within regulatory DNA to displace nucleosomes, remodel chromatin, and create nuclease hypersensitivity^1^,^2^. Discovered over 30 years ago, DNase I hypersensitive sites (DHSs) have been used extensively to mark regulatory DNA and map active *cis*-regulatory elements in diverse organisms^2^,^3^,^4^. Advanced next-generation sequencing (NGS) technologies have enabled the genome-wide mapping of DHSs in mammalian cells^5^,^6^,^7^, revealing comprehensive catalogues of regulatory DNA.

In eukaryotes, multiple TFs cooperatively bind regulatory DNA to temporally and spatially control gene expression. Therefore, a full understanding how TFs contribute to the control of cellular transcriptional regulation requires an in-depth analysis of the complete ensemble of TF binding events in a cell. However, to date, high-throughput ChIP-seq (HT-ChIP-seq)^8^ and the ENCODE project^9^ have only enabled the investigation of roughly 200 TFs in 72 cell lines. Similarly, Yan *et al.* used HT-ChIP-seq to analyze 239 TFs in two colon cancer cell lines^10^. Despite the progress that has been made, these numbers are far lower than the estimated number of TFs that are encoded in the human genome or that are functional in a single cell type^11^.

Recent studies have revealed that TF binding is highly clustered in *Caenorhabditis elegans*^12^, *Drosophila melanogaster*^13^,^14^,^15^,^16^, and humans^10^,^17^. The broad presence of clustered transcription factor binding sites (TFBSs) in worms, flies, and humans suggests that they might represent a general property of regulatory genomes. However, the manner in which hundreds of TFs coordinate their binding in clusters across cell types and tissues remains unclear. Because TFBSs are hypersensitive to DNase I and are located in only a fraction of the human genome^18^, TF motif discovery at DHSs can greatly increase the speed with which TFs can locate their binding sites, and can significantly extend the repertoire of TFs in the human genome.

We have developed a computational method for the genome-wide mapping of TFBS-clustered regions in 133 human cell and tissue types. An integrative analysis using ENCODE data extended our understanding of these TFBS-clustered regions. Furthermore, the TFBS-clustered regions could be used to establish human lineage relationships. Based on these findings, we present a transcriptional regulation model of the accessible chromatin landscape as determined by TFBS complexity. We discuss the implications of this broad resource we have generated for future studies of the comprehensive assessment of transcription factor cooperativity in relation to human health and disease.

## Results

### Identification of TFBS-clustered regions across diverse human cells

**Figure 1:**
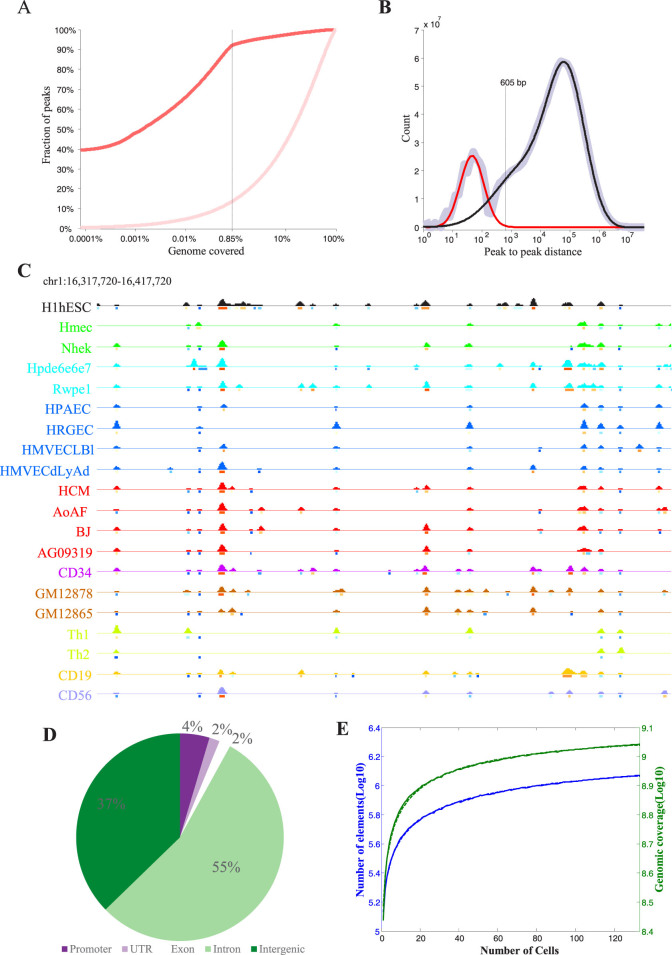
Identification of the human TFBS-clustered regions. (A) Most of TF-binding sites are concentrated in approximately 0.8% of the genome. Fraction of site-site intervals (*y* axis) as a function of fraction of genome covered (*x* axis) by the same intervals (red line). The distribution expected by random (light red) is shown for comparison. (B) Width determination of the TFBS clusters. The distribution of distances between each pair of TFBSs in the genome (light blue) can be modeled with the distributions representing site pairs within (red) and between (black) clusters. (C) The Gaussian kernel density across the binding profiles of the 542 TFs in 19 primary human cell types in addition to ESCs. A approximately 100 kb region along chromosome 1 is shown. The cell types are colored according to their embryological origin: black, ESC; green, ectoderm; aqua, endoderm; blue, endothelia; red, somatic mesoderm; magenta, hemat; brown, B-lymphocyte; light green, T-cell; gold, B-cell; periwinkle, NK-Cell. Lines under each profile indicate distinct TFBS complexity categories. (D) The distribution of 1,583,977 TFBS-clustered regions with respect to GENCODE annotations. (E) The saturation curves of TFBS-clustered regions with Weibull Fitting. Mean TFBS-clustered region count (blue line) and mean genome coverage (green line) for *x* cell types after clustering from 20,000 random samples (solid line), fit using the Weibull distribution (corresponding dashed line). The elements are non-overlapping and have maximum length 5000 bp. See also Figure S1 and Tables S1.

We produced high-quality genome-wide maps of the TFBSs for 542 TFs in 133 human cell and tissue types that were included in the ENCODE Project^19^. On average, we obtained approximately 4,470 TFBSs for each TF using iFORM (incorporating Find Occurrence of Regulatory Motifs) (Chen *et al.*, in preparation). To determine whether the binding sites were clustered together, we analysed a distribution of the distances between the adjacent binding sites in ESCs. Consistent with previous studies^10^,^12^,^13^,^17^, the TFBSs were highly clustered in distinct human cell types; 91% of the TFBSs were located in only 0.8% of the genome (Fig. 1A). To determine the average width of the TFBS clusters, the genomic distances between the adjacent TFBSs in the ESCs were plotted on a histogram (Fig. 1B). The distribution was clearly bimodal; short intervals were described well by a geometric distribution (mean 46 bp), and 99.5% of the predicted intervals were less than approximately 605 bp. This result suggests that TFBSs cluster in regions that are approximately 600 bp wide.

To identify the TFBS-clustered regions, we used a Gaussian kernel density estimation with a bandwidth of 300 bp to assay the binding profiles of the 542 TFs. We defined a “TFBS complexity” score based on the quantity and proximity of the contributing TFBSs (Figs. 1C and S1A). On average, we defined 141,846 TFBS-clustered regions per cell type (ranging from 62,092 to 315,831; Table S1) that spanned approximately 2.5% of the genome on average. Across all cell types, 1,583,977 distinct TFBS-clustered regions were discovered, collectively spanning 27.7% of the genome. These regions were predominantly detected in more than one cell type (median = 13; Fig. S1B). A majority (1,563,462; 98.7%) of the regions were bound by 2 or more two factors, while 20,515 (1.3%) regions were bound by a single TF. In addition, 56,316 (3.6%) regions were bound by more than 40 factors, and were thus classified as HOT (high-occupancy target) regions (Fig. S1C). Genome-wide location analysis showed that 25,767 (1.6%) of TFBS-clustered regions were found in UTRs as defined by GENCODE, 2.8% (72,877) of the regions were located in promoters, and 1.8% (28,360) of the regions were located in exons. Among the remaining TFBS-clustered regions, 54.7% (866,756) and 37.3% (590,217) of them were located within intronic and intergenic regions, respectively (Figs. 1D and S1D).

To determine whether our coverage of the TFBS-clustered regions was an underestimate, saturation analyses^19^ were performed to assess the rate of discovery of new TFBS-clustered regions. The saturation was predicted to be at approximately 1,696,566 (standard error (s.e.) = 692,615) of the TFBS-clustered regions and 1,243,240,105 (s.e. = 57,668,966) bp (40.9% genome coverage) (Fig. 1E). These saturation analyses indicated that nearly all (93%) of the total estimated number of TFBS-clustered regions had been discovered and that nearly 41% of the human genome is accessible to TF binding. These estimates represent a lower bound and support the observation that there are more non-coding functional DNA sequences than there are coding sequences or evolutionarily constrained bases in humans^19^.

### General features of the human TFBS-clustered regions

**Figure 2:**
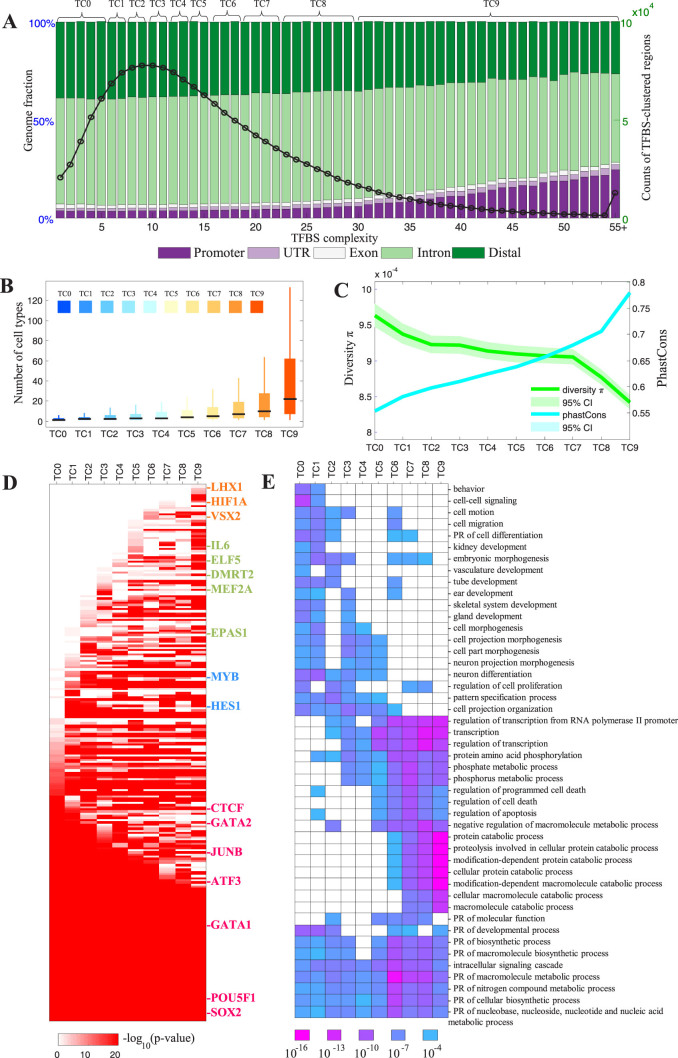
General features of the TFBS-clustered regions. (A) The number of TFBS-clustered regions (right *y*-axis, black circles) and distribution of genomic annotation classes (left *y*-axis, colors) as a function of TFBS complexity (*x*-axis). (B) The boxplot distributions of cell type number, from 1 to 133 (*y* axis), in each TFBS-clustered region category (*x* axis). (C) The distributions of PhastCons and nucleotide diversity in each TFBS-clustered region category. (D) The motif enrichment in each TFBS-clustered region category. (E) Gene ontology terms for genes associated TFBS-clustered regions of each complexity category with corresponding *p*-values. See also Figure S2 and Tables S1, S2, and S3.

To further characterise the TFBS-clustered regions, 10 categories of TFBS-clustered regions with increasing TFBS complexity were analysed. As TFBS complexity increased, the portion of the TFBS-clustered regions that were located within promoters (as defined by GENCODE^20^) increased, whereas the portion of the TFBS-clustered regions that were located within intergenic regions decreased (Fig. 2A). The categorisation also revealed that the TFBS-clustered regions exhibited an increase in cellular ubiquity with increasing TFBS complexity. The TFBS-clustered regions in the lowest complexity category were detected in 4 cell types. In contrast, the TFBS-clustered regions in the highest complexity category were detected in 39 cell types (Fig. 2B). An evolutionary conservation analysis of the categorised TFBS-clustered regions (i.e., the 10 categories of TFBS-clustered regions) revealed that sequence conservation increased and nucleotide diversity decreased with increasing TFBS complexity (Fig. 2C). This finding suggests that highly complex TFBS-clustered regions are functionally conserved and bear stronger signatures of purifying selection in humans.

To delineate the sequence features of the categorised TFBS-clustered regions, we used 796 motifs, representing the binding preferences of 542 human TFs, to determine the relative enrichment of TF-binding elements within the different categories of TFBS-clustered regions (Fig. 2D, Table S2). Forty six percent of the TFs (251 out of 542) were significantly enriched in at least one TFBS-clustered region category. Notably, over 42% (106 out of 251) of the enriched TFs, including ATF3, CTCF, POU5F1, SOX2, E2F6, JUNB, GATA1, and GATA2, were significantly enriched in all the categories of TFBS-clustered regions. The remaining enriched TFs demonstrated specificity for distinct TFBS-cluster region categories. For example, HES1, MYB, and KLF12 were specifically enriched in low-complexity TFBS-clustered regions, while DMRT2, MEF2A, NR0B1, ELF5, EPAS1, ZNF263, HOXA5, CDC5L, LHX8, IL6, MYBL2, and FOXJ1 were specifically enriched in median-complexity TFBS-clustered regions. Finally, LHX1, HOMEZ, FOXI1, LMX1A, HOXC4, HIF1A, and VSX2 were specifically enriched in high-complexity TFBS-clustered regions.

Next, we performed gene ontology analysis to characterise the genes associated with the TFBS-clustered region categories (Fig. 2E, Table S3). A small portion of overrepresented biological processes, largely defined by positive regulation, were significantly enriched in all the categories of TFBS-clustered regions. However, the most overrepresented biological processes were specific for distinct TFBS-cluster region categories. For example, the genes associated with low-complexity TFBS-clustered regions were involved in behaviour processes. The genes associated with median-complexity regions were associated with cellular development processes, and the genes associated with high-complexity regions were involved in transcription, phosphate metabolism, cell death, and metabolic processes. KEGG process analyses also demonstrated the specificity of the categorised TFBS-clustered regions (Fig. S2, Table S4). The low-complexity TFBS-clustered regions were associated with signalling molecules, interaction, and signal transduction, whereas the median-complexity regions were involved with the circulatory and endocrine systems and cell motility. The high-complexity regions were associated with protein folding, sorting and degradation, specific cancers, and the immune system.

### Transcription factor drivers of TFBS-clustered regions

**Figure 3:**
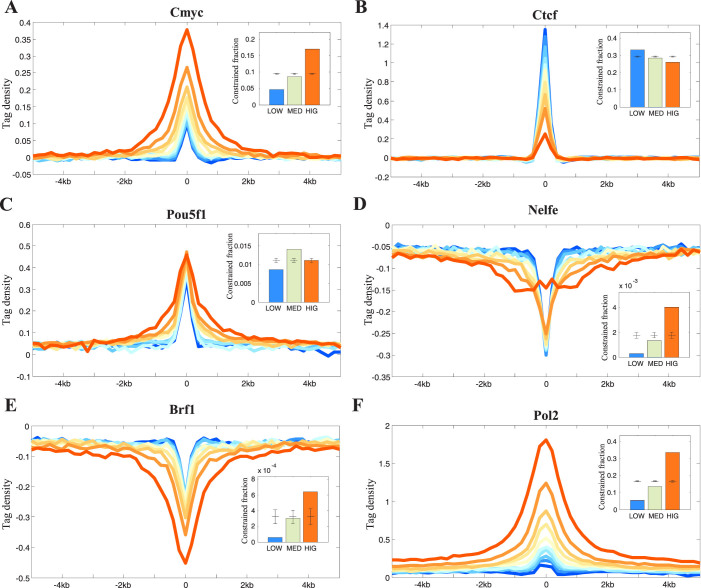
Transcription factor drivers of the TFBS-clustered regions. The profiles of transcription factor (A) CMYC (B) CTCF (C) POU5F1 (D) NELFE (E) BRF1 (F) POL II across the TFBS-clustered regions and their neighboring regions. Inset shows the GSC analysis of the TF peaks and TFBS-clustered regions. Bars indicate the fraction of low-, median-, and high-complexity TFBS-clustered regions that occupy TF peaks. Error bars are standard deviation for random placement of elements calculated with GSC. If columns are outside the standard deviation, TFBS-clustered regions are significantly associated with TF peaks. See also Figure S3 and Tables S5.

To quantify the occupancy patterns of the transcription factors in the TFBS-clustered regions, we considered 110 ENCODE transcription factors mapped by ENCODE ChIP-seq. Of these, 97 (88.2%) TFs were enriched in the TFBS-clustered regions, which contained a median value of 84% of the ChIP-seq peaks (Figs. 3 and S3 and Table S5). Several factors were found almost exclusively in the TFBS-clustered regions, including transcription activators AP2^21^, CTBP2^22^, and BRG1^23^. A small number of chromatin repressors diverged from this scenario, including known repressors such as ZNF274^24^, RFX5^25^ and MAFK^26^, suggesting that some factors may preferentially inhabit heterochromatin.

We further compared the aggregate ChIP-seq density profiles in the categorised TFBS-clustered regions. Among the enriched TFs within the TFBS-clustered regions, the greatest number of TFs demonstrated occupancy that increased along with increased complexity of the TFBS-clustered regions; these TFs included CMYC, P300, and E2F6 (Figs. 3A and S3A). In contrast, a small number of TFs showed decreased occupancy as the region complexity increased; these TFs included CTCF and RAD21^27^,^28^ (Figs. 3B and S3B). Interestingly, a few TFs showed their highest occupancies in the median-complexity TFBS-clustered regions (Figs. 3C and S3C), including the known pluripotent-cell master TFs NANOG, SOX2, and POU5F1^29^. With respect to TFs that were depleted within the TFBS-clustered regions, NELFE, MALF, and STAT1 showed increased occupancy that correlated with increased complexity (Figs. 3D and S3D), whereas BRF1, BRF2, POL3, and ZNF274 demonstrated decreased occupancy as complexity increased (Figs 3E and S3E). To further elucidate the patterns of transcription factor occupancy within each category of the TFBS-clustered regions, GSC (genome structure correction) analyses were performed between the TF peaks and the low-, median-, and high-complexity TFBS-clustered regions. The GSC results were highly consistent with the ChIP-seq aggregate density profiles (insets in Figs. 3 and S3 and Table S5). Collectively, these results suggest that the categorised TFBS-clustered regions illustrate distinct TF occupancy patterns.

### RNA polymerase II and RNA in the TFBS-clustered regions

RNA polymerase II, which can transcribe enhancers, produces noncoding RNAs that contribute to enhancer activity^30^,^31^,^32^,^33^,^34^. Thus, we measured the RNA polymerase II and RNA signals in the categorised TFBS-clustered regions to determine the effect of these regions on transcriptional control. Both the RNA polymerase II and RNA signals were highly enriched in each region category. As TFBS-clustered region complexity increased, the signals for RNA polymerase II and RNA also substantially increased (Figs. 3F and S3F). Additionally, the number of bound RNA polymerase II enzymes increased substantially with increasing region complexity (inset in Fig. 3F). These findings help to explain why high-complexity regions drive high-level expression of their associated genes relative to low-complexity regions. Our results demonstrate that high-complexity TFBS-clustered regions may be involved in regulating RNA polymerase II activity, and may therefore affect gene expression. They also indicate that high-complexity TFBS-clustered regions may harbour specific features that are associated with recently identified enhancer RNAs^30^,^31^,^32^,^35^, which are noncoding RNA transcripts that are produced from putative enhancer regions and are characterised by high levels of H3K4me1 and H3K4me2 relative to H3K4me3.

### Epigenetic signatures of the TFBS-clustered regions

**Figure 4:**
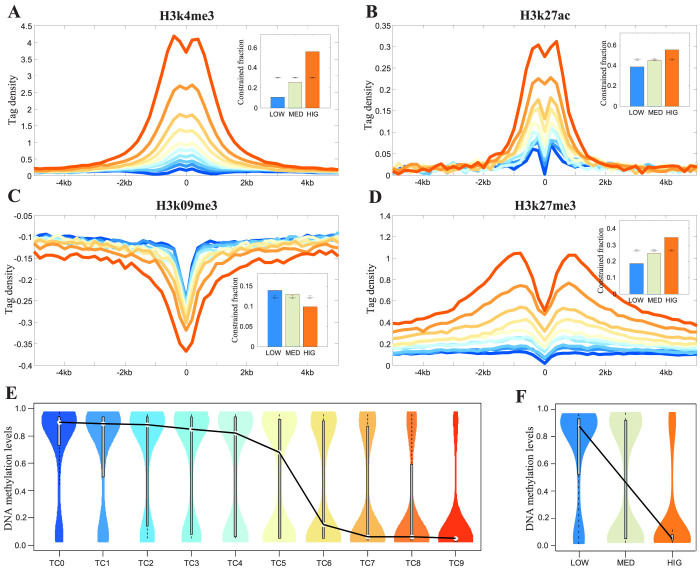
Epigenetic signatures of the TFBS-clustered regions. (A–D) The profiles of epigenetic markers (A) H3K4me3 (B) H3K27ac (C) H3K9me3 (D) H3K27me3 across the TFBS-clustered regions and their neighboring regions. Inset shows the GSC analysis of histone peaks and TFBS-clustered regions. Bars indicate the fraction of low-, median-, and high-complexity TFBS-clustered regions that occupy histone peaks. Error bars are standard deviation of random placement of elements calculated with GSC. If columns are outside the standard deviation, TFBS-clustered regions are significantly associated with histone peaks. (E) The violin distributions of DNA methylation levels within each TFBS-clustered region category. (F) The violin distributions of DNA methylation levels within low-, median-, and high-complexity TFBS-clustered regions. See also Figure S4 and Tables S6.

To further characterize the TFBS-clustered regions, 10 histone modifications (H3K4me1/me2/me3, H3K36me3, H3K27me3, H3K9me3, H3K79me2, H4K20me1, H3K9ac, and H3K27ac) and a histone variant (H2A.Z) were analysed in H1 ES cells (Figs. 4 and S4). These markers represent different types of chromatin activity. Aggregate ChIP-seq density profiles were compared within each categorised TFBS-clustered regions. The active-chromatin markers H3K4me1/me2/me3, H3K9ac, H3K27ac, and H2A.Z differentially increased with increasing TFBS complexity (Figs. 4A–B and S4A–D). Interestingly, a few repressive markers, such as H3K9me3 and H3K36me3, differentially decreased as TFBS complexity increased (Figs. 4C and S4E). Additional repressive histone marks, such as H3K27me3, H3K79me2, and H4K20me1, increased with increasing complexity (Figs. 4D and S4F–G). These findings are highly consistent with the GSC analysis, which revealed the enrichment and depletion patterns of these markers in the low-, medium-, and high-complexity TFBS-clustered regions (insets in Figs. 4 and S4A–G and Table S6).

The methylation of cytosine at CpG dinucleotides plays a vital role in diverse biological processes^36^. Thus, we integrated methylated DNA immunoprecipitation (MeDIP-seq) and methylation-sensitive restriction enzyme (MRE-seq) sequencing data from human H1 ESCs to determine the methylation levels of 28 million CpGs using MethylCRF^37^. This integrated analysis revealed that 3,122,813 (approximately 11.1%) CpGs occurred in TFBS-clustered regions, thereby covering approximately 4% of the genome. Although the density of the CpGs within the TFBS-clustered regions was much higher than in the genome-wide background (Fig. S4J; 15.58 vs. 7.00, two-sample Kolmogorov-Smirnov test, *p*-value = 10^−323^), the CpGs in the TFBS-clustered regions were significantly less methylated than in the genome at large (Fig. S4H; 0.08 vs. 0.90, two-sample Kolmogorov-Smirnov test, *p*-value = 10^−323^). Individual analysis of each TFBS-clustered region complexity category revealed that increasing density of CpGs was uniformly associated with increasing TFBS complexity (Fig. S4I). Interestingly, the methylation level was strongly and negatively correlated with TFBS complexity (*R*^2^ = 0.84, Figs. 4E and 4F). Therefore, TFBS-clustered regions are selectively protected from DNA methylation, and the magnitude of protection has a significantly positive association with TFBS complexity. Our results indicate that there is a widespread connection between TF binding levels that can be measured by TFBS complexity and DNA methylation, which confirms and extends recent reports^18^,^38^,^39^.

### Chromatin structure surrounding the TFBS-clustered regions

**Figure 5:**
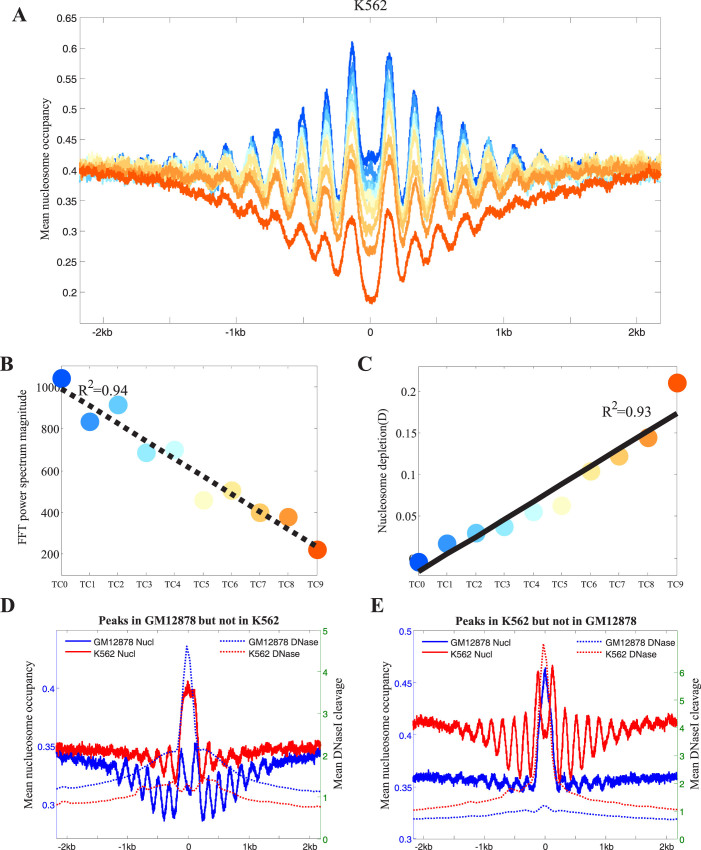
Chromatin structure of the TFBS-clustered regions. (A) The nucleosome occupancy profiles anchored on DHS centres in each TFBS-clustered region category (K562 cells). (B) The fast Fourier transform (FFT) spectra at the period of positioning across each TFBS-clustered region category (K562 cells). (C) The nucleosome depletion “D” across TFBS-clustered regions in each TFBS-clustered region category (K562 cells). (D) The nucleosome occupancy profiles (solid lines) and DNase I cleavage profiles (dashed lines) anchored on DHS centres within the GM12878-specific TFBS-clustered regions. (E) Nucleosome occupancy profiles (solid lines) and DNase I cleavage profiles (dashed lines) anchored on DHS centres within the K562-specific TFBS-clustered regions. See also Figure S5.

Recent studies have reported that CTCF and NRSF (also called REST) binding sites are flanked by strongly positioned nucleosomes; they appear as a periodic oscillatory pattern in the average nucleosome occupancy profile centred on binding sites^40^,^41^,^42^. To investigate the chromatin structure surrounding the TFBS-clustered regions, we computed the average nucleosome occupancy profile when separately anchored on each category of TFBS-clustered regions (Figs. 5 and S5). We found that the categorised TFBS-clustered regions showed striking patterns of positioned flanking nucleosomes. We distinguish between nucleosome positioning and nucleosome occupancy, as described in a recent study^43^. To quantify the regularity of the nucleosome positioning surrounding the categorised TFBS-clustered regions, fast Fourier transforms (FFTs) were applied to the nucleosome occupancy profiles, yielding power spectra. The periodicity of each nucleosome position was determined by the height of the power spectrum at the spatial frequency corresponding to the nucleosomal repeat length. Our analysis revealed that the power spectrum height correlated negatively with TFBS complexity (*R^2^* = 0.94, Figs. 5B, S5B, S5D, and S5E). Furthermore, the nucleosome occupancy profile dips at the TFBS-clustered regions (Figs. 5A and S5A), indicating that TFs preferentially bind to nucleosome-depleted regions or that TF binding excludes nucleosomes. We define nucleosome depletion as a nucleosome occupancy profile that dips at the centre of the TFBS-clustered regions relative to the nucleosome occupancy profile 2 kb from the centre (considered to be background). The high-complexity TFBS-clustered regions showed significantly greater nucleosome depletion than the low-complexity regions (Figs. 5A and S5A). As TFBS complexity increased, nucleosome depletion showed a significantly positive linear correlation with TFBS complexity (*R*^2^ = 0.93, Figs. 5C and S5C). These results indicate that TFs and nucleosomes compete for genomic DNA and that lower-complexity TFBS-clustered regions are correlated with more strongly-positioned periodical nucleosomes and with greater nucleosome occupancy, above and beyond the effect of transcription.

Two sets of cell-type-specific TFBS-clustered regions were analysed to investigate the relationship between TFBS-clustered regions and chromatin structure. The first was detected in GM12878 cells but not in K562 cells, and the second was detected in K562 cells but not in GM12878 cells. Nucleosome occupancy and DNase I cleavage profiles anchored on the centres of the two sets were determined separately for each cell line (Figs. 5D–E). The GM12878-specific TFBS-clustered regions showed a decrease in nucleosome positioning in the K562 cells, and vice versa (power spectra: 38.3 vs. 1.5 in GM12878 cells; 118.9 vs. 18.3 in K562 cells). Additionally, the GM12878- and K562-specific TFBS-clustered regions were preferentially occupied by nucleosomes in the K562 and GM12878 cells, respectively. Accordingly, increased nucleosome occupancy manifested as decreased DNase I cleavage in the K562 and GM12878 cells. Similar results were obtained when each TFBS complexity category of the GM12878- or K562-specific TFBS-clustered regions was assayed (Figs. S5F-U). Collectively, our results show that there is a strong correlation between TFBS complexity and the positioning and occupancy of nucleosomes. Such a correlation is likely a universal phenomenon that can be regulated in a cell-type-specific fashion.

### Lineage programming of human TFBS-clustered regions

One recent study indicated that developmental fate and lineage relationships were derived from DHSs in definitive cells^44^. TFBS-clustered regions are similar to DHSs in that both are both highly cell selective and highly stable (Fig. S6A)^18^. This similarity suggests that developmental fate and lineage programming can also be derived from comparisons of TFBS-clustered regions in cell type pairs. Genome-wide maps of TFBS-clustered regions were generated from human ESCs, from 29 diverse normal definitive primary cell types and from 17 well-characterised hematopoietic cell types (Fig. S1A)^18^. We considered each TFBS-clustered region to be either present or absent within a given cell type and defined the similarity measure *Φ(X,Y)* as the Euclidean distance between all the pairs of cell types *X* and *Y*. Two precursor cells of these lineages, CD34 and H1 ESCs, enabled the study of branch points.

**Figure 6:**
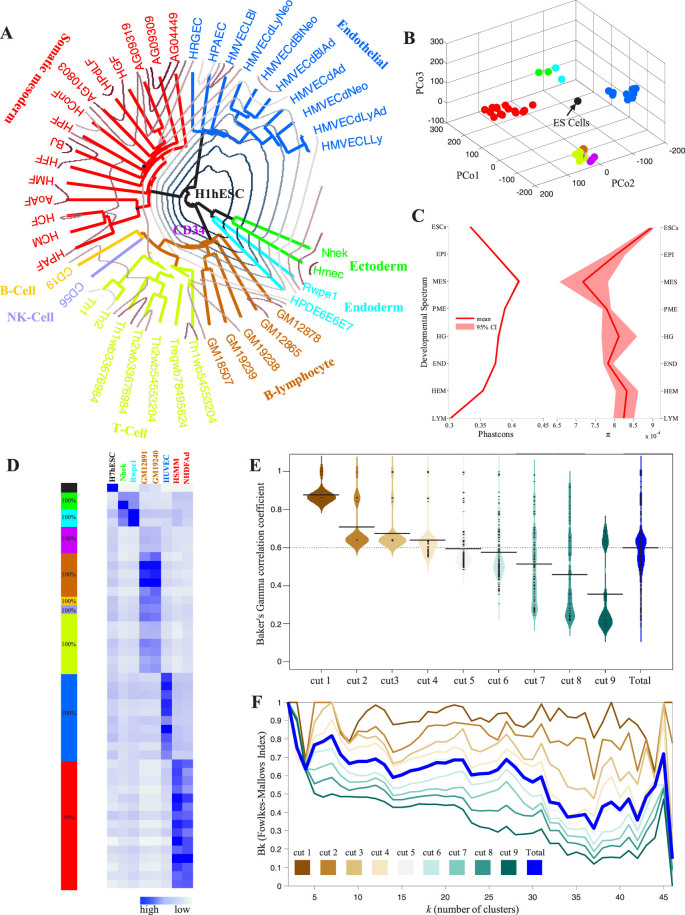
Lineage programming of the human TFBS-clustered regions. (A) The pairwise lineage relationships between 47 cell types based on the similarity measure *Φ*. The circular dendrogram in the *xy* plane arranges cells to branching lineages, which are colored according to their embryological origin. Precursor cell types placed to branch points with the Hungarian algorithm are indicated. The landscape elevation represents the similarity *Φ* to ESCs. (B) PCoA of cell-type relationships. Each cell type from (A) is projected into a 3D principal coordinates space. Cell-type coloring is indicated above. Note the centrality of ESCs and the spatial separation of major lineage groups. (C) “Hourglass” evolutionary pattern of TFBS-clustered regions across the developmental spectrum. Mean and 95% CIs of evolutionary conservation (left) and nucleotide diversity π (right) are shown. Abbreviations: Epiblast (EPI), mesoderm (MES), paraxial mesoderm (PME), hemangioblast (HG), endothelia (END), hematopoietic (HEM), and lymphoid (LYM). (D) Robustness analysis of the clustering. (letf) Bootstrap analyses of major branches shown in Figure 6A. Displayed is the percentage of 1,000 bootstrapped dendrograms, which is colored according to their embryological origin. (right) Addition of any of the cell types yields the major branches shown in Figure 6A. The euclidian distance of the TFBS-clustered regions of the 8 additional cell types versus all 47 original cell types as measured by overlapping TFBS-clustered regions. (E–F) Sensitivity analysis of the clustering. Distribution of Baker's Gamma γ correlation coefficient (E) and *B_k_* plot (F) at each subset of TFBS-clustered regions. See also Figure S6.

Classical hierarchical clustering approaches create dendrograms; however, dendrograms cannot reflect the biological lineage tree, as the precursors are placed on the leaves rather than on the branch points. Thus, we performed hierarchical clustering based on the similarity *Φ* in 47 terminally differentiated cells, and we then separately place precursor cell types onto the tree branch points using the Hungarian algorithm (HA)^45^. The resulting *ab initio* dendrogram reflects the established hierarchical lineage relationships among these cell types (Figs. 6A and S6B). H1 ESCs occupied the deepest root, while the mesoderm, ectoderm, and endoderm were correctly partitioned into separate high-level clusters (Fig. 6A). The mesodermal progeny were divided into paraxial mesoderm, primitive mesoderm, and hemangioblast derivatives. The embryological origin of endothelia and blood was also revealed. Furthermore, the hematopoietic progeny were partitioned into hematopoietic progenitors, lymphoid cells, and myeloid cells. They were also partitioned into subtypes of lymphoid tissue, including B cells, T cells, NK cells, and the more primitive lymphoblastoid cells. A three-dimensional (3D) principal coordinate analysis (PCoA) further confirmed the distinctiveness of these major cluster groups and the central positions of the ES TFBS-clustered regions (Fig. 6B).

To determine whether the lineage relationships that were derived from the simple clustering of the TFBS-clustered regions in differentiated cells coincided with evolutionary constraint patterns, a recent method^44^ was used to identify the TFBS-clustered regions that stably arose at the seven developmental branch points, namely the epiblast, mesoderm, paraxial mesoderm, hemangioblast, endothelia, hematopoietic, and lymphoid branch points. Two measures of sequence evolution, conservation and constraint, were used to calculate the mean evolutionary levels of the eight lineage-restricted groups (Fig. 6C). The TFBS-clustered regions that arose stably in the mesodermal lineage demonstrated the highest levels of evolutionary conservation/constraint, and the regions that arose either during early embryogenesis or later lineage differentiation showed reduced levels of conservation/constraint, suggesting that mesodermal derivatives are subject to stronger purifying selection.

We applied bootstrap analysis^46^ to confirm the stability of the lineage relationships exposed by clustering the TFBS-clustered regions. This analysis showed that the bootstrapped dendrograms retained all the major branches (Fig. 6D, left). To further confirm the robustness of the clustering, we added multiple cell types, including ESC (*n* = 1), B-lymphocyte (*n* = 2), somatic mesoderm (*n* = 2), endoderm (*n* = 1) and ectoderm (*n* = 1). The addition of any of these cell types yielded a dendrogram that was almost identical to the dendrogram obtained with all 47 cell types (Fig. 6D, right; Fig. S6C).

To determine whether the dendrogram we obtained could be systematically reproduced without the TFBS-clustered region categories, an ensemble of 1,022 data sets with at least one TFBS-clustered region category left out was generated from all 47 differentiated cell types. Similar *Φ* construction and clustering procedures to those shown in Fig. 6A were performed on these data sets. We used Baker's Gamma γ^47^ and a *B_k_* plot^48^ to compare the similarity between the experimental dendrograms and the reference dendrogram from the complete data set. The median γ value was approximately 0.90 after removing a single category. However, as more categories were removed, the median values of γ dropped dramatically (Fig. 6E). The ensemble of all 1,022 *B_k_* plots was illustrated, and each *B_k_* plot showed significant similarities between the experimental dendrograms and the reference dendrogram, as the points generally lay well beyond the limit *E*(*B_k_*) ± 2(var(*B_k_*))^1/2^. Furthermore, *B_k_* was quite symmetric for 5 ≤ *k* ≤ 30. Although *B_k_* reached 1 at some levels of *k*, no incomplete group of categorised TFBS-clustered regions completely replicated the reference dendrogram based on the complete data set (Figs. 6F and S6D-M). This result suggests that the clustering is sensitive to the categorised TFBS-clustered regions.

## Discussion

Here, we present by far the most comprehensive catalogue to date of human TFBS-clustered regions. It was generated via genome-wide mapping of the TFBSs in 133 human cell and tissue types using a computational method based on Gaussian kernel density estimation. We identified approximately 1.6 million distinct TFBS-clustered regions spanning 27.7% of the human genome. Saturation analyses suggested that nearly all of the estimated TFBS-clustered regions were discovered in our analysis and that approximately 41% of the human genome is accessible to TF binding. Although these estimates are conservative, they further reinforce the finding that the quantity of functional non-coding DNA sequences exceeds that of coding sequences or of evolutionarily constrained bases in humans.

Partitioning the TFBS-clustered regions according to their TFBS complexity revealed that distinct TFBS-clustered region categories represent differential genomic location, cell specificities, evolutionary conservation, sequence features, and functional roles. Our results suggest that the human accessible chromatin landscape is generally organised into large TFBS-clustered regions with distinct levels of TFBS complexity that are characterised by specific combinations of genomic signatures and play different functional roles.

**Figure 7:**
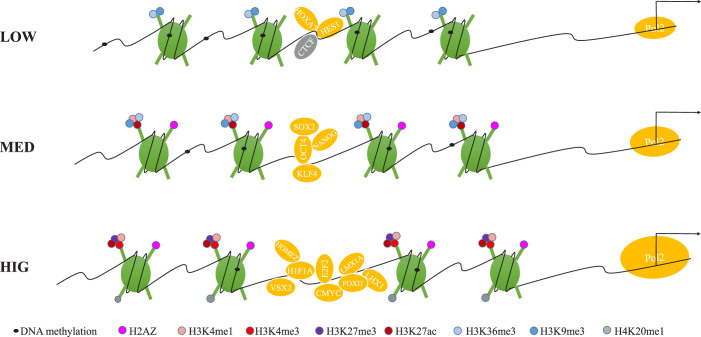
Transcriptional regulation models on the accessible chromatin landscape. Transcriptional regulation models summarizing the main results presented. The TFBS-clustered regions with low-, medium-, and high-complexity demonstrate differential and characteristic transcription factor occupancy, transcriptional activity, histone modification, DNA methylation, and nucleosome occupancy.

Further integrative analyses using ENCODE data were performed to extend our understanding of the TFBS-clustered regions by determining TF occupancy patterns, transcriptional activity, and chromatin signatures, including histone modification, DNA methylation, and chromatin structure. These analyses led us to propose that TFBS complexity determines TF occupancy, transcriptional activity, and chromatin structure (Fig. 7). Indeed, the low-complexity TFBS-clustered regions were characterised by the presence of CTCF, HOXA7, and HES1, low transcriptional activity, the repressive histone modifications H3K9me3 and H3K36me1, high DNA methylation levels, maximal nucleosome occupancy and the strongest periodic nucleosome positioning. In contrast, the high-complexity TFBS-clustered regions were characterised by the presence of CMYC, E2F2, VSX2, LMX1A, HOMEZ, H1F1A, and FOXI1, high transcriptional activity, both active and repressive histone modifications such as H2AZ, H3K4me1, H3K4me3, H3K27me3, H3K27ac and H4K20me1, low DNA methylation levels, minimal nucleosome occupancy and the weakest periodic nnucleosome positioning. The median-complexity TFBS-clustered regions contained several master transcription factors associated with pluripotent cells, including POU5F1, NANONG, SOX2, and KLF4^29^, and showed the histone modifications H3K36me3, H3K9me3, H3K4me1, H3K4me3, and H2A.Z. The strong correlations found between TFBS complexity and TF occupancy, transcription levels, chromatin modification, and chromatin structure support the notion that chromatin structure changes to accommodate TFs and the passage of RNA polymerase around transcriptionally genes^49^ and suggest that TFs cooperate with chromatin modifiers and remodellers to determine chromatin structure.

Hierarchical clustering of the TFBS-clustered region maps from human ESCs in addition to 46 other primary cell types revealed that the TFBS-clustered regions reflected human lineage hierarchies that were consistent with the established lineage relationships derived from DHSs in definitive cells^44^. We emphasise that this clustering is purely data-driven; we do not incorporate any knowledge other than the categorised TFBS-clustered regions. The developmental patterns revealed by the dendrogram resembled the evolutionary patterns observed in Fig. 6C, supporting the “hourglass” model of development^50^,^51^ described by cross-species morphology^52^, gene expression^53^, and gene conservation^54^. This resemblance indicates that, as with the regulatory DHS landscape, the hourglass phenomenon may be related to TFBS-clustered regions^44^. Stability and sensitivity analyses suggest that developmental patterning is generally robust, whereas clustering results vary across the TFBS-cluster region categories.

The data described herein greatly strengthen our understanding of the mechanisms underlying transcriptional regulation in the human genome. This study, and particularly the massive data resources that it introduces, are expected to promote future research and encourage new explorations into the comprehensive assessment of transcriptional regulation in relation to common phenotypes, especially those involved in human health and disease. There are at least two types of TFBS-clustered regions that warrant further research. HOT regions are defined as TFBS-clustered regions with extremely high TFBS complexity. Previous studies^10^,^12^,^13^,^14^,^15^,^16^,^17^ have revealed many HOT regions in metazoan genomes. However, the function of HOT regions has remained unclear, and their proposed roles include putative functions as mediators of ubiquitously expressed genes^12^, insulators^14^, DNA origins of replication^14^, sinks or buffers for sequestering excess TFs^16^, and patterned developmental enhancers^70^. Alternatively, “COLD” regions are defined as TFBS-clustered regions with extremely low TFBS complexity. “COLD” regions have been identified in *Drosophila melanogaster*^70^,^71^, *Caenorhabditis elegans* and humans^72^, however, their function remains unknown. Our research lays a solid cornerstone for investigating the functions of HOT and “COLD” regions and for exploring their comprehensive association with human health and disease.

## Methods

### Data sets

DNaseI Hypersensitivity by Digital DNaseI data were obtained from Duke and UW ENCODE groups. Histone modifications by ChIP-seq data were download from the Broad histone ENCODE group. Transcription factors by ChIP-seq data were obtained from the HAIB and SYDH TFBS ENCODE groups. MNase-seq data were obtained from Stanford and BYU ENCODE groups. Gene annotations were obtained from the GENCODE data (V15). All these data were provided through the ENCODE Project^19^, and the use of the data strictly adheres to the ENCODE Consortium Data Release Policy. PhastCons were extracted from the hg19 conservation track of the UCSC Genome Browser^55^. MeDIP-seq and MRE-seq data from H1 ESC cells were obtained from the NIH Roadmap Epigenomics Mapping Centers' repository for human reference epigenome atlas^56^.

### DNase-seq data analysis

DNase-seq data were processed using a uniform processing pipeline as described in the ENCODE integrative analysis study^19^. For each sample, the sequence reads were mapped using Bowtie^57^, allowing for a maximum of two mismatches. Only those reads that mapped uniquely to the genome were utilized in the analysis. All of the replicates for a given cell/tissue type were combined and subsampled at the level of 30 million tags. We identified the DNaseI hypersensitive regions of accessible chromatin (hotspots) and DHSs with a false discovery rate (FDR) threshold of 1% using the hotspot algorithm^58^. Our methods were applied uniformly to the data sets from 224 samples, including 133 cell types that were studied in the ENCODE Project^19^ (Table S1).

### Identification of the TFBSs

The position-specific weight matrices of 542 TFs, which corresponded to 796 motif models, were collected from TRANSFAC^59^, JASPAR^60^, and UniPROBE^61^ databases. We used the genomic sequences under the DHSs in the hg19 genome as inputs for iFORM (Chen et al., in preparation) with a custom library of all 796 motifs to scan for motif instances at a *p*-value threshold of 10^−18^ (corresponding to an FIMO threshold of 10^−5^). The motif instances were combined to generate the TFBSs for each TF.

### Identifying TFBS-clustered regions in human cells

An established method^14^ was used to perform the Gaussian kernel density estimations across the genome (with a bandwidth of 300 bp centred on each TFBS). Each peak in the density profile was considered a TFBS-clustered region. To determine the complexity of each TFBS-clustered region, the Gaussian kernalised distances from each peak to each TFBS that contributed at least 0.1 to its strength were determined. The window for each TFBS-clustered region was determined by finding the maximum distance (in bp) from the TFBS-clustered region to a contributing TF and then adding 150 bp (one-half of the bandwidth). Each window was centred on the TFBS-clustered region.

### Categorisation of the TFBS-clustered regions

To delineate the TFBS-clustered regions, we divided them into 10 categories based on their TFBS complexity. Categories 1 to 9, represented as TC0 to TC8, had TFBS complexity values of less than 6, 8, 10, 12, 14, 16, 19, 23, and 30, respectively. Category 10 (TC9) comprised all the regions with complexity values greater than 30. The TFBS complexity thresholds for each category were selected to maintain consistency and comparability (Table S7). Categories TC0 to TC2 were designated as low-complexity TFBS-clustered regions; categories TC3 to TC7 were designated as median-complexity TFBS-clustered regions; and categories TC8 to TC9 were designated as high-complexity TFBS-clustered regions.

### Saturation Analysis

Saturation analyses were revised from previous methods^19^. The TFBS-clustered regions were sorted, and their overlapping regions were combined. The cell type coverage was compared to 20,000 randomly generated cell type combinations for each coverage value. Thus, the distribution of the number of unique elements for any number of cell types is an approximation. This distribution was modelled using a Weibull distribution; hence, it was interpolated.

### Generation and annotation of a TFBS-clustered region master list

The TFBS-clustered regions from all the cell types were consolidated into a master list of 1,583,977 unique, non-overlapping TFBS-clustered region positions by merging the regions across cell types. From each resulting interval of merged sites, the TFBS-clustered region with the highest TFBS complexity was selected for the master list. The TFBS-clustered regions that overlapped with the regions selected for the master list were then discarded. The remaining TFBS-clustered regions were merged, and the process was repeated until each original TFBS-clustered region was either incorporated into the master list or discarded.

We used GENCODE annotations (V15)^20^, i.e., Basic, Comprehensive, PseudoGenes, 2-way PseudoGenes, and PolyA Transcripts, to annotate the master list. The “promoter” class for each GENCODE annotated TSS was defined as a peak in the master list within 1 kb of the TSS. The “exon” class was defined as any TFBS-cluster region outside the promoter class that overlapped a GENCODE-annotated “CDS” segment by at least 75 bp. The “UTR” class was defined as any TFBS-cluster region outside the promoter or exon classes that overlapped a GENCODE-annotated “UTR” segment by at least 1 bp. The “intron” class was defined as those GENCODE segments that were annotated as “gene” with complete “CDS” segments. The intron class also covered the TFBS-clustered regions that were not defined by other categories but that overlapped with introns by at least 1 bp.

The cell-type number was defined for each TFBS-cluster region by annotating the master list with the number of cell-types with overlapping TFBS-cluster regions. The plots in Fig. 2B were generated using the R function “boxplot” from the “boxplot” package; the plots summarise the distribution of cell-type numbers for distinct categories of TFBS-clustered regions. The distribution of the cell types that contained a TFBS-clustered region was calculated separately for the TFBS-clustered regions observed in 47 terminally differentiated cell types, 15 paraxial mesoderm cell types, 14 lymphoid cell types, and 10 endothelial cell types.

### Evolutionary conservation analysis

Two related measures of sequence evolution, conservation and constraint, were used to calculate the mean evolution levels of the categorised TFBS-clustered regions. We used phastCons to estimate the sequence conservation scores^62^ of multiple alignments of 45 vertebrate genomes to the human genome. We calculated constraint, which is measured by human nucleotide diversity (π) by using the genomic sequence data released by Complete Genomics (version 1.1034) from 53 unrelated individuals, as previously described^63^. Nucleotide diversity provides a quantitative assessment of ongoing purifying selection on the TFBS-clustered regions within the human population. To obtain a per-nucleotide estimate, π was normalised to the total number of bases under consideration for each particular analysis^63^. RepeatMasker regions, Gencode exons and CpGs were removed from all π calculations.

### Motif analysis

To locate enriched sequence motifs in the categorised TFBS-clustered regions, we analysed the genomic sequence under the DHSs within TFBS-clustered regions. HOMER (http://homer.salk.edu/homer/)^64^ was used with its default parameters to determine whether any of the 542 nonredundant TFs from TRANSFAC^59^, JASPAR^60^, and UniPROBE^61^ were overrepresented in any of the TFBS-clustered region categories. Overrepresentation was statistically evaluated using three independent background sets: chromosome 20, the complete set of RefSeq transcription start sites (TSSs) (±2.0 kb), and the complete set of CpG islands annotated in the hg19 genome. A motif was retained only when it was significantly overrepresented (*P* ≤ 0.01) compared with the background sets.

### Gene ontology analysis

Each TFBS-clustered region was assigned to the closest GENCODE (V15)-annotated genes by determining the distance from the centre of the TFBS-clustered region to the TSS of each GENCODE gene. The genes associated with each TFBS-clustered region category were analysed using DAVID^65^ for gene ontology (GO) analysis. For each region category, the 10 top-scoring categories with the lowest *p*-values were selected for display. A threshold *p*-value score of 10^−4^ was incorporated as a minimum requirement filter for the top category.

### Density of the ChIP-seq data surrounding the TFBS-clustered regions

The genome-wide ChIP-seq density of transcription factor and histone modifications surrounding the TFBS-clustered regions in each category (Figs. 3, 4, S3 and S4) was estimated by mapping the reads to the ±5 kb flanking regions of the centres of the TFBS-clustered regions. The flanking regions were split into 50 equally sized bins, which were aligned at the centre. The average ChIP-seq density in each bin was calculated to create a genome-wide average in terms of reads per million per base pair (rpm/bp).

### Statistical analysis by GSC

The GSC statistic^66^,^67^ was used to calculate the confidence intervals (CIs) for the transcription factor and histone modification peaks that were expected to contain diverse TFBS-clustered regions by chance. This statistic corrected for internal correlations of size and position within the annotations and within each TFBS-clustered region category.

### Characterisation of the chromatin structure surrounding the TFBS-clustered regions

Average nucleosome occupancy profiles were determined for each TFBS-clustered region category. Two sets of cell line-specific regions (one set found in GM12878 cells but not in K562 cells, and the other set found in K562 cells but not GM12878 cells) were constructed. The average nucleosome occupancy profiles and DNase I cleavage profiles anchored on the DHS centres of these cell line-specific regions were then determined.

Nucleosome depletion was defined as a dip in nucleosome occupancy, which was found by comparing the background signal to the signal at the centre of each region. A fast Fourier transform (FFT) was applied to the nucleosome occupancy profile. The magnitude of the FFT power spectrum at the frequency component corresponding to the period of the positioned nucleosomes was used to indicate the strength of nucleosome positioning (the higher the magnitude, more periodic the nucleosome occupancy profile). The frequency component of the power spectrum (*x*-axis) corresponds to all the possible periods that may exist in the input signal.

### Clustering the TFBS-clustered regions in noncancerous human cells

A newly developed method^44^ was used to cluster the TFBS-clustered regions in noncancerous cells. Briefly, a final reference set comprising the unique TFBS-clustered regions in noncancerous cell types was constructed using the BEDOPS suite^68^, version 2.2.0 (using bedops -u). Similarity, *Φ(X,Y)*, was defined as the Euclidean distance between cell type pairs *X* and *Y*, which can be calculated using vectors of binary values with sizes equal to the number of elements in the reference set. For each element in the reference set, a cell type received a “1” if it contained a TFBS-clustered region enveloped by the reference element; otherwise, it received a “0” (using bedmap–fraction-map 1–indicator). Pairwise Euclidean distances were computed and arranged into a matrix. Hierarchical clustering based on the similarity *Φ* in all 47 terminally differentiated, non-redundant cell types was performed with the nearest-neighbour algorithm. Another recently developed method^69^ was used to assign two naive cell types (CD34 and H1 ESCs) as precursors by taking *Φ* into consideration. The Hungarian algorithm (HA)^45^ was used to determine the optimal assignment for each progenitor cell type. Multidimensional scaling was used to construct a two-dimensional (2D) representation of the similarity matrix. A landscape was interpolated over the 2D representation of the cell types using the similarity *Φ* to the ESC as the elevation. Additionally, three dimensional plots were generated with the cmdscale function in R.

### Stability and sensitivity analysis of cell-type clustering

A 1000-iteration bootstrap approach^46^ was used to test the stability of the clustering results. Each iteration consisted of randomly sampling the genomic positions from the reference set with replacement until the number of positions obtained was equal to that of the reference set. A new clustering result was generated for each sample, calculated as the percentage of times that each branch remained unchanged compared with the reference set.

The sensitivity of our clustering results was determined by generating an ensemble of the data sets with at least one TFBS-clustered region category left out. Subsets of the categorised TFBS-clustered regions (1022 data sets) were generated for all 47 non-redundant cell types. Calculation of the similarity *Φ* and the hierarchical clustering procedure were applied to all the data sets, as shown in Fig. 6A. Baker's Gamma γ^47^ index, which is the rank correlation between the stages at which pairs of objects combine in each of two trees, was used to measure the similarity between two hierarchical clustering trees. The Fowlkes-Mallows index, *B_k_* (*k* = 2, …, *n*-1; *n* = 47 cell types)^48^, was used to measure the similarity or faithfulness between the dendrograms. *B_k_* versus *k* was plotted for each set of two hierarchical clustering dendrograms. The *B_k_* plot helps to identify the similarity between two dendrograms at different values of the number of clusters *k*, and it can be enhanced by the addition of *E*(*B_k_*) and the limits *E*(*B_k_*) ± 2(var(*B_k_*))^1/2^. If *B_k_* falls outside these limits, the similarity is considered to be significant.

### Evolutionary Conservation of TFBS-clustered regions arising in Embryological Ancestors

Following recently described method^44^, eight lineage-restricted groups of the TFBS-clustered regions across the developmental spectrum were defined, including epiblast, mesoderm, paraxial, hemangioblast, endothelia, hematopoietic, and lymphoid lineage group. We used conservation and constraint to calculate the mean evolution levels of the TFBS-clustered regions within lineage groups. For each TFBS-clustered region within a lineage group the maximum evolution level of 100 bp window within this region was identified. For each group, 1,000 values were sampled with 1,000 replacements to calculate the average evolution level and 95% confidence intervals.

### Accession numbers

The identified TFBSs and TFBS-clustered regions have been deposited with the Gene Expression Omnibus under the accession ID GSE53962 and GSE59016.

## Author Contributions

W.S. conceived the project. S.W., X.B. and W.S. designed all experiments. H.C., H.L., F.L. and X.Z. performed the experiments. All authors analysed the data and contributed to manuscript preparation. W.S. wrote the manuscript.

## Supplementary Material

Supplementary Information Supplementary materials
