# Supplementary material for: An integrative analysis of TFBS-clustered regions reveals new transcriptional regulation models on the accessible chromatin landscape
Source: Sci Rep. 2015 Feb 16;5:8465. doi: 10.1038/srep08465 (PMC4329551; doi:10.1038/srep08465)
Supplement: Supplementary Information — Supplementary materials [file srep08465-s1.pdf]

Supplementary materials for

An integrative analysis of TFBS-clustered regions reveals new transcriptional regulation models on the accessible chromatin landscape

Running Title: New transcriptional regulation models on the accessible chromatin landscape

Hebing Chen[1], Hao Li[1], Feng Liu[1], Xiaofei Zheng[2], Shengqi Wang[1*], Xiaochen Bo[1*], Wenjie Shu[1§]

[1]Department of Biotechnology, Beijing Institute of Radiation Medicine, Beijing 100850, China

[2]Department of Biochemistry and Molecular Biology, Beijing Institute of Radiation Medicine, Beijing 100850, China

[*]Corresponding author. To whom correspondence should be addressed. Tel & Fax: +86 10 68210077 66932211; Email: shuwj@bmi.ac.cn. Correspondence may also be addressed to: boxc@bmi.ac.cn and sqwang@bmi.ac.cn.

Supplementary Figures

Figure S1. Identification of the TFBS-clustered Regions across diverse human cells, related to Figure 1

(A) Gaussian kernel density across the binding profiles of 542 TFs for 46 definitive cell types plus embryonic stem cells. A ~100 kb region along chromosome 1 is shown. Cell types are colored according to their embryological derivation. Lines under each profile indicate distinct TFBS-clustered region categories. (B) Distributions of cell types, from 1 to 133 (*x* axis), in which TFBS-clustered regions (*y* axis) are observed. (C) Distributions of TFBS complexity, from 1 to 55 (*x* axis), in which TFBS-clustered regions (*y* axis) are observed. (D) Distribution of intergenic TFBS-clustered regions relative to GENCODE TSSs.

See also Tables S1.

Figure S2. KEGG analysis for the TFBS-clustered region, related to Figure 2

KEGG analysis for TFBS-clustered-region-associated genes in each TFBS complexity class in 52 human cell and tissue types with corresponding *p*-values.

See also Tables S4.

Figure S3. Transcription factor drivers of the TFBS-clustered regions, related to Figure 3

Profiles of epigenetic markers across TFBS-clustered regions and their neighboring regions. (A) Positive enrichment of TFs. (B) Negative enrichment of TFs. (C) Positive enrichment of TFs within median complexity TFBS-clustered regions. (D) Positive depletion of TFs. (E) Negative depletion of TFs. (F) RNA-seq. Inset shows GSC analysis of TF peaks and TFBS-clustered regions. Bars indicate fraction of low, median, and high complexity TFBS-clustered regions that occupy TF peaks. Error bars are standard deviation for random placement of elements calculated with GSC. If columns are outside the standard deviation, TFBS-clustered regions are significantly associated with TF peaks.

See also Tables S5.

Figure S4. Epigenetic signatures of the TFBS-clustered regions, related to Figure 4

Profiles of epigenetic markers across TFBS-clustered regions and their neighboring regions. (A) H3K4me1. (B) H3K4me2. (C) H3K9ac. (D) H2A.Z. (E) H3K36me3. (F) H3K79me3. (G) H4K20me1. Inset shows GSC analysis of histone peaks and TFBS-clustered regions. Bars indicate fraction of low, median, and high complexity TFBS-clustered regions occupying histone peaks. Error bars are standard deviation for random placement of elements calculated with GSC. If columns are outside the

standard deviation, TFBS-clustered regions are significantly associated with histone peaks. (H) Violin distributions of methylation level in TFBS-clustered regions and Genome background. (I) Violin distributions of CpG density in each TFBS-clustered region category. (J) Violin distributions of CpG density in TFBS-clustered regions and Genome background.

See also Tables S6.

Figure S5. Chromatin structure of the TFBS-clustered regions, related to Figure 5

(A) Nucleosome occupancy profiles anchored on DHS centers within TFBS-clustered regions in each TFBS-clustered region category (GM12878 cells). (B) Fast Fourier transform (FFT) spectra at the period of positioning across TFBS-clustered regions in each TFBS-clustered region category (GM12878 cells). (C) Nucleosome depletion "D" across TFBS-clustered regions in each TFBS-clustered region category (GM12878 cells). (D-E) Fast Fourier transform spectra that correspond to the nucleosome occupancy profiles in K562 (D) and GM12878 (E). The regularity of positioning is shown as the magnitude of the FFT spectra at the period of positioning. (F-G) Nucleosome occupancy profiles in GM12878 (F) and K562 (G) cells anchored on DHS centers within TFBS-clustered regions in GM12878 but not in K562 in each TFBS-clustered region category. (H-I) Nucleosome occupancy profiles in K562 (H) and GM12878 (I) cells anchored on DHS centers within TFBS-clustered regions in K562 but not in GM12878 in each TFBS-clustered region category. (J-K) DNase I

cleavage profiles in GM12878 (J) and K562 (K) cells anchored on DHS centers within TFBS-clustered regions in GM12878 but not in K562 in each TFBS-clustered region category. The average DNase I cleavage at these regions is higher in GM12878 than in K562. (L-M) DNase I cleavage profiles in K562 (L) and GM12878 (M) cells anchored on DHS centers within TFBS-clustered regions in K562 but not in GM12878 in each TFBS-clustered region category. The average DNase I cleavage at these regions is higher in K562 than in GM12878. (N-O) Fast Fourier transform (FFT) spectra corresponding to the nucleosome occupancy profiles in GM12878 (N) and K562 (O) cells at the period of positioning across GM12878 specific TFBS-clustered regions in each TFBS-clustered region category. (P-Q) Fast Fourier transform (FFT) spectra corresponding to the nucleosome occupancy profiles in K562 (P) and GM12878 (Q) at the period of positioning across K562 specific TFBS-clustered regions in each TFBS-clustered region category. (R-S) Nucleosome depletion "D" corresponding to the nucleosome occupancy profiles in GM12878 (R) and K562 (S) at the period of positioning across GM12878 specific TFBS-clustered regions in each TFBS-clustered region category. (T-U) Nucleosome depletion "D" corresponding to the nucleosome occupancy profiles in K562 (R) and GM12878 (S) at the period of positioning across K562 specific TFBS-clustered regions in each TFBS-clustered region category.

Figure S6. Lineage programming of the TFBS-clustered regions, related to Figure 6

(A) Distributions of cell type number in which a given TFBS-clustered region is observed for: (left) all cell types (n = 47); (middle-left) paraxial mesoderm cell types (n = 15); (middle-right) lymphoid cell types (n = 14); and (right) endothelial cell types (n = 10). Width of each shape at a given y value shows the relative frequency of TFBS-clustered regions present in that number of cell types. (B) Landscape elevation related to Figure 6A. (C) PCoA of cell-type relationships for each of the 8 additional cell types and the 47 original cell types. Cell-type coloring is indicated above. Each of the 8 additional cell types are labeled with arrows. (D) 100% and 95% confidence intervals and mean of all 1022 Bk plots. (E-M) 100% and 95% confidence intervals and mean of Bk plots for subsets that omit one to nine TFBS-clustered region categories.

Fig. S1A

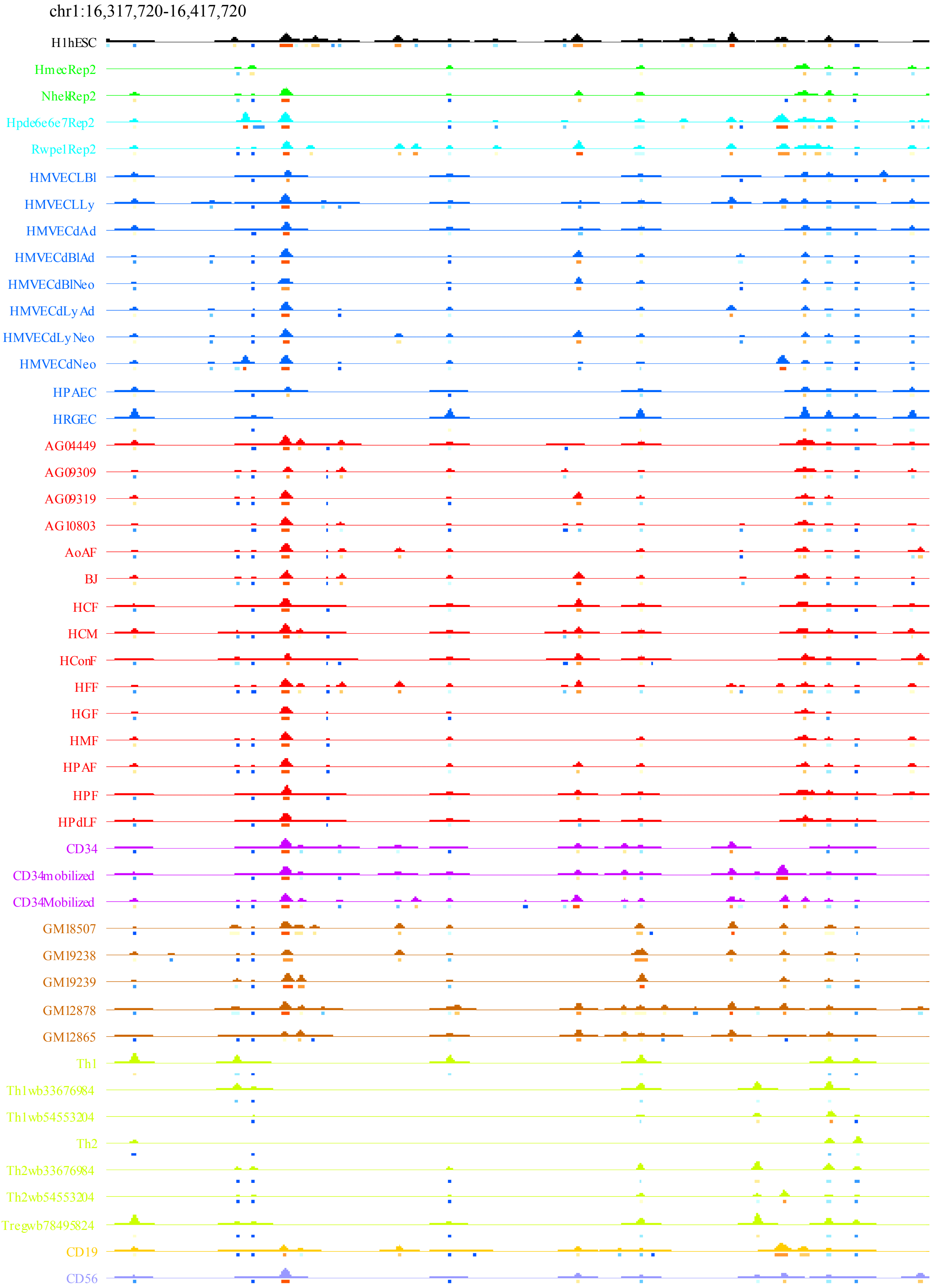
chr1:16,317,720-16,417,720
H1hESC
HmecRep2
NhekRep2
Hpde6e6e7Rep2
RwpelRep2
HMVECLBl
HMVECLLy
HMVECdAd
HMVECdBlAd
HMVECdBlNeo
HMVECdLyAd
HMVECdLyNeo
HMVECdNeo
HPAEC
HRGEC
AG04449
AG09309
AG09319
AG10803
AoAF
BJ
HCF
HCM
HConF
HFF
HGF
HMF
HPAF
HPF
HPdLF
CD34
CD34mobilized
CD34Mobilized
GM18507
GM19238
GM19239
GM12878
GM12865
Th1
Th1wb33676984
Th1wb54553204
Th2
Th2wb33676984
Th2wb54553204
Tregwb78495824
CD19
CD56

Fig. S1B

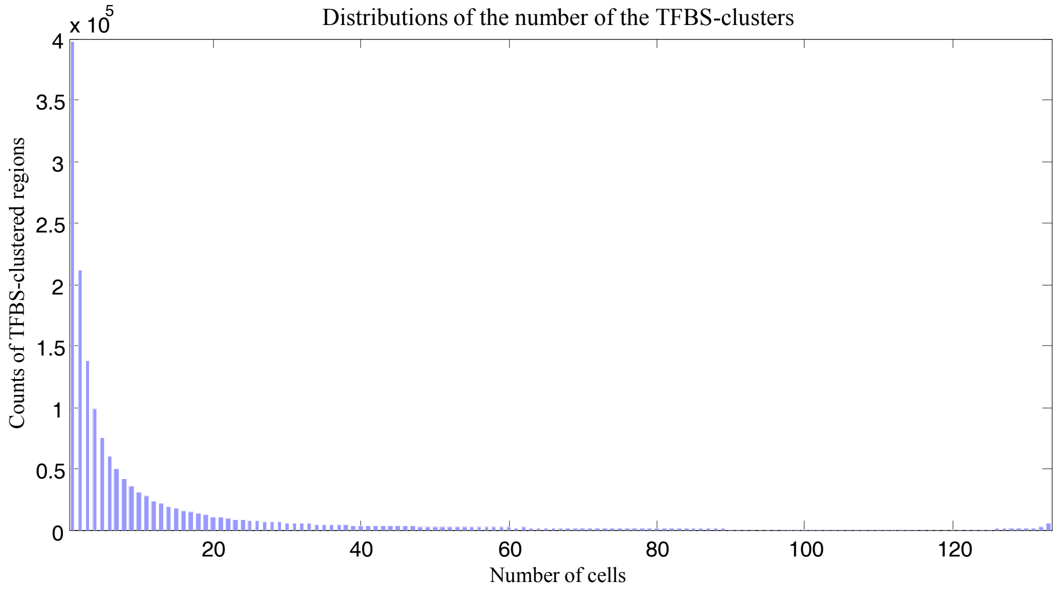

Fig. S1C

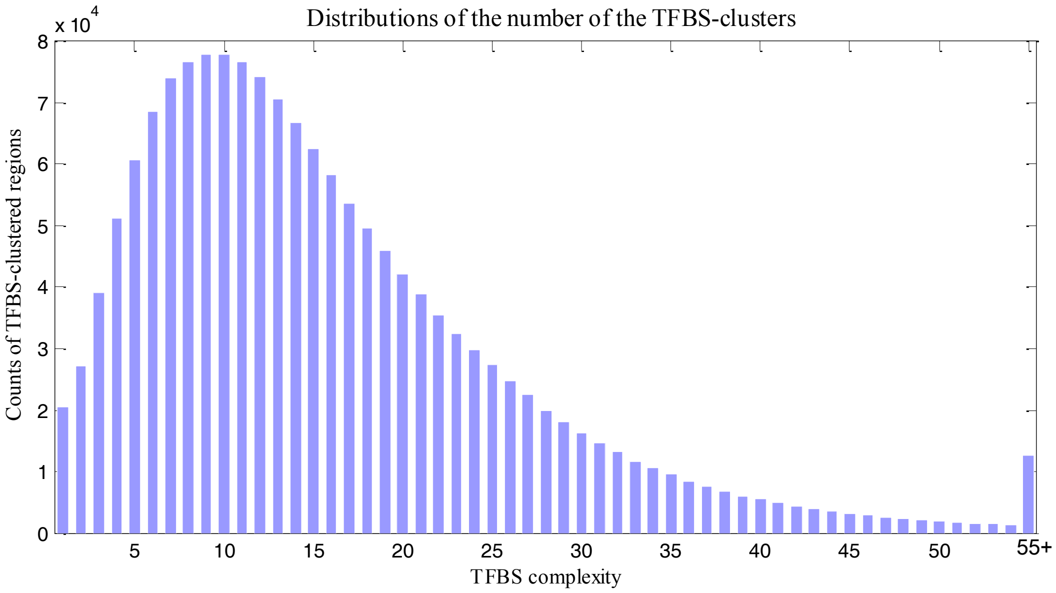

Fig. S1D

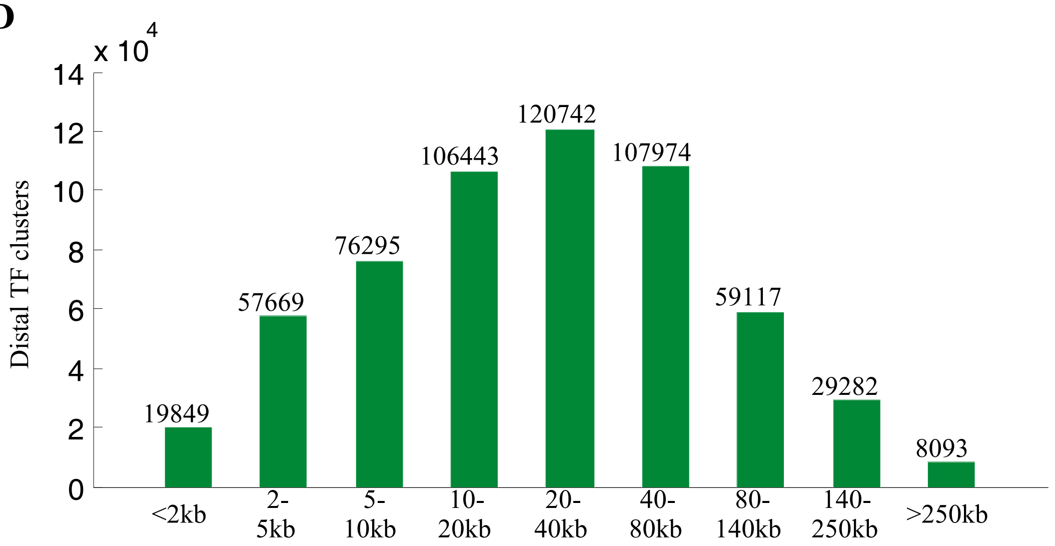

Fig. S2A

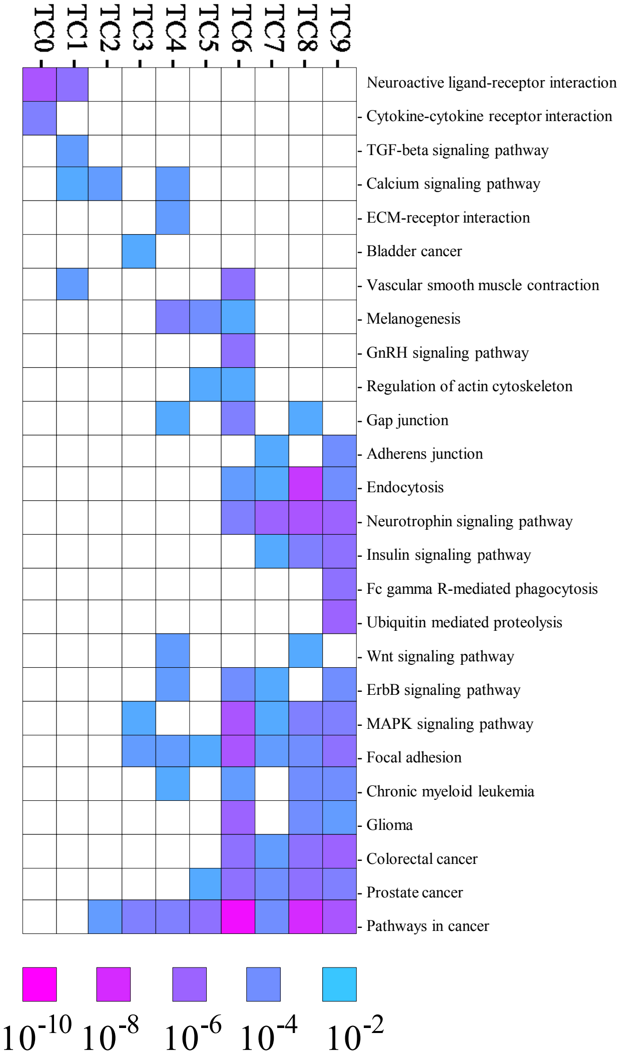
TC0
TC1
TC2
TC3
TC4
TC5
TC6
TC7
TC8
TC9
Neuroactive ligand-receptor interaction
Cytokine-cytokine receptor interaction
TGF-beta signaling pathway
Calcium signaling pathway
ECM-receptor interaction
Bladder cancer
Vascular smooth muscle contraction
Melanogenesis
GnRH signaling pathway
Regulation of actin cytoskeleton
Gap junction
Adherens junction
Endocytosis
Neurotrophin signaling pathway
Insulin signaling pathway
Fc gamma R-mediated phagocytosis
Ubiquitin mediated proteolysis
Wnt signaling pathway
ErbB signaling pathway
MAPK signaling pathway
Focal adhesion
Chronic myeloid leukemia
Glioma
Colorectal cancer
Prostate cancer
Pathways in cancer
10^{-10}
10^{-8}
10^{-6}
10^{-4}
10^{-2}

Fig. S3A Positive enrichment of TFs:

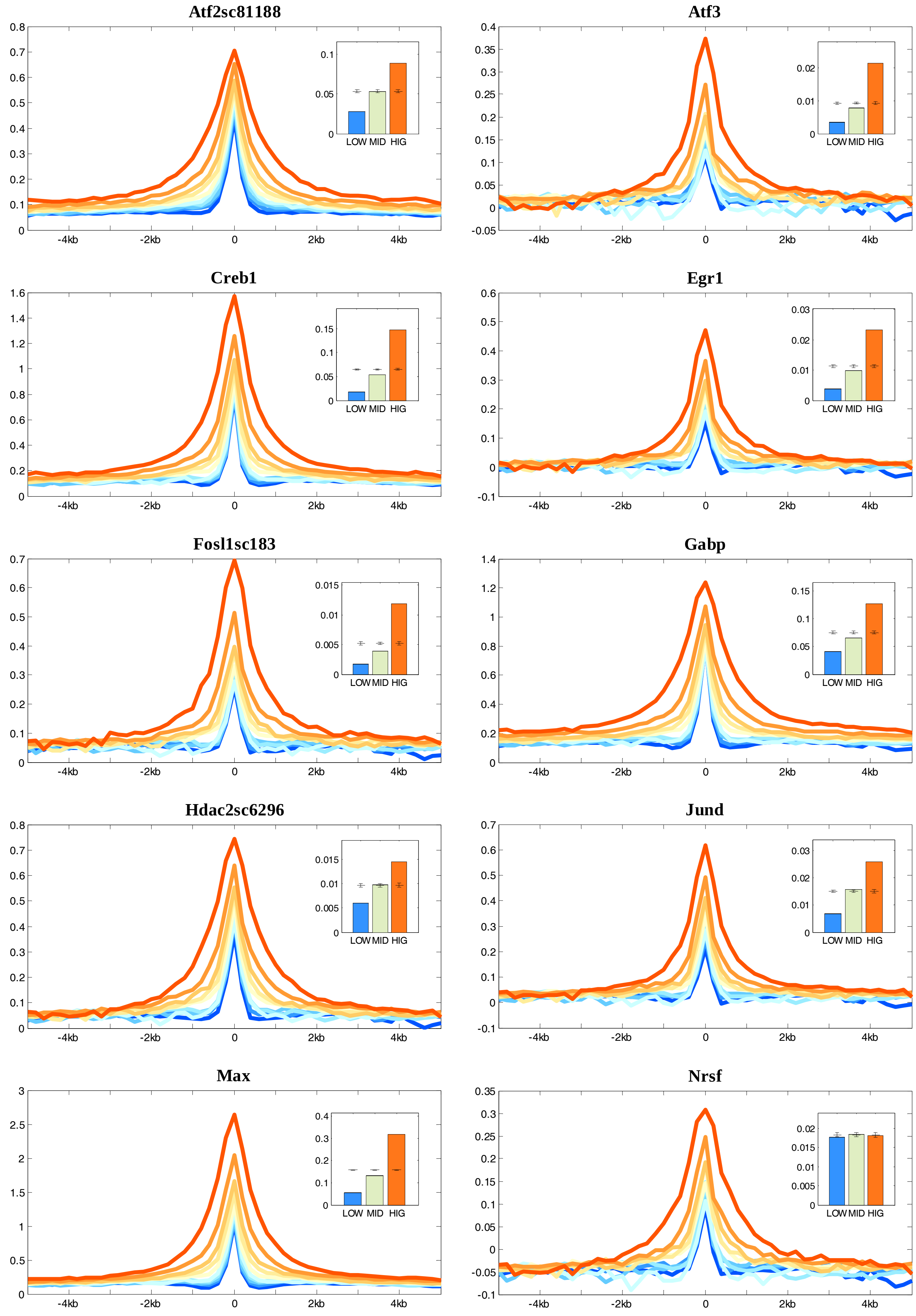

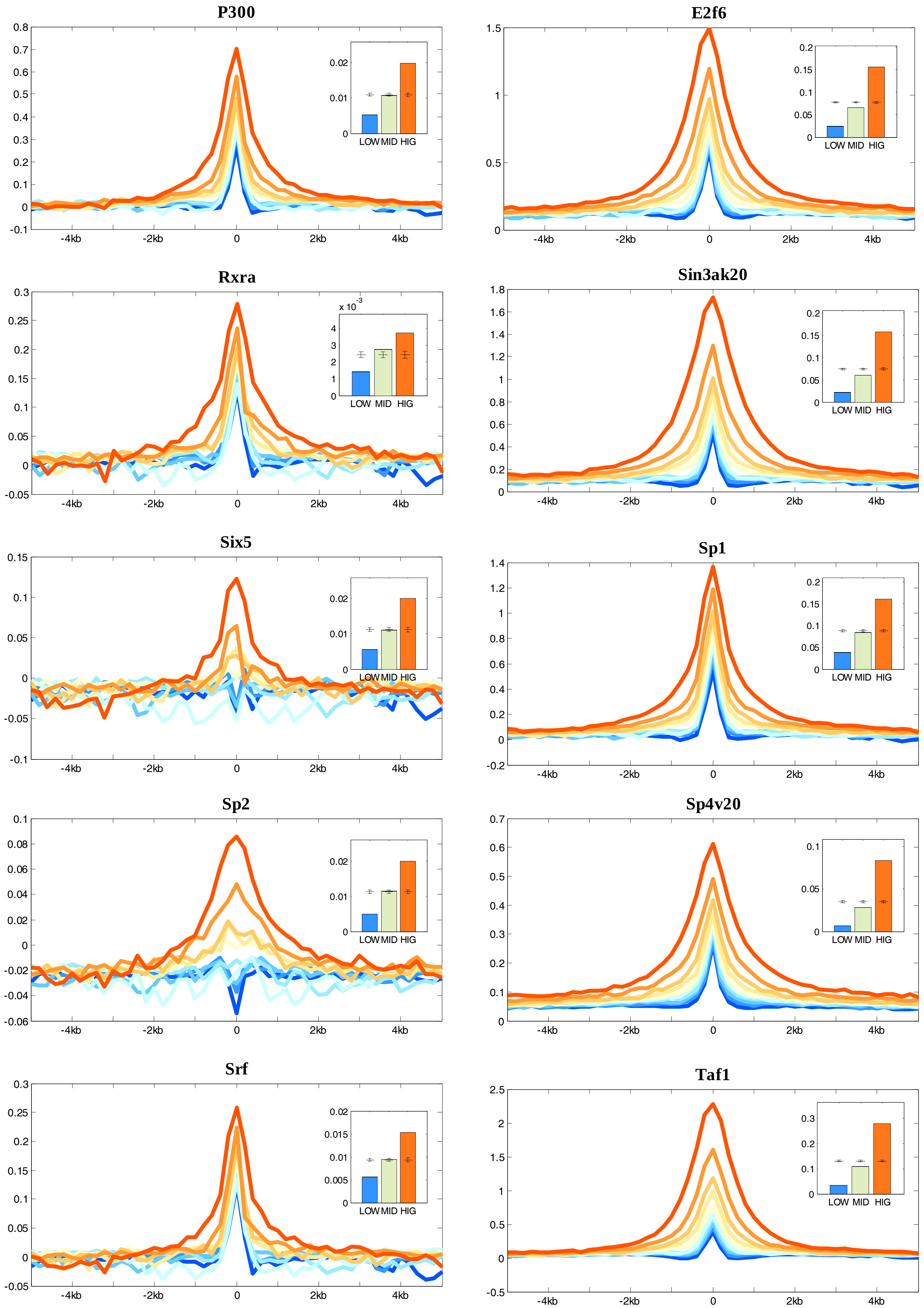
P300
E2f6
Rxra
Sin3ak20
Six5
Sp1
Sp2
Sp4v20
Srf
Taf1
-4kb
-2kb
0
2kb
4kb
LOW MID HIG
x 10^-3

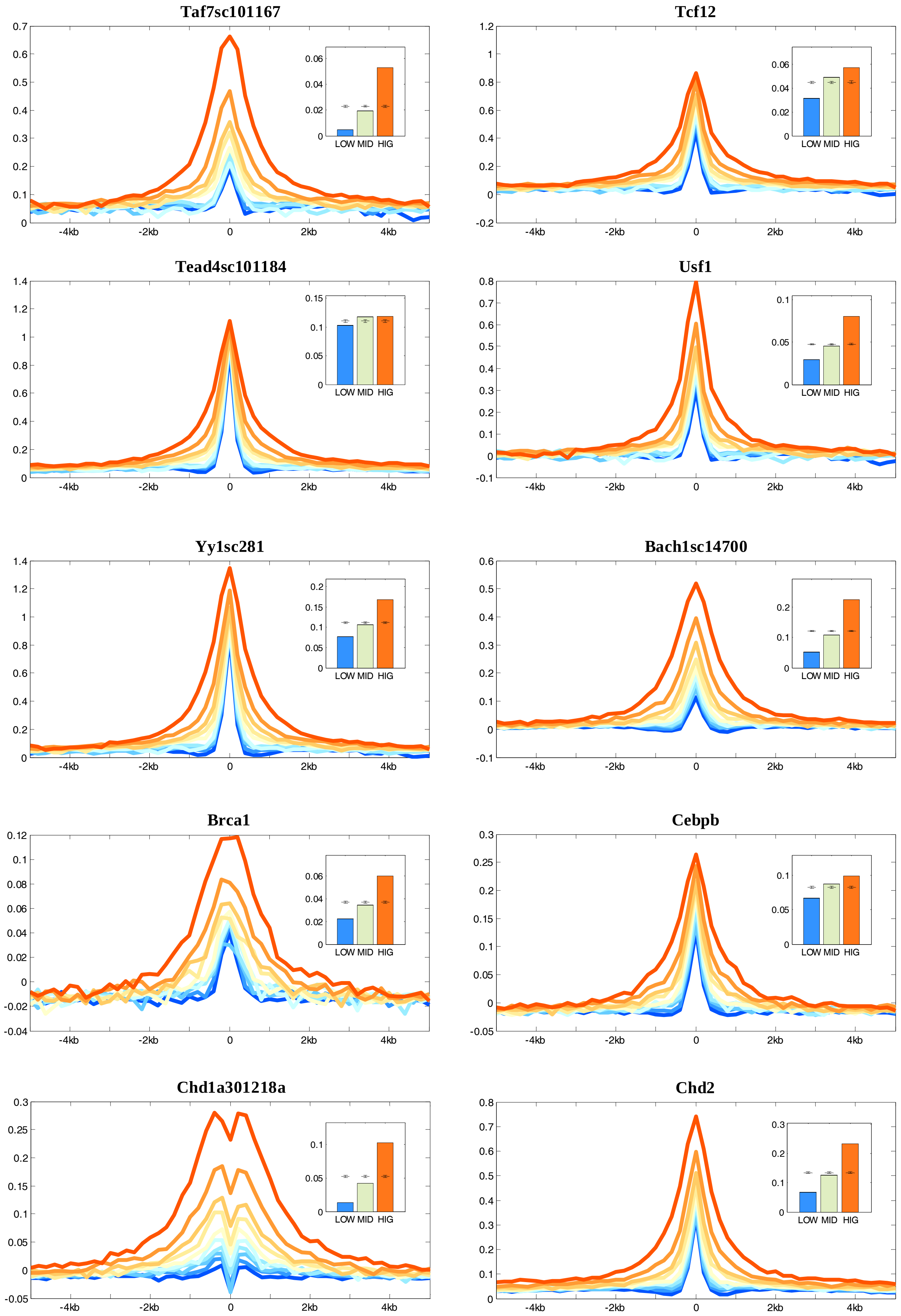
Taf7sc101167
0.7
0.6
0.5
0.4
0.3
0.2
0.1
0
-4kb
-2kb
0
2kb
4kb
0.06
0.04
0.02
0
LOW MID HIG
Tcf12
1.2
1
0.8
0.6
0.4
0.2
0
-0.2
-4kb
-2kb
0
2kb
4kb
0.06
0.04
0.02
0
LOW MID HIG
Tead4sc101184
1.4
1.2
1
0.8
0.6
0.4
0.2
0
-4kb
-2kb
0
2kb
4kb
0.15
0.1
0.05
0
LOW MID HIG
Usf1
0.8
0.7
0.6
0.5
0.4
0.3
0.2
0.1
0
-0.1
-4kb
-2kb
0
2kb
4kb
0.1
0.05
0
LOW MID HIG
Yy1sc281
1.4
1.2
1
0.8
0.6
0.4
0.2
0
-4kb
-2kb
0
2kb
4kb
0.2
0.15
0.1
0.05
0
LOW MID HIG
Bach1sc14700
0.6
0.5
0.4
0.3
0.2
0.1
0
-0.1
-4kb
-2kb
0
2kb
4kb
0.2
0.1
0
LOW MID HIG
Brca1
0.12
0.1
0.08
0.06
0.04
0.02
0
-0.02
-0.04
-4kb
-2kb
0
2kb
4kb
0.06
0.04
0.02
0
LOW MID HIG
Cebpb
0.3
0.25
0.2
0.15
0.1
0.05
0
-0.05
-4kb
-2kb
0
2kb
4kb
0.1
0.05
0
LOW MID HIG
Chd1a301218a
0.3
0.25
0.2
0.15
0.1
0.05
0
-0.05
-4kb
-2kb
0
2kb
4kb
0.1
0.05
0
LOW MID HIG
Chd2
0.8
0.7
0.6
0.5
0.4
0.3
0.2
0.1
0
-4kb
-2kb
0
2kb
4kb
0.3
0.2
0.1
0
LOW MID HIG

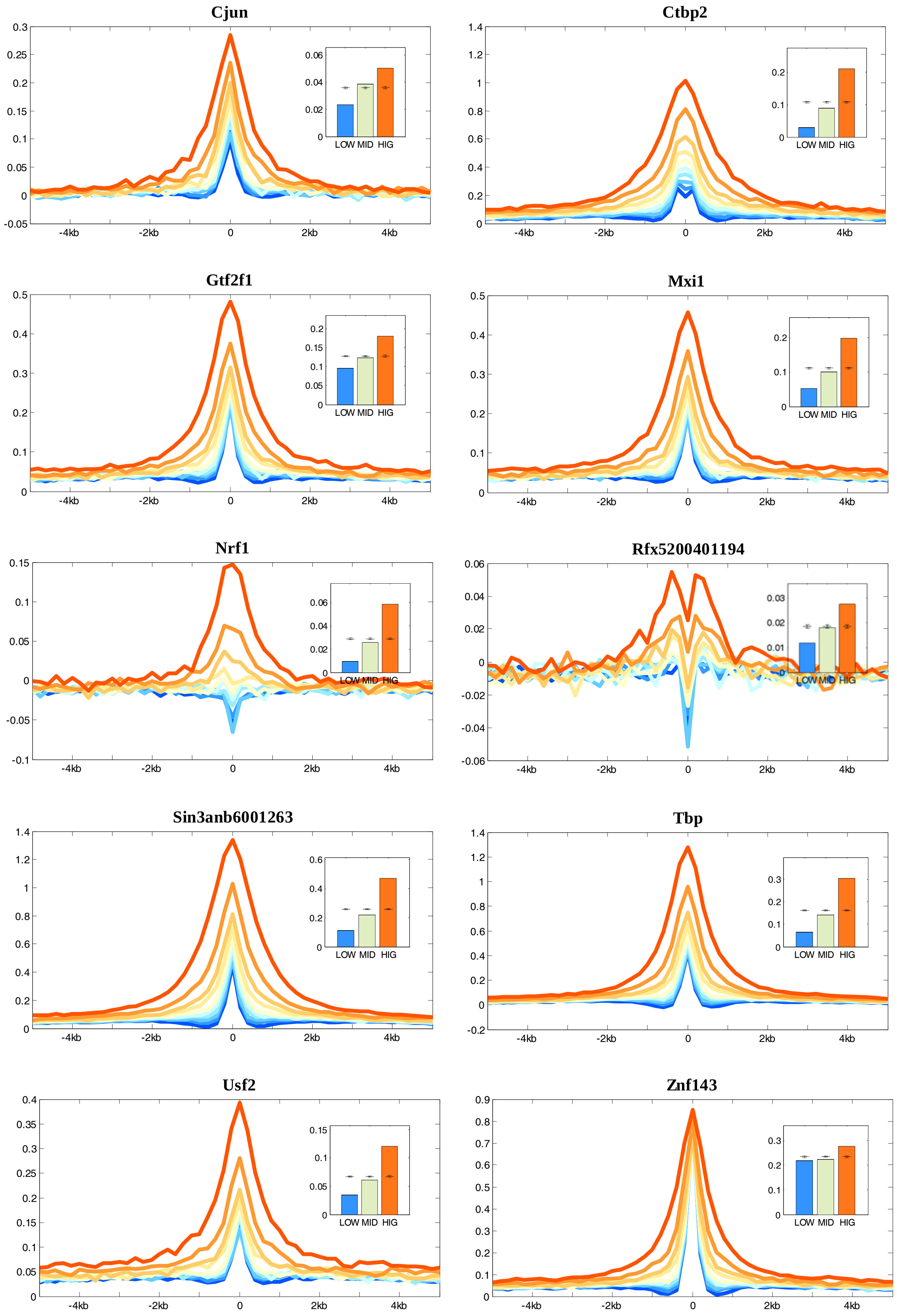

Cjun
Ctbp2
Gtf2f1
Mxi1
Nrf1
Rfx5200401194
Sin3anb6001263
Tbp
Usf2
Znf143
-4kb
-2kb
0
2kb
4kb
LOW MID HIG

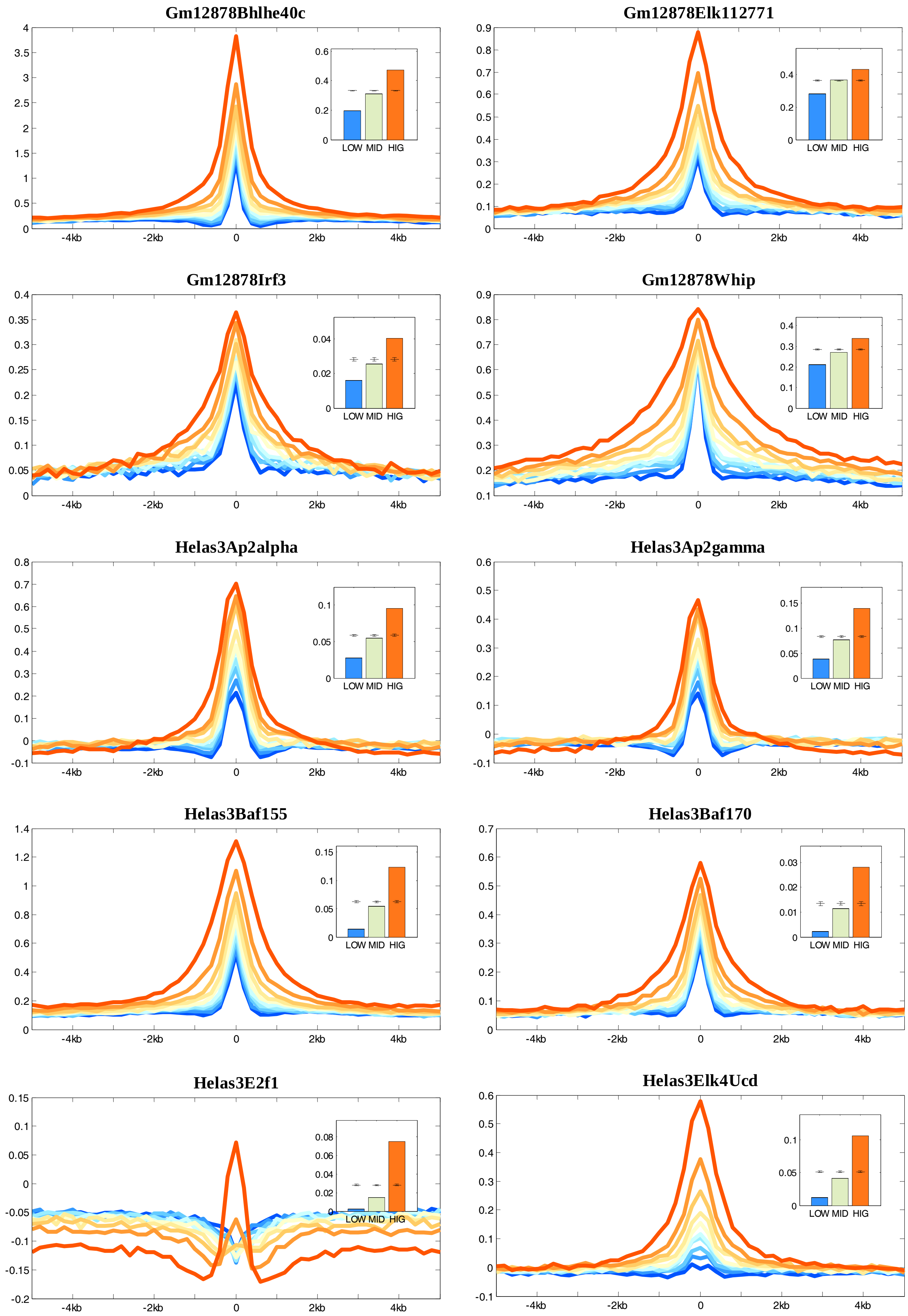
Gm12878Bhlhe40c
Gm12878Elk112771
Gm12878Irf3
Gm12878Whip
Helas3Ap2alpha
Helas3Ap2gamma
Helas3Baf155
Helas3Baf170
Helas3E2f1
Helas3Elk4Ucd
LOW MID HIG
-4kb -2kb 0 2kb 4kb

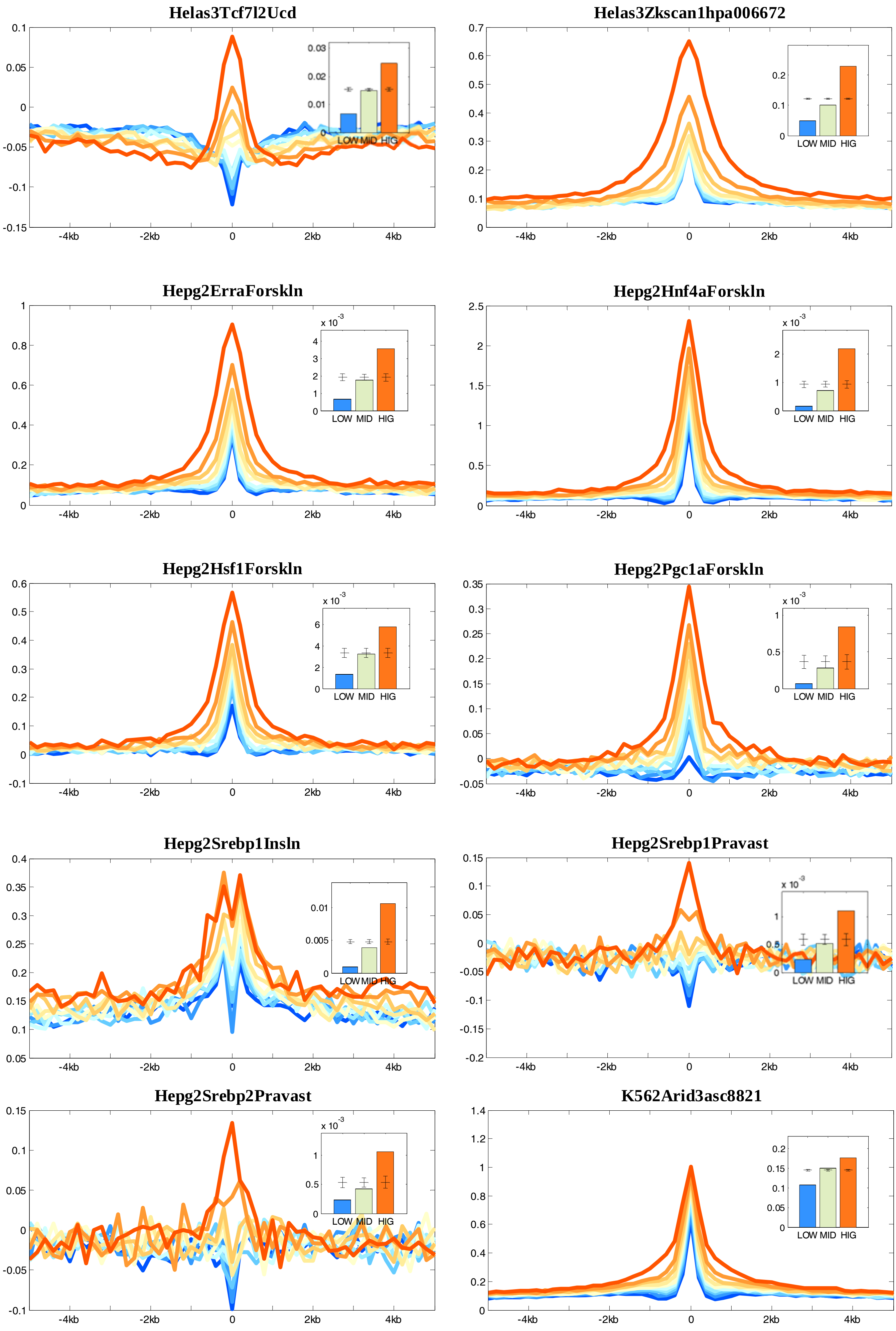
Helas3Tcf7l2Ucd
0.1
0.05
0
-0.05
-0.1
-0.15
0.03
0.02
0.01
0
LOW MID HIG
-4kb
-2kb
0
2kb
4kb
Helas3Zkscan1hpa006672
0.7
0.6
0.5
0.4
0.3
0.2
0.1
0
0.2
0.1
0
LOW MID HIG
-4kb
-2kb
0
2kb
4kb
Hepg2ErraForskln
1
0.8
0.6
0.4
0.2
0
x 10^-3
4
3
2
1
0
LOW MID HIG
-4kb
-2kb
0
2kb
4kb
Hepg2Hnf4aForskln
2.5
2
1.5
1
0.5
0
x 10^-3
2
1
0
LOW MID HIG
-4kb
-2kb
0
2kb
4kb
Hepg2Hsf1Forskln
0.6
0.5
0.4
0.3
0.2
0.1
0
-0.1
x 10^-3
6
4
2
0
LOW MID HIG
-4kb
-2kb
0
2kb
4kb
Hepg2Pgc1aForskln
0.35
0.3
0.25
0.2
0.15
0.1
0.05
0
-0.05
x 10^-3
1
0.5
0
LOW MID HIG
-4kb
-2kb
0
2kb
4kb
Hepg2Srebp1Insln
0.4
0.35
0.3
0.25
0.2
0.15
0.1
0.05
0.01
0.005
0
LOW MID HIG
-4kb
-2kb
0
2kb
4kb
Hepg2Srebp1Pravast
0.15
0.1
0.05
0
-0.05
-0.1
-0.15
-0.2
x 10^-3
1
0.5
0
LOW MID HIG
-4kb
-2kb
0
2kb
4kb
Hepg2Srebp2Pravast
0.15
0.1
0.05
0
-0.05
-0.1
x 10^-3
1
0.5
0
LOW MID HIG
-4kb
-2kb
0
2kb
4kb
K562Arid3asc8821
1.4
1.2
1
0.8
0.6
0.4
0.2
0
0.2
0.15
0.1
0.05
0
LOW MID HIG
-4kb
-2kb
0
2kb
4kb

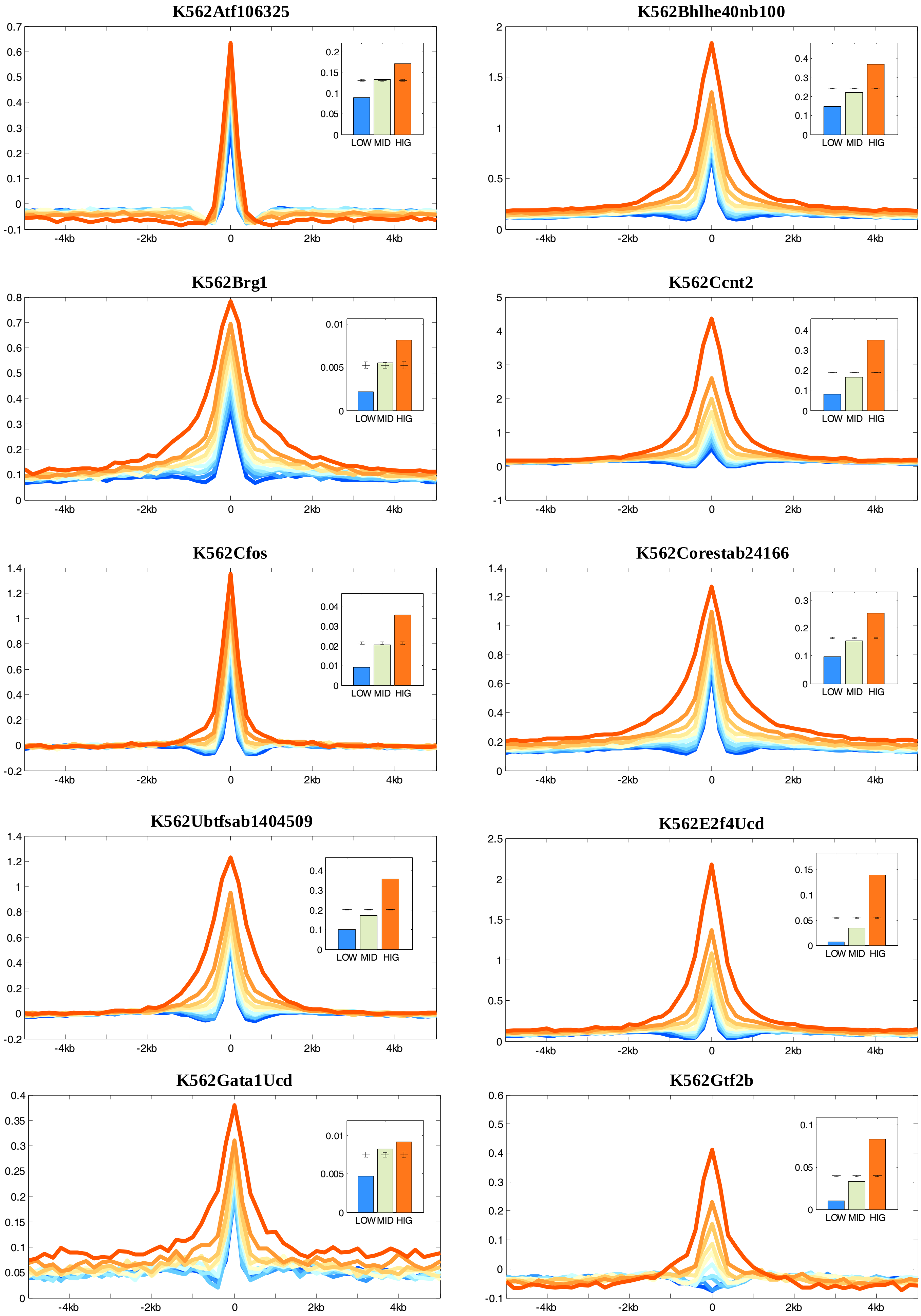
K562Atf106325
K562Bhlhe40nb100
K562Brg1
K562Ccnt2
K562Cfos
K562Corestab24166
K562Ubtfsab1404509
K562E2f4Ucd
K562Gata1Ucd
K562Gtf2b
LOW MID HIG
-4kb -2kb 0 2kb 4kb

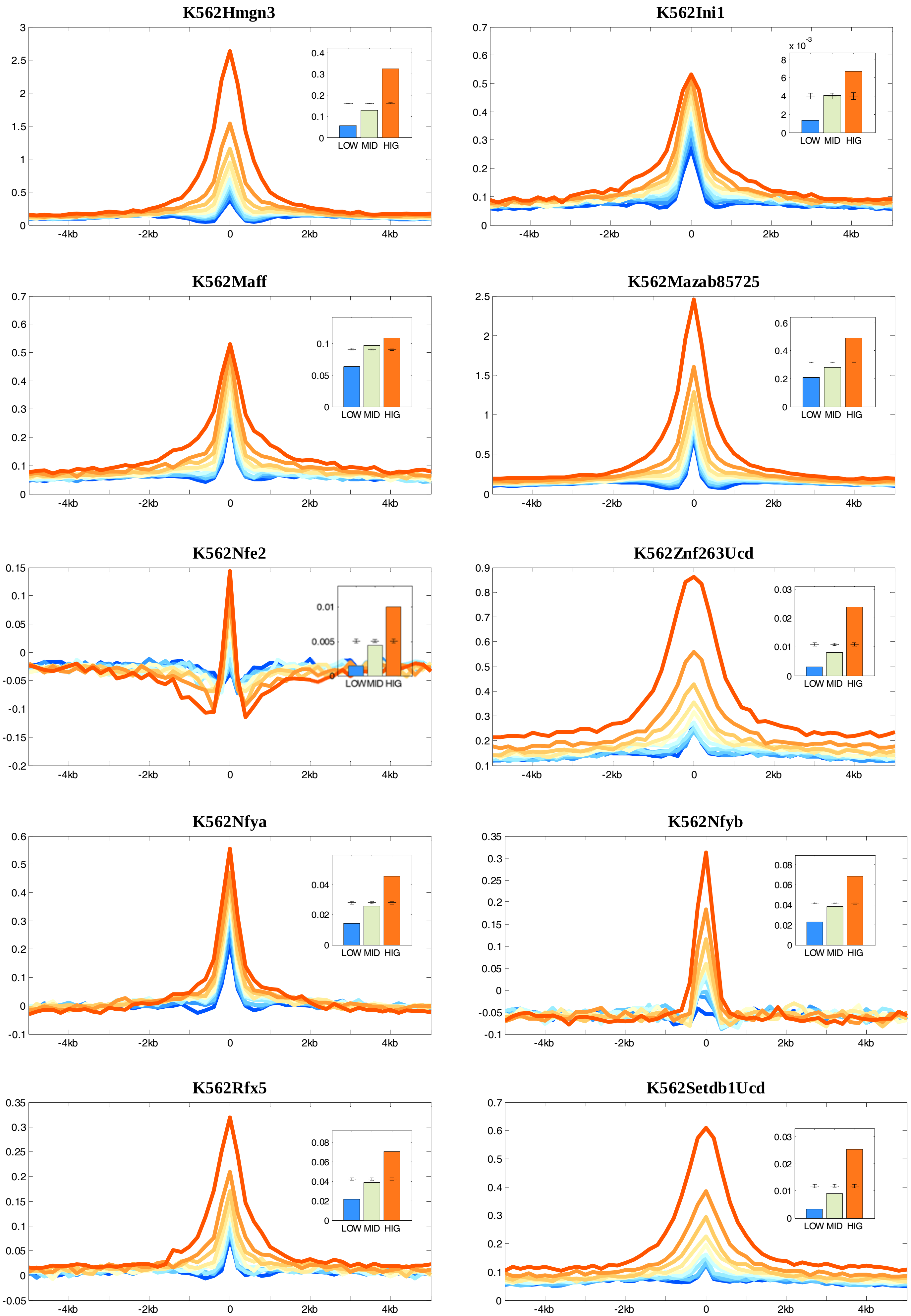
K562Hmgn3
LOW MID HIG
-4kb -2kb 0 2kb 4kb
K562Ini1
x 10^-3
LOW MID HIG
-4kb -2kb 0 2kb 4kb
K562Maff
LOW MID HIG
-4kb -2kb 0 2kb 4kb
K562Mazab85725
LOW MID HIG
-4kb -2kb 0 2kb 4kb
K562Nfe2
LOWMID HIG
-4kb -2kb 0 2kb 4kb
K562Znf263Ucd
LOW MID HIG
-4kb -2kb 0 2kb 4kb
K562Nfya
LOW MID HIG
-4kb -2kb 0 2kb 4kb
K562Nfyb
LOW MID HIG
-4kb -2kb 0 2kb 4kb
K562Rfx5
LOW MID HIG
-4kb -2kb 0 2kb 4kb
K562Setdb1Ucd
LOW MID HIG
-4kb -2kb 0 2kb 4kb

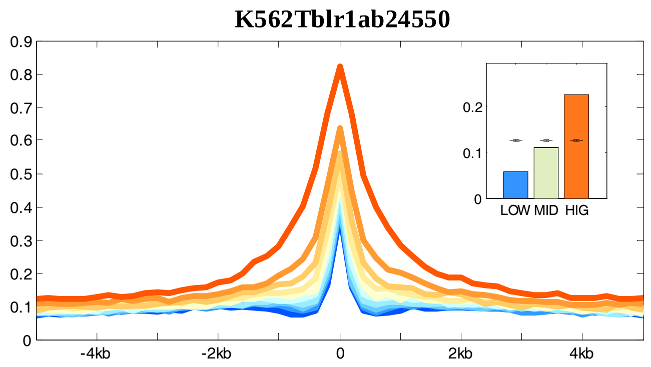
K562Tblr1ab24550
0.9
0.8
0.7
0.6
0.5
0.4
0.3
0.2
0.1
0
-4kb
-2kb
0
2kb
4kb
0.2
0.1
0
LOW MID HIG

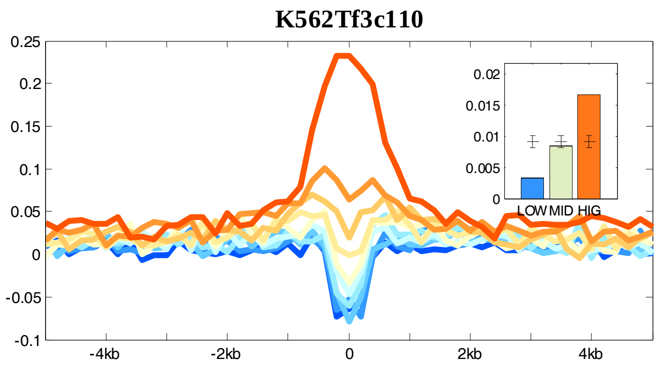
K562Tf3c110
0.25
0.2
0.15
0.1
0.05
0
-0.05
-0.1
-4kb
-2kb
0
2kb
4kb
0.02
0.015
0.01
0.005
0
LOW MID HIG

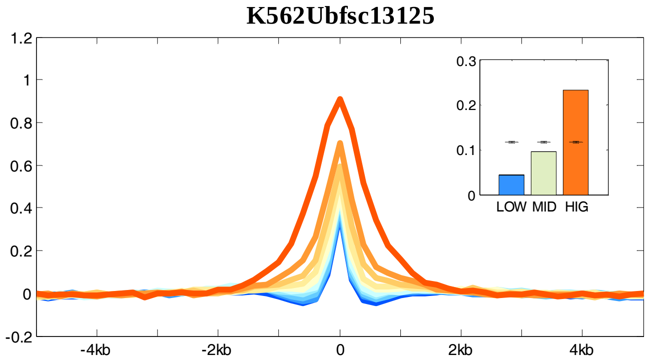
K562Ubfsc13125
1.2
1
0.8
0.6
0.4
0.2
0
-0.2
-4kb
-2kb
0
2kb
4kb
0.3
0.2
0.1
0
LOW MID HIG

Fig. S3B Negative enrichment of TFs

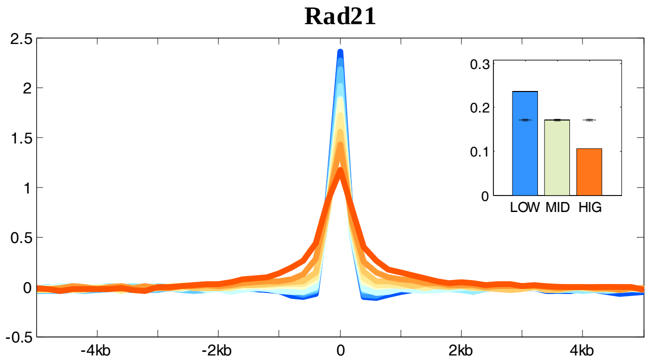

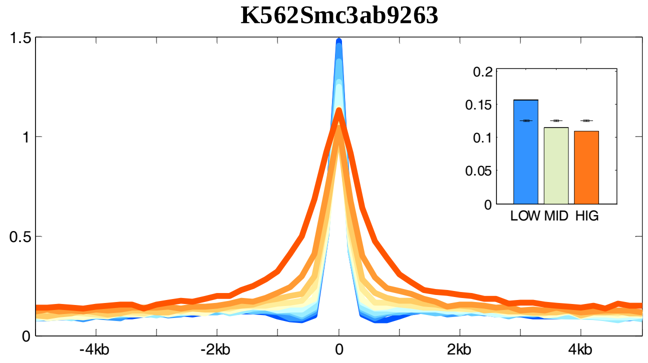

Fig. S3C Positive enrichment of TFs within median-TFBS-complexity TFBS-clustered regions

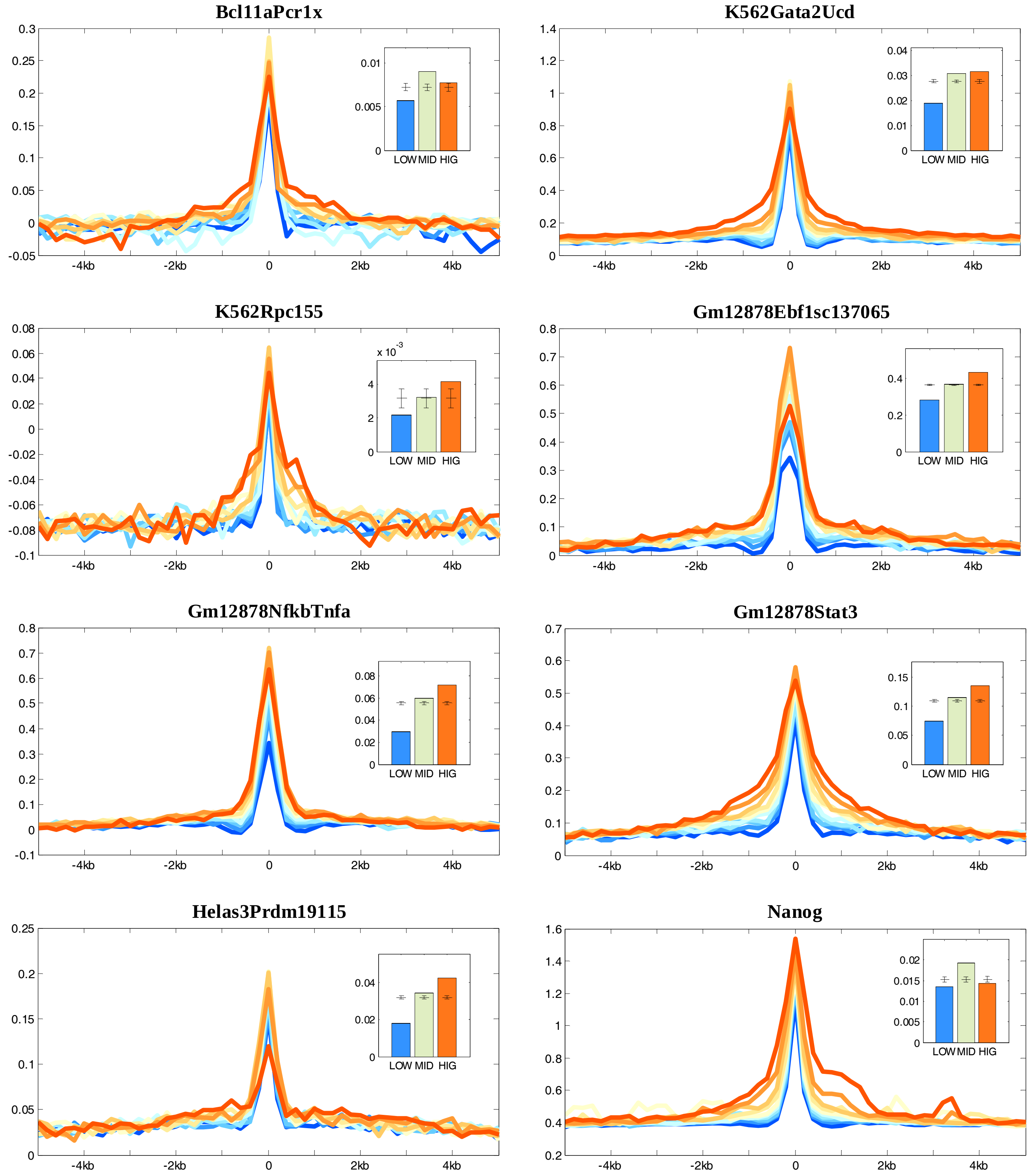

Fig. S3D Positive depletion of TFs

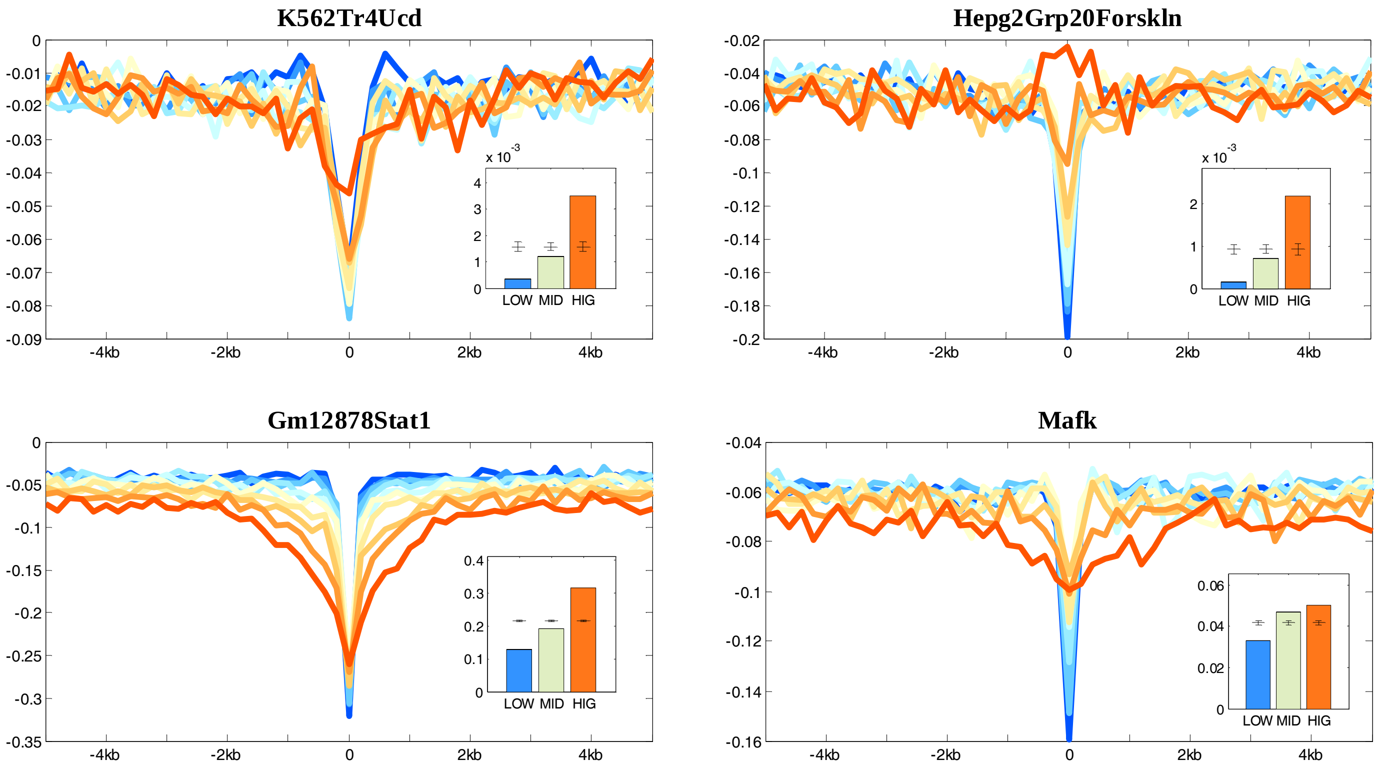

Fig. S3E Negative depletion of TFs

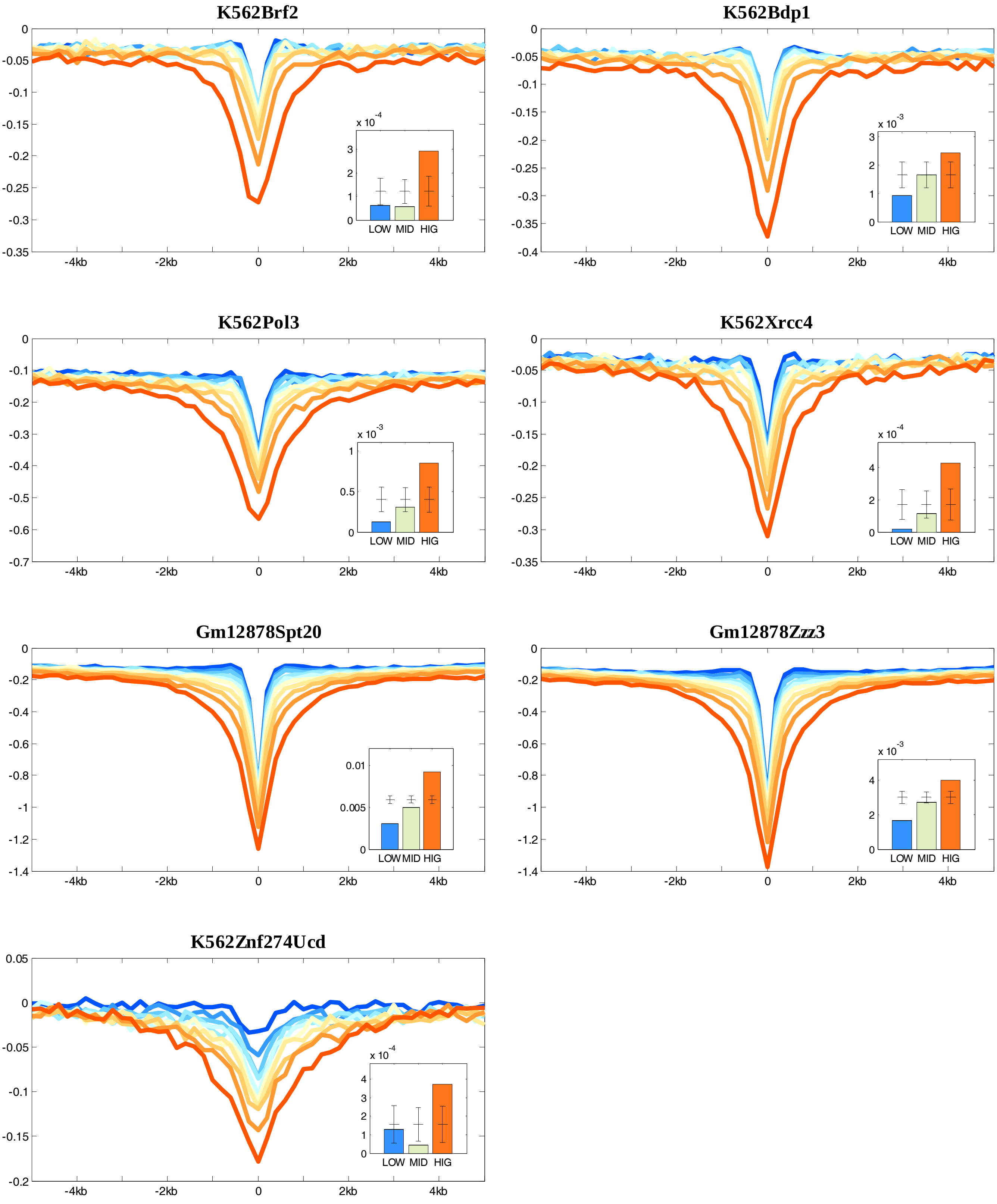

Fig. S3F RNA seq

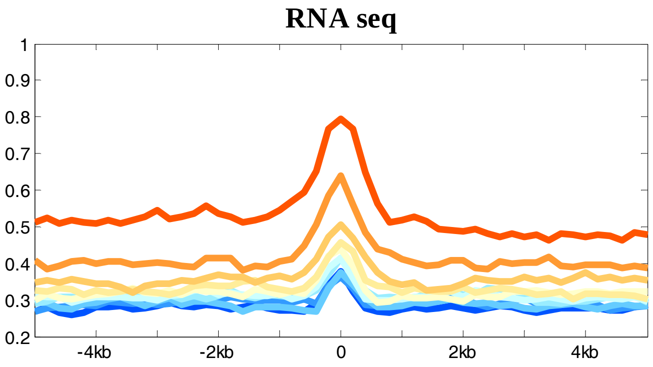

Fig. S4A

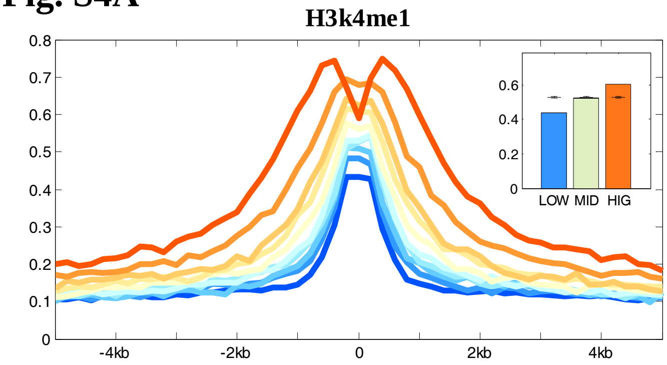

Fig. S4B

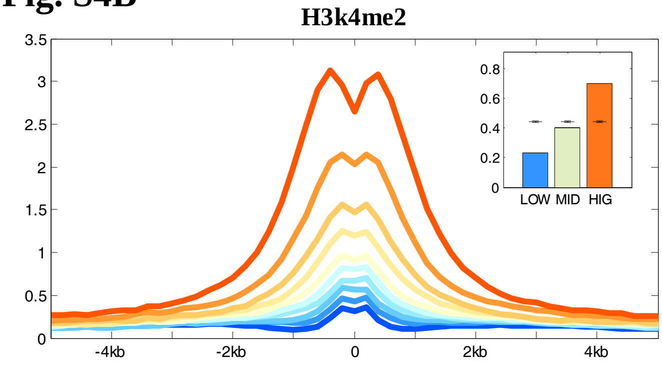

Fig. S4C

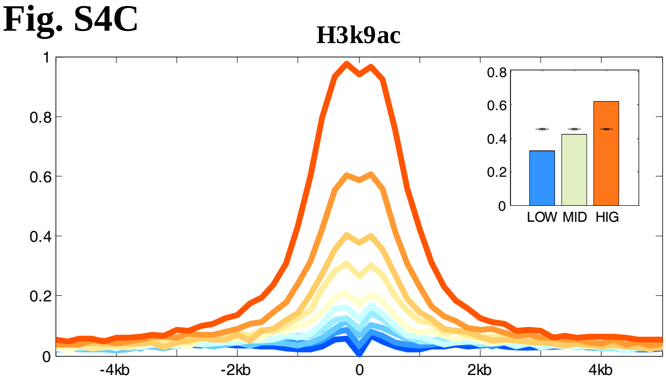

Fig. S4D

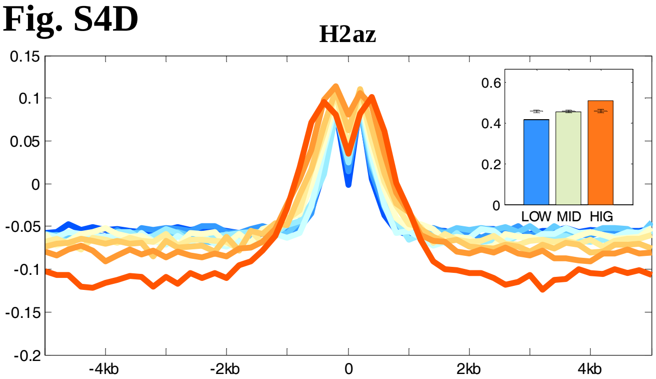

Fig. S4E

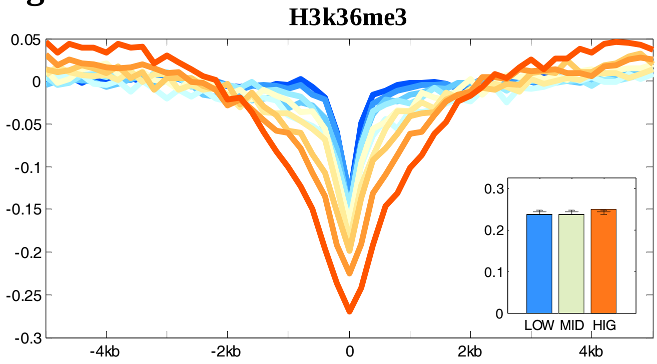

Fig. S4F

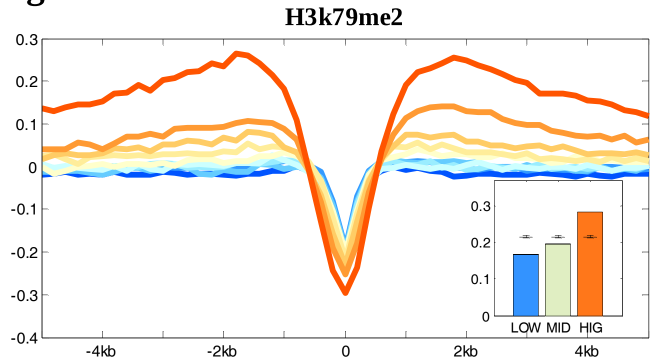

Fig. S4G

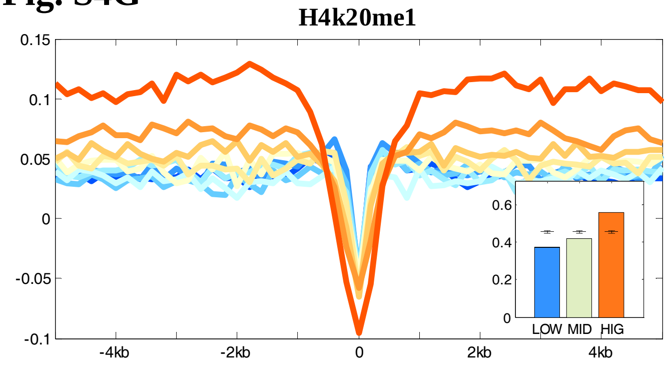

Fig. S4H

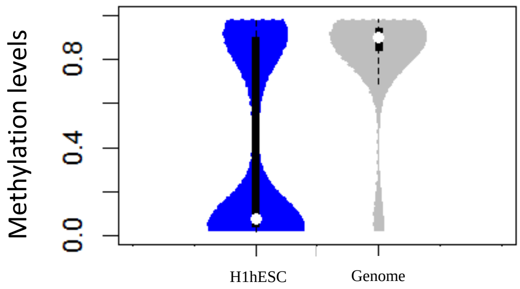

Fig. S4I

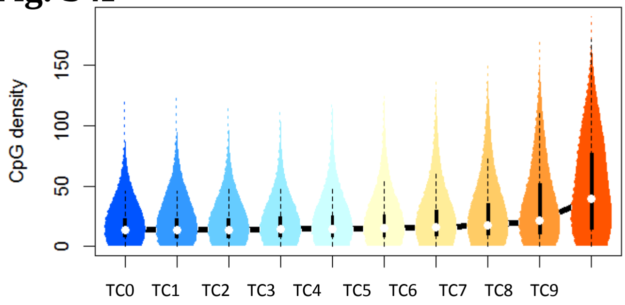

Fig. S4J

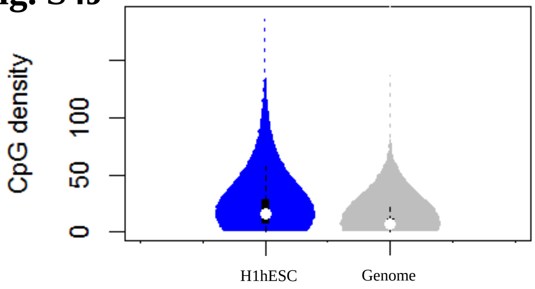

Fig.S5A

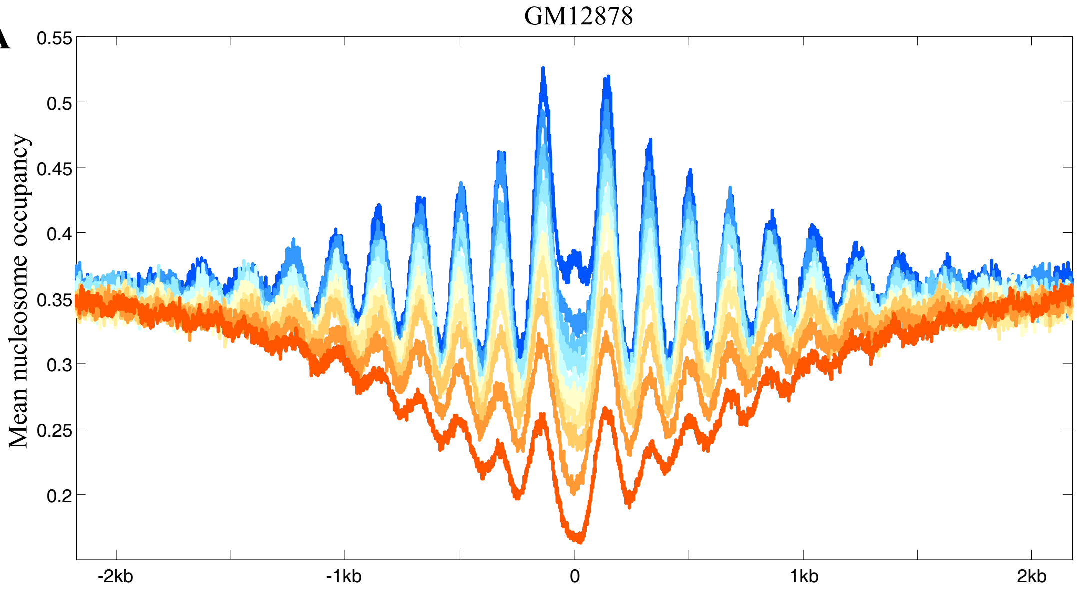

Fig.S5B

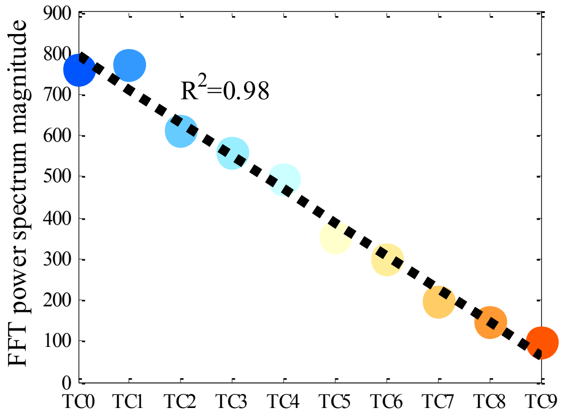

Fig.S5C

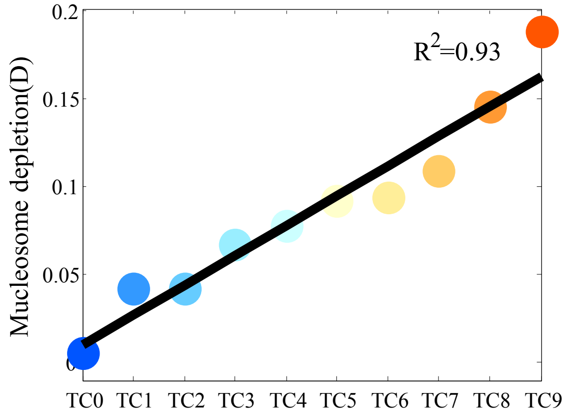

Fig.S5D

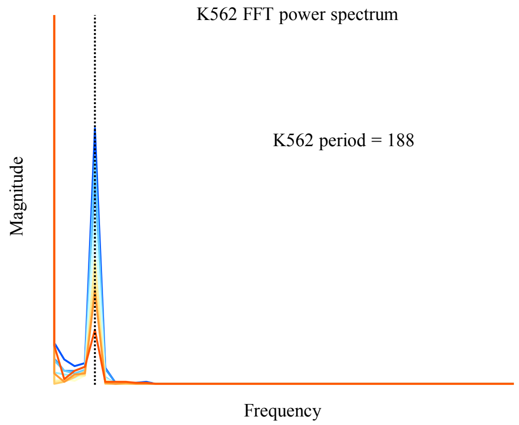

Fig.S5E

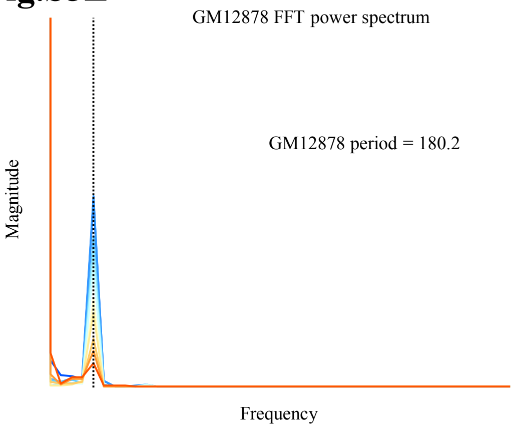

Fig.S5F

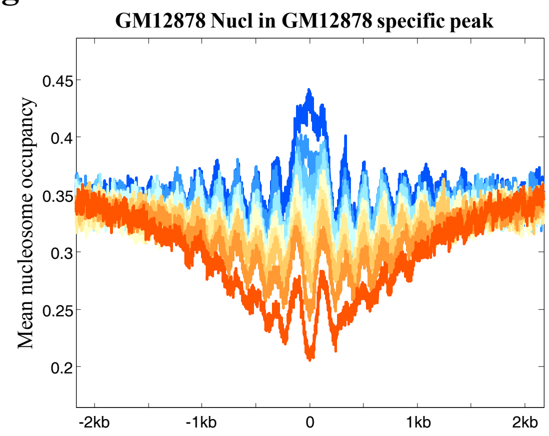

Fig.S5G

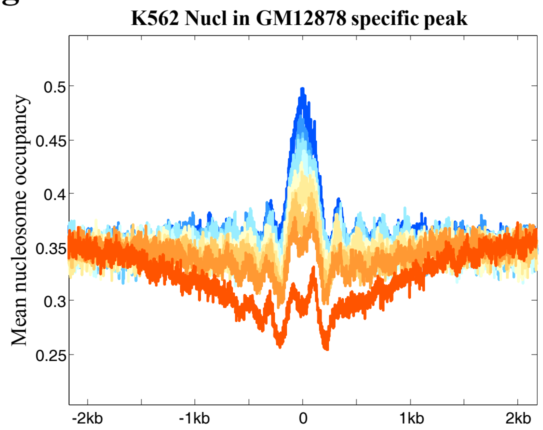

Fig.S5H

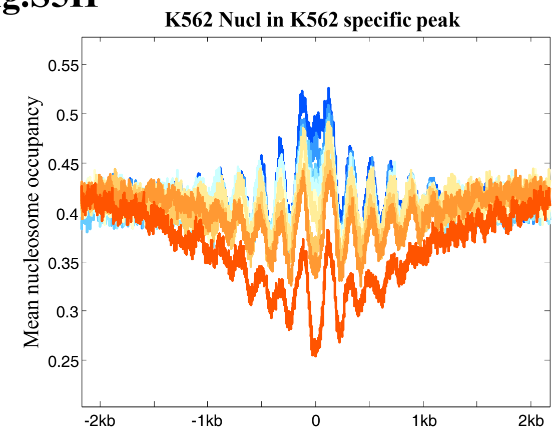

Fig.S5I

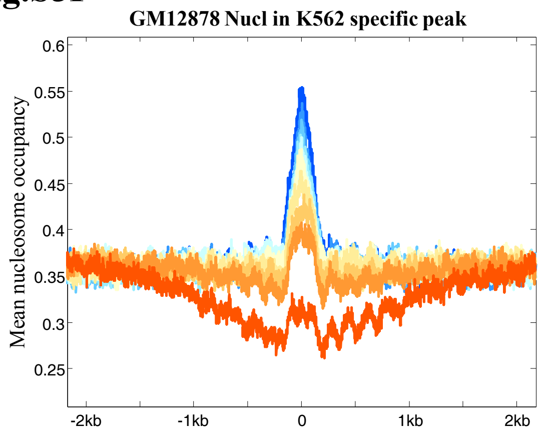

Fig.S5J

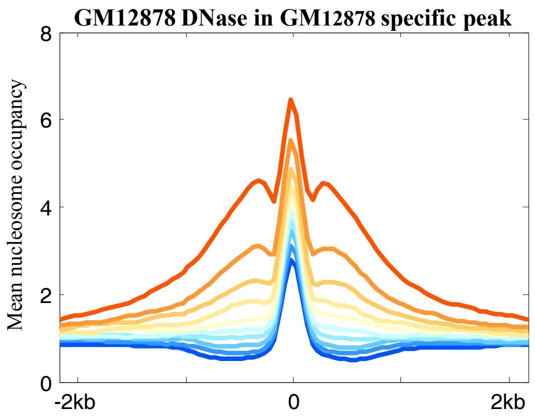

Fig.S5K

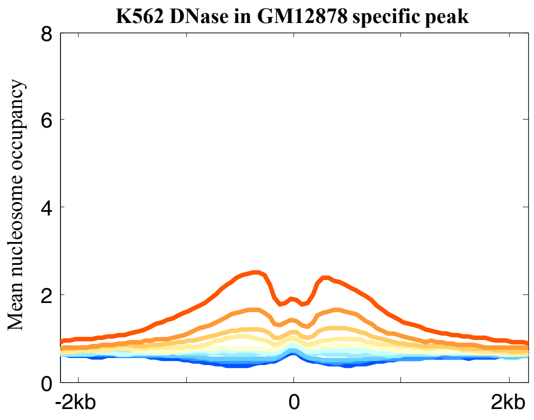

Fig.S5L

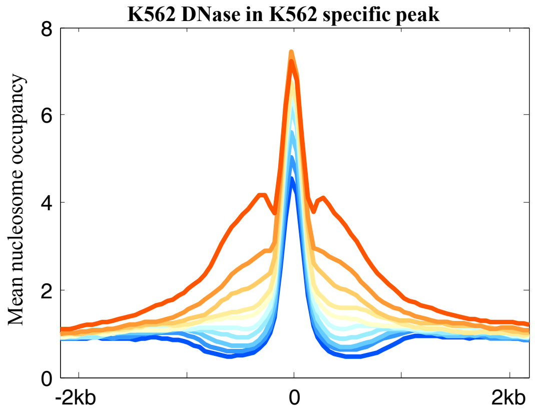

Fig.S5M

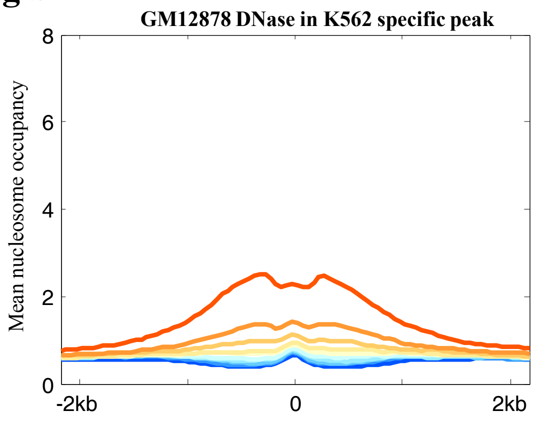

Fig.S5N

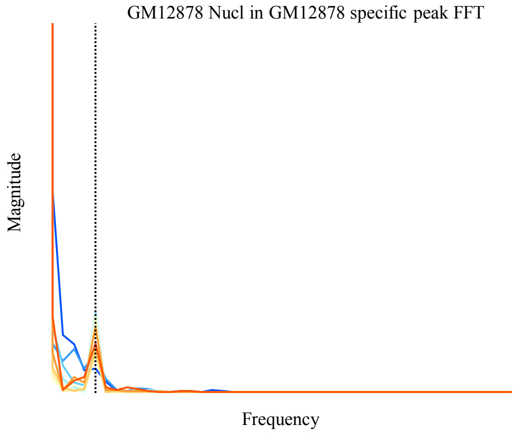

Fig.S5O

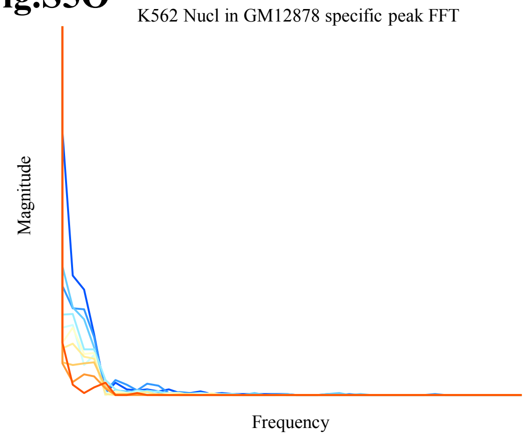

Fig.S5P

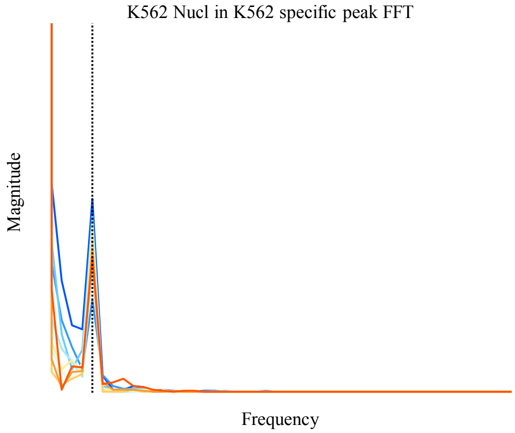

Fig.S5Q

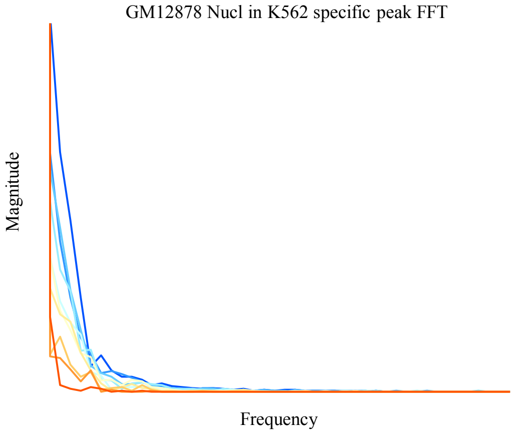

Fig.S5R

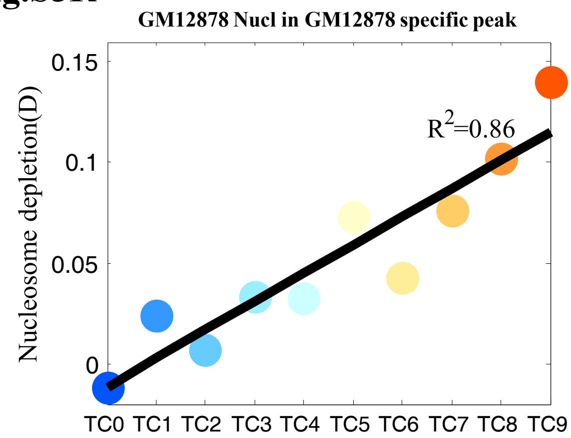

Fig.S5S

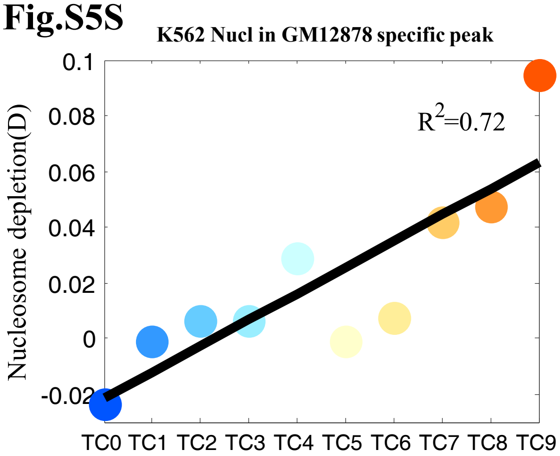

Fig.S5T

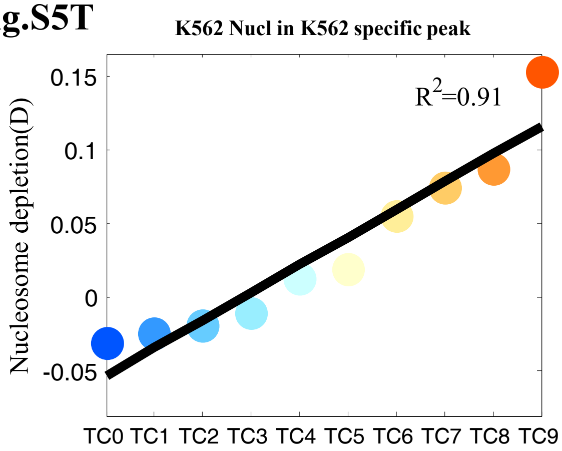

Fig.S5U

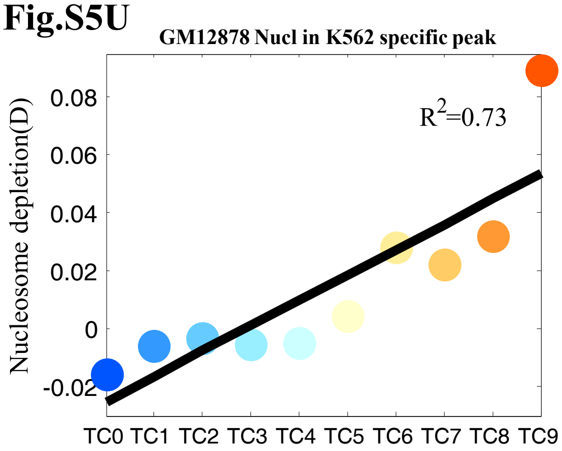

Fig.S6A

Distribution of cells containg a TFBS-clustered region found in **ALL 47 Cell Type**

Distribution of cells containg a TFBS-clustered region found in **Paraxial Mesoderm**

Distribution of cells containg a TFBS-clustered region found in **Lymphoid Cells**

Distribution of cells containg a TFBS-clustered region found in **Endothelial Cells**

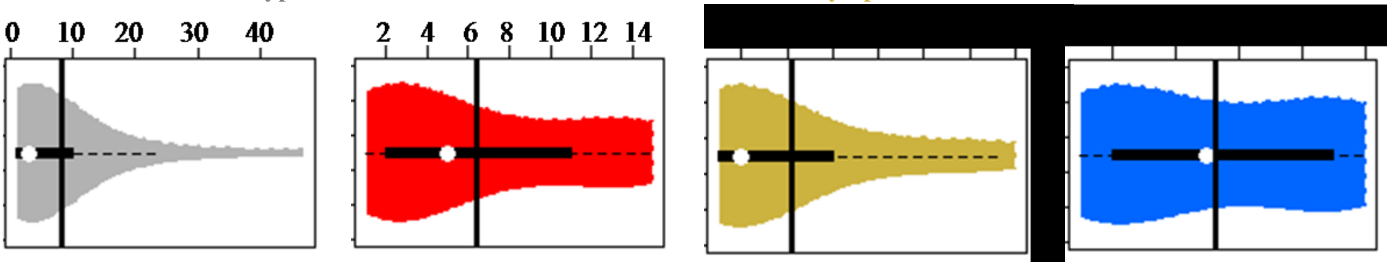

Fig.S6B

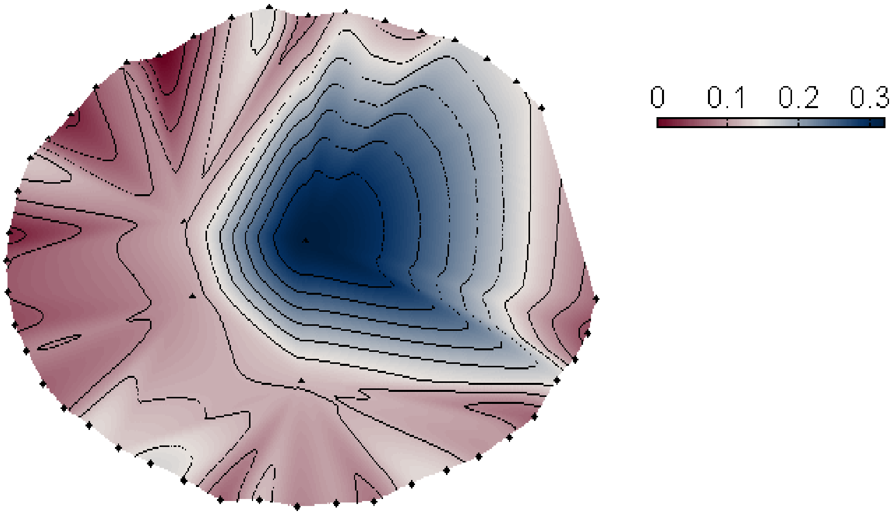

Landscape elevation ralate to Figure 6A

Fig.S6C

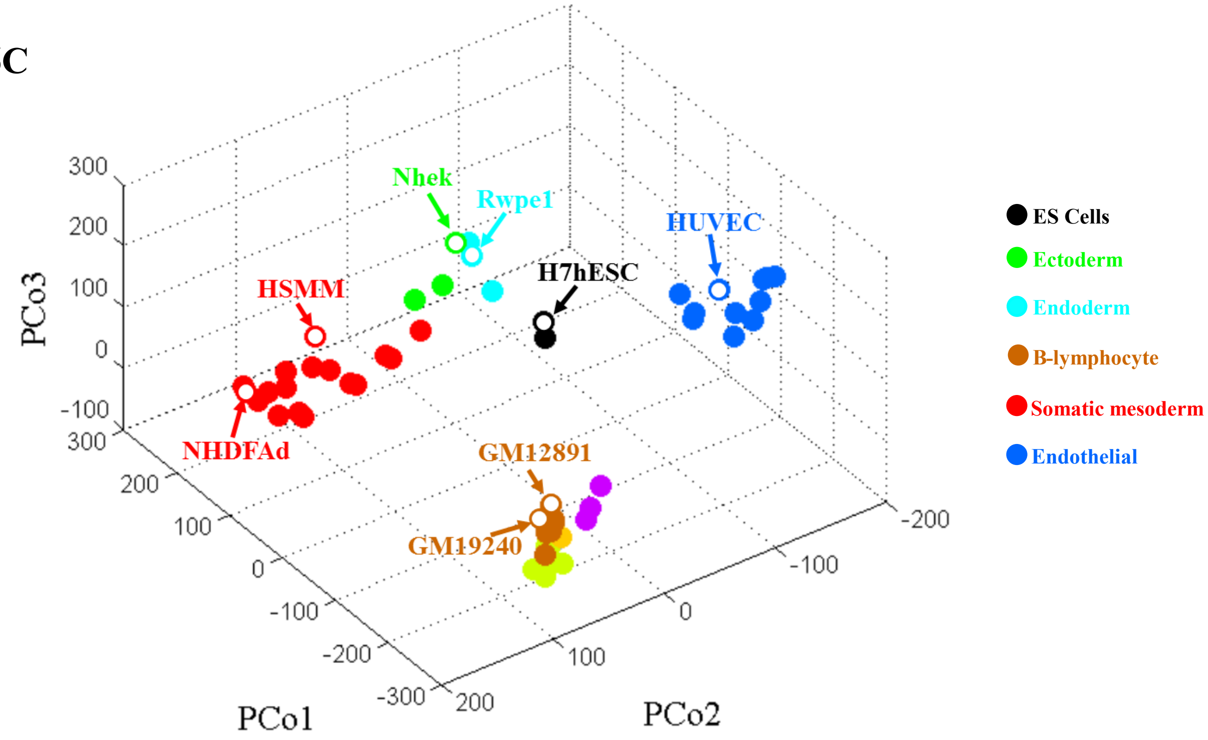

Fig.S6D

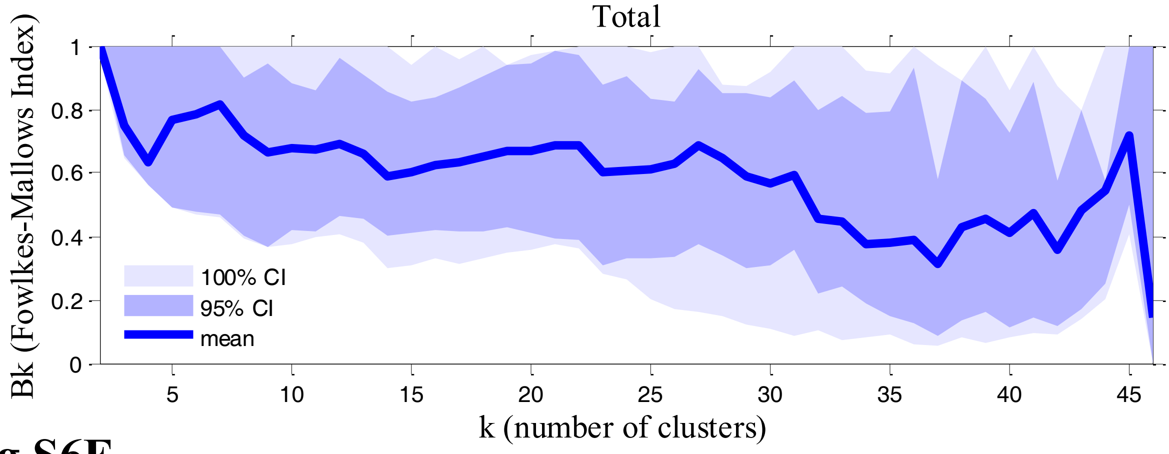

Fig.S6E

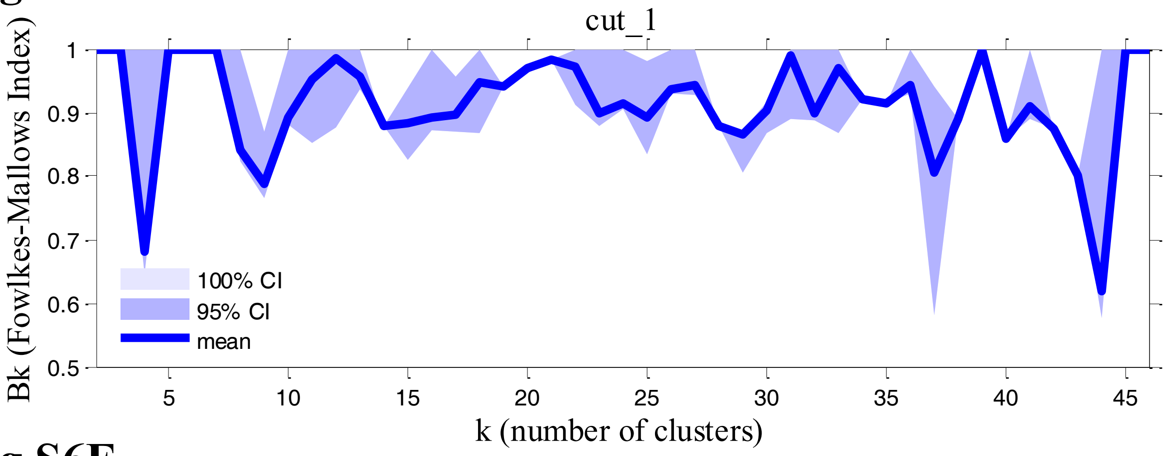

Fig.S6F

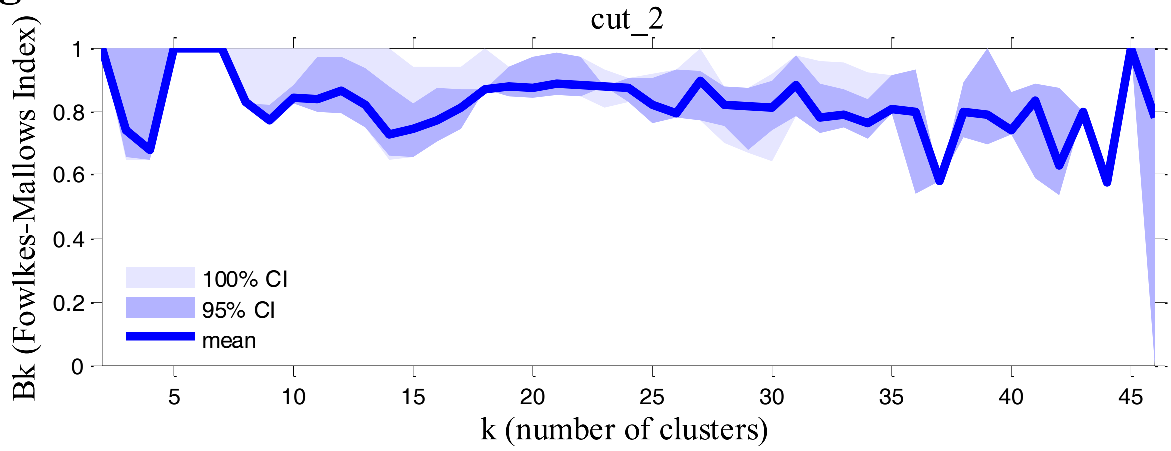

Fig.S6G

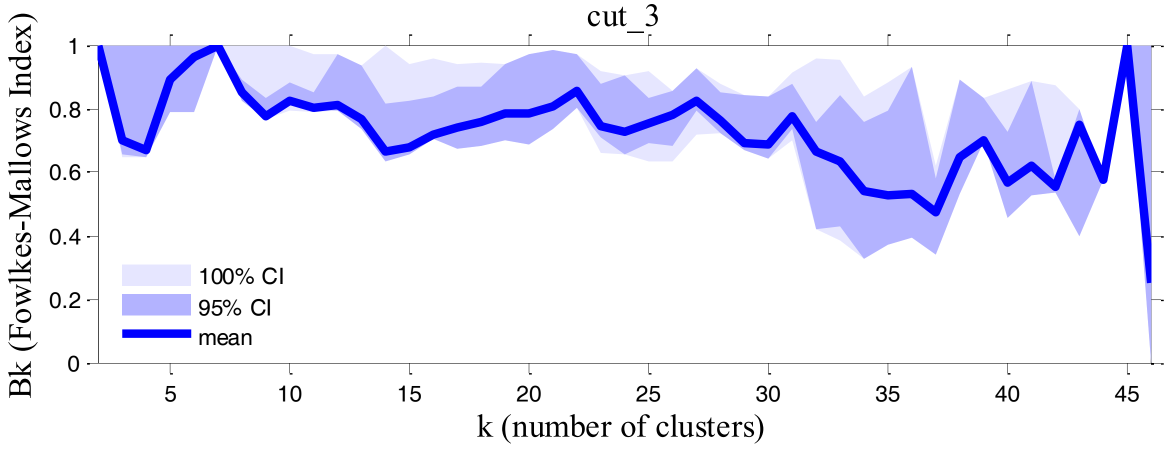

Fig.S6H

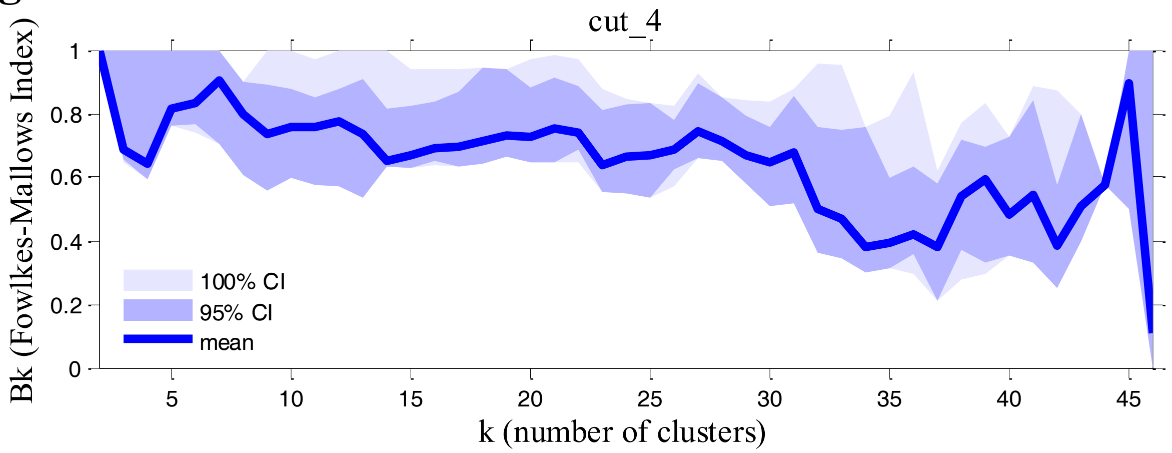

Fig.S6I

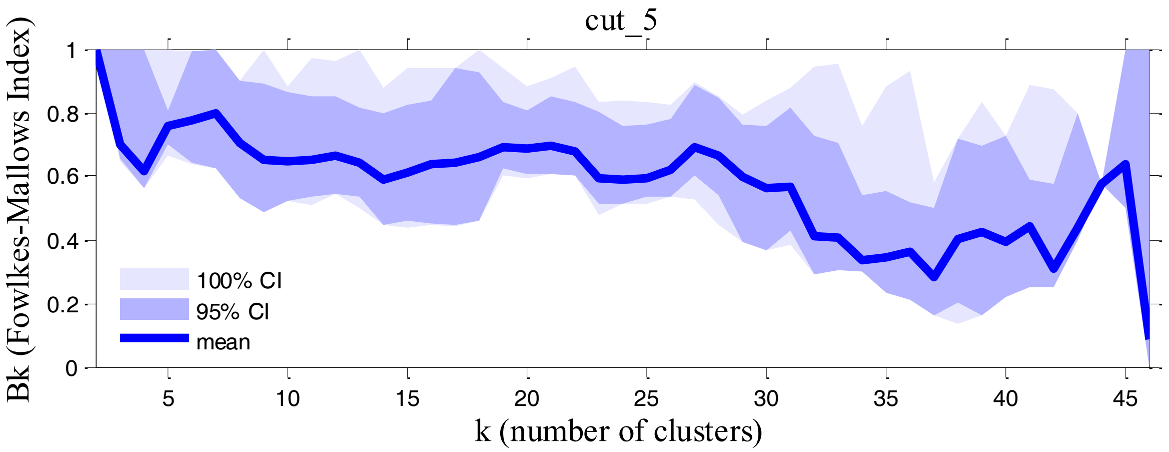

Fig.S6J

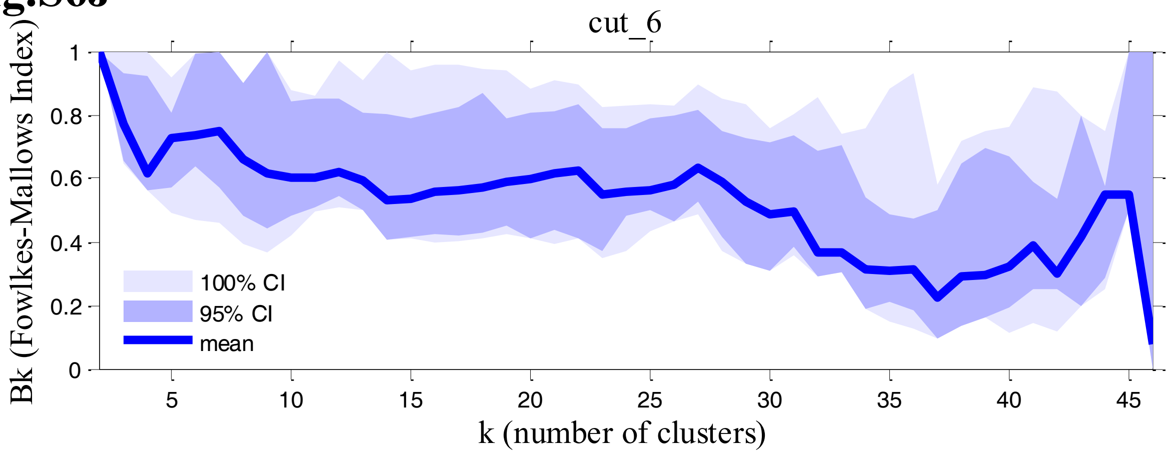

Fig.S6K

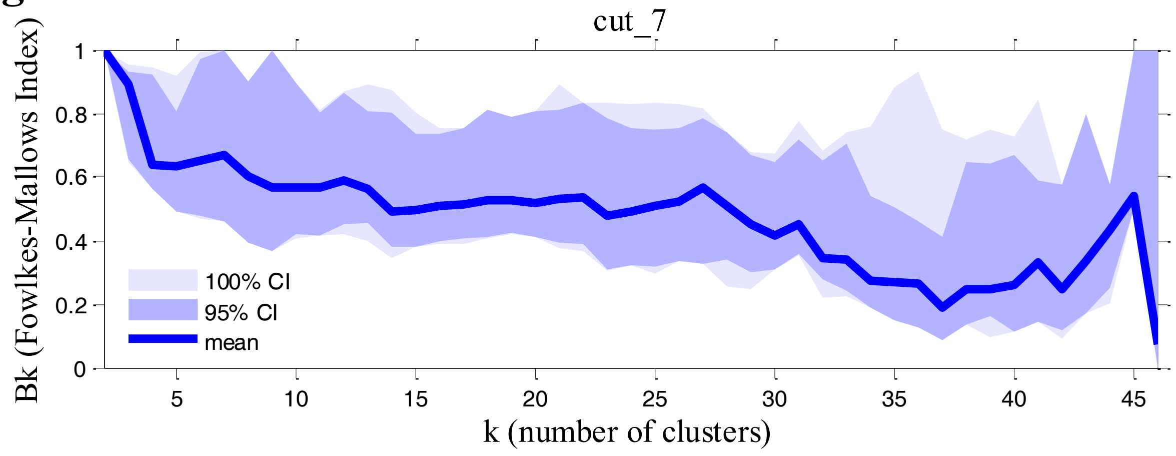

Fig.S6L

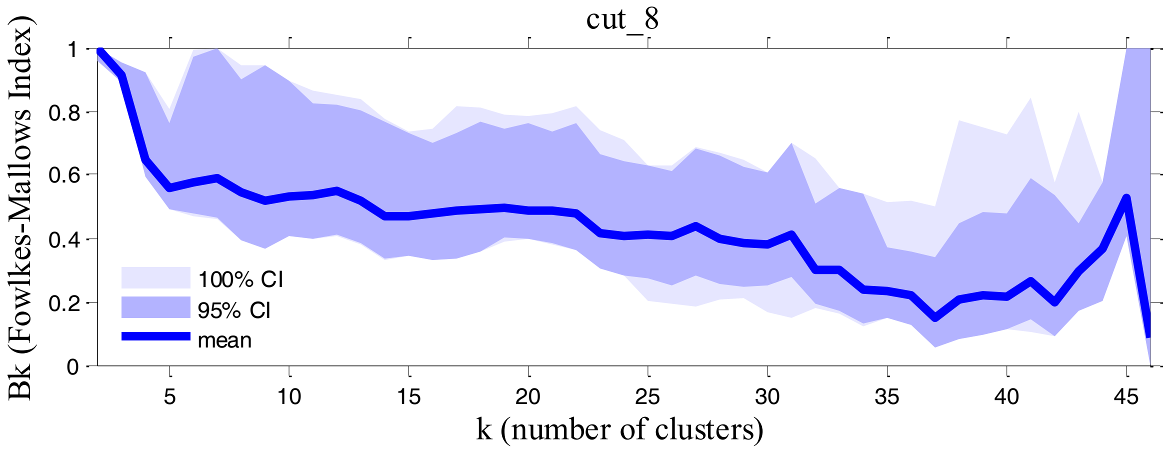

Fig.S6M

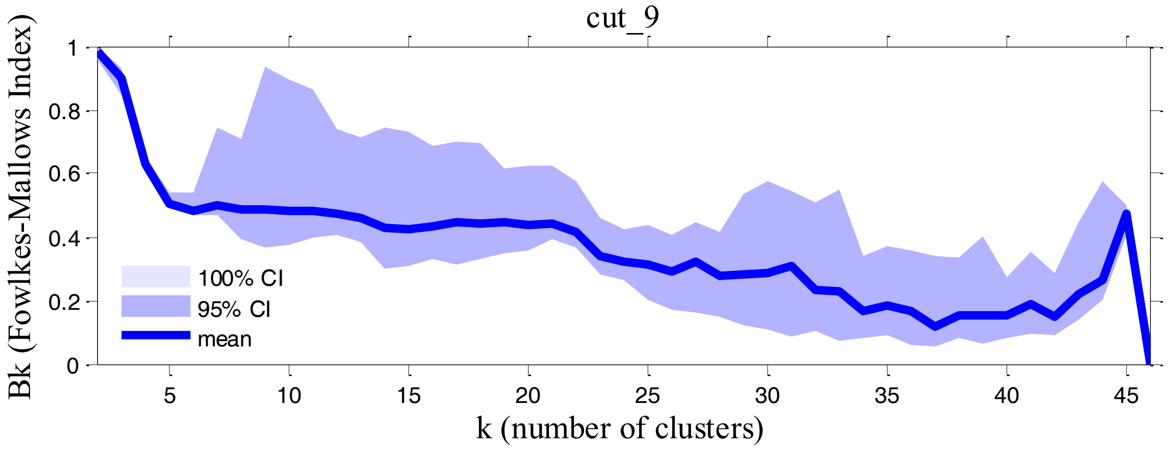

Supplementary Tables

Table S1. Identification of the TFBS-clustered regions in diverse human cells, related to Figure 2.

Table S2. Motif enrichments in each TFBS-clustered region category, related to Figure 2D.

Table S3. GO term enrichments in each TFBS-clustered region category, related to Figure 2E.

Table S4. KEGG term enrichments in each TFBS-clustered region category, related to Figure S2.

Table S5. GSC statistical analysis of the TFBS-clustered regions with TF peaks, related to Figure 3 and Figure S3.

Table S6. GSC statistical analysis of the TFBS-clustered regions with Histone peaks, related to Figure 4 and Figure S4.

Table S7. Percentage of the number of the TFBS-clustered regions in each category.

Table S1. Identification of TFBS-clustered regions in diverse human cells, Related to Figure 2

Cell type	Description	Lineage	Tissue	Karyotype	Sex	associated GSM	associated GSE	Classification by known genes															total		
								tss			utr			cds			intron			Intergenic					
								Count	Len(bp)	Percent	Count	Len(bp)	Percent	Count	Len(bp)	Percent	Count	Len(bp)	Percent	Count	Len(bp)	Percent	Count	Len(bp)	Genome coverage
Duke_8988T	pancreas adenocarcinoma (PA-TU-8988T), "established in 1985 from the liver metastasis of a primary pancreatic adenocarcinoma from a 64-year-old woman" - DSMZ	endoderm	liver	cancer	F	GSM816667	GSE32970	20,084	773.37± 428.75	14.1%	4,260	645.7± 385.05	3.0%	5,020	579.83± 338.51	3.5%	71,075	539.59± 301.58	49.8%	42,382	516.47± 273.98	29.7%	142,821	570.18± 330.36	2.7%
Duke_AoSMC	aortic smooth muscle cells	mesoderm	blood vessel	normal	U	GSM816638	GSE32970	17,041	748.38± 411.2	12.9%	2,983	591.82± 337.25	2.3%	1,571	528.76± 276.52	1.2%	70,282	511.06± 256.98	53.0%	40,687	501.49± 242.47	30.7%	132,564	540.66± 291.21	2.4%
Duke_CLL	chronic lymphocytic leukemia cell, T-cell lymphocyte	mesoderm	blood	cancer	F	GSM816664	GSE32970	14,961	825.46± 422.96	22.0%	2,010	666.25± 378.6	3.0%	1,574	590.35± 332.01	2.3%	31,471	533.31± 276.12	46.2%	18,119	511.58± 248.38	26.6%	68,135	596.92± 336.3	1.3%
Duke_Chorion	chorion cells (outermost of two fetal membranes), fetal membranes were collected from women who underwent planned cesarean delivery at term, before labor and without rupture of membranes.	extraembryonic mesoderm, trophectoderm	fetal membrane		U	GSM816628	GSE32970	20,367	737.49± 415.72	15.4%	4,300	632.42± 377.77	3.3%	3,922	656.77± 394.65	3.0%	65,480	546.51± 310.17	49.6%	38,004	520.92± 282.54	28.8%	132,073	574.67± 334.98	2.5%
Duke_FibroP	fibroblasts taken from individuals with Parkinson's disease, AG20443, AG08395 and AG08396 were pooled for this sample		skin	normal	U	GSM816626	GSE32970	21,250	726.9± 409	10.0%	4,691	576± 330.1	2.2%	3,222	525.99± 285.87	1.5%	112,458	502.57± 251.7	53.1%	70,176	492.69± 237.29	33.1%	211,797	523.79± 278.38	3.7%
Duke_Fibrobl	child fibroblast		skin	normal	F	GSM816652	GSE32970	26,361	740.86± 424.19	8.3%	7,639	610.87± 360.72	2.4%	7,181	584.34± 345.32	2.3%	173,099	544.04± 306.65	54.8%	101,551	528.18± 289.77	32.2%	315,831	557.9± 320.44	5.8%
Duke_GM12891	B-lymphocyte, lymphoblastoid, International HapMap Project, CEPH/Utah pedigree 1463, treatment: Epstein-Barr Virus transformed	mesoderm	blood		M	GSM816656	GSE32970	18,556	807.22± 430.02	18.0%	3,287	666.08± 387.4	3.2%	3,289	607.83± 351.87	3.2%	49,701	538.94± 294.49	48.2%	28,235	518.05± 271.55	27.4%	103,068	587.77± 339.72	2.0%
Duke_GM12892	B-lymphocyte, lymphoblastoid, International HapMap Project, CEPH/Utah pedigree 1463, treatment: Epstein-Barr Virus transformed	mesoderm	blood		F	GSM816657	GSE32970	19,277	785.95± 425.96	16.7%	3,658	644.41± 380.6	3.2%	4,283	573.38± 327.02	3.7%	56,272	539.6± 297.47	48.7%	32,107	519.25± 273.94	27.8%	115,597	579.6± 334.28	2.2%
Duke_GM18507	lymphoblastoid, International HapMap Project, Yoruba in Ibadan, Nigera, treatment: Epstein-Barr Virus transformed	mesoderm	blood		M	GSM816653	GSE32970	16,053	830.53± 428.07	18.9%	2,337	669.49± 380.16	2.7%	1,778	579.36± 319.76	2.1%	41,700	526.17± 270.24	49.0%	23,230	509.79± 247.31	27.3%	85,098	584.16± 328.58	1.6%
Duke_GM19238	B-lymphocyte, lymphoblastoid, International HapMap Project, Yoruba in Ibadan, Nigera, treatment: Epstein-Barr Virus transformed	mesoderm	blood		F	GSM816658	GSE32970	18,078	836.28± 436.55	16.6%	3,153	657.03± 380.27	2.9%	2,807	581.31± 331.23	2.6%	54,242	532.83± 286.62	49.9%	30,412	510.55± 258.76	28.0%	108,692	581.93± 334.68	2.1%
Duke_GM19239	B-lymphocyte, lymphoblastoid, International HapMap Project, Yoruba in Ibadan, Nigera, treatment: Epstein-Barr Virus transformed	mesoderm	blood		M	GSM816659	GSE32970	17,201	821.96± 431.22	18.7%	2,841	657.1± 382.96	3.1%	2,175	605.97± 348.01	2.4%	44,522	534.2± 287.31	48.5%	25,064	508.41± 253.47	27.3%	91,803	586.58± 336.69	1.8%
Duke_GM19240	B-lymphocyte, lymphoblastoid, International HapMap Project, Yoruba in Ibadan, Nigera, treatment: Epstein-Barr Virus transformed	mesoderm	blood		F	GSM816648	GSE32970	18,664	797.6± 430.95	13.5%	3,601	617.01± 357.13	2.6%	3,237	555.98± 312.27	2.3%	70,884	526.16± 281.69	51.2%	42,118	505.4± 255.79	30.4%	138,504	559.48± 316.72	2.6%
Duke_Gliobla	glioblastoma, these cells (aka H54 and D54) come from a surgical resection from a patient with glioblastoma multiforme (WHO Grade IV). D54 is a commonly studied glioblastoma cell line (Bao et al., 2006) that has been thoroughly described by S Bigner (1981). (PMID: 7252524)	ectoderm	brain	cancer	U	GSM816668	GSE32970	17,429	797.5± 430.35	13.8%	3,227	623.89± 363.9	2.5%	2,077	556± 309.39	1.6%	64,499	520.29± 272.59	51.0%	39,342	506.03± 251.87	31.1%	126,574	557.26± 312.42	2.3%
Duke_H9ES	embryonic stem cell (hESC) H9	inner cell mass	embryonic stem cell		F	GSM816629	GSE32970	18,743	820.05± 429.38	15.1%	3,303	634.49± 364.74	2.7%	3,181	578.98± 323.81	2.6%	59,189	504.76± 251.53	47.7%	39,616	492.78± 231.78	31.9%	124,032	553.94± 307.71	2.3%
Duke_HPDE6E6E7	pancreatic duct cells immortalized with E6E7 gene of HPV	endoderm	pancreatic duct	normal	F	GSM816639	GSE32970	16,567	805.91± 427.75	15.6%	2,475	630.96± 364.48	2.3%	1,666	564.81± 308.7	1.6%	51,176	516.66± 262.19	48.1%	34,538	498.3± 236.16	32.5%	106,422	559.14± 309.66	2.0%
Duke_HSMMemb	embryonic myoblast		muscle		U	GSM1008592	GSE32970	17,150	797.04± 422.78	17.4%	2,686	629.92± 360.17	2.7%	2,013	553.62± 297.02	2.0%	48,221	516.61± 259.77	48.9%	28,488	500.55± 238.9	28.9%	98,558	564.61± 312.71	1.8%
Duke_HTR8svn	trophoblast (HTR-8/SVneo) cell line, a thin layer of ectoderm that forms the wall of many mammalian blastulas and functions in the nutrition and implantation of the embryo	ectoderm	blastula	normal	F	GSM816644	GSE32970	16,373	793.02± 422.85	16.4%	2,308	621.68± 355.24	2.3%	1,452	551.25± 293.74	1.5%	48,577	503.28± 245.38	48.8%	30,875	489.31± 221.6	31.0%	99,585	550.03± 300.36	1.8%
Duke_HeLaS3IFNa4h	cervical carcinoma	ectoderm	cervix	cancer	F	GSM816633	GSE32970	14,105	819.99± 431.88	16.0%	2,187	625.78± 355.66	2.5%	1,374	549.75± 300.18	1.6%	42,034	507.79± 248.2	47.6%	28,658	494.44± 229.84	32.4%	88,358	556.87± 307.13	1.6%
Duke_Hepatocytes	primary hepatocytes, liver perfused by enzymes to generate single cell suspension	endoderm	liver	normal	B	GSM816663	GSE32970	18,695	762.56± 433.87	15.7%	3,730	668.18± 395	3.1%	2,393	648± 381.54	2.0%	59,238	585.65± 343.42	49.9%	34,652	551.14± 313.55	29.2%	118,708	607.29± 360.74	2.4%
Duke_Huh7.5	hepatocellular carcinoma, hepatocytes selected for high levels of hepatitis C replication	endoderm	liver	cancer	M	GSM816671	GSE32970	19,305	826.74± 443.77	14.4%	3,715	635.5± 378.69	2.8%	3,028	569.55± 331.33	2.3%	67,891	519.65± 276.3	50.6%	40,317	505.53± 256.95	30.0%	134,256	563.9± 324.47	2.5%
Duke_Huh7	hepatocellular carcinoma	endoderm	liver	cancer	M	GSM816641	GSE32970	17,605	841.98± 436	15.9%	2,916	650.87± 381.04	2.6%	2,176	577.44± 325.43	2.0%	53,923	519.92± 269.31	48.8%	33,875	507.56± 252.7	30.7%	110,495	572.03± 324.89	2.1%
Duke_IshikawaEstradiol	endometrial adenocarcinoma		uterus		F	GSM1008593	GSE32970	16,655	799.58± 417.97	15.7%	2,429	649.54± 367.75	2.3%	1,570	551.06± 295.5	1.5%	50,543	515.02± 255.91	47.8%	34,568	499.14± 232.02	32.7%	105,765	558.26± 303.65	1.9%
Duke_IshikawaTamoxifen	endometrial adenocarcinoma		uterus		F	GSM1008594	GSE32970	16,931	830.22± 427.8	16.0%	2,502	662.37± 379.38	2.4%	1,762	559.59± 299.13	1.7%	50,625	520.35± 262.22	47.9%	33,799	502.76± 238.19	32.0%	105,619	568.42± 314.7	2.0%
Duke_LNCaPAndrogen	prostate adenocarcinoma, "LNCaP clone FGC was isolated in 1977 by J.S. Horoszewicz, et al., from a needle aspiration biopsy of the left supraclavicular lymph node of a 50-year-old caucasian male (blood type B+) with confirmed diagnosis of metastatic prostate carcinoma." - ATCC. (Horoszewicz et al. LNCaP Model of Human Prostatic Carcinoma. Cancer Research 43, 1809-1818, April 1983.)	endoderm	prostate	cancer	M	GSM816634	GSE32970	17,535	842.18± 439.02	16.3%	3,247	641.12± 373.58	3.0%	2,649	571.52± 326.63	2.5%	54,680	517.82± 268.79	50.7%	29,637	493.57± 234.14	27.5%	107,748	568.97± 324.24	2.0%
Duke_MCF7Hypoxia	mammary gland, adenocarcinoma. (PMID: 4357757), newly promoted to tier 2: not in 2011 analysis	ectoderm	breast	cancer	F	GSM816670	GSE32970	17,947	766.1± 428.47	15.2%	2,951	606.17± 356.92	2.5%	2,528	548.04± 310.28	2.1%	57,827	506.27± 258.02	49.1%	36,495	498.29± 246.14	31.0%	117,748	546.8± 306.06	2.1%
Duke_Medullo	medulloblastoma (aka D721), surgical resection from a patient with medulloblastoma as described by Darrell Bigner (1997)	ectoderm	brain	cancer	U	GSM816636	GSE32970	20,239	744.25± 414.75	11.6%	4,544	610.14± 360.33	2.6%	3,945	584.47± 335.7	2.3%	92,496	529.19± 288.02	52.8%	53,867	518.88± 276.87	30.8%	175,091	554.22± 313.35	3.2%
Duke_Melano	epidermal melanocytes	ectoderm	skin	normal	U	GSM816631	GSE32970	21,976	729.42± 412.2	9.3%	5,189	578.14± 329.77	2.2%	3,451	535.71± 293.31	1.5%	125,037	503.61± 255.67	53.1%	80,031	490.71± 237.1	34.0%	235,684	522.39± 279.03	4.1%
Duke_Myometr	myometrial cells		myometrium	normal	F	GSM816630	GSE32970	18,515	787.81± 428.1	14.0%	3,371	627.66± 362.69	2.6%	2,231	584.54± 334.35	1.7%	66,405	516.64± 269.61	50.3%	41,396	502.79± 251.33	31.4%	131,918	554.34± 311.03	2.4%

Duke_Osteobl	osteoblasts (NHOst)	mesoderm	bone	normal	U	GSM816654	GSE32970	26,291	745.96±425.98	9.0%	7,153	622.88±371.28	2.5%	6,563	604.86±361.95	2.2%	157,269	541.25±304.45	53.9%	94,621	527.77±289.55	32.4%	291,897	558.75±321.82	5.4%
Duke_PanIsletD	dedifferentiated human pancreatic islets from the National Disease Research Interchange (NDRI), same source as PanIslets	endoderm	pancreas		B	GSM816666	GSE32970	19,780	767.62±422.31	12.0%	3,931	594.58±339.98	2.4%	2,924	539.91±295.11	1.8%	86,659	509.09±260.01	52.7%	51,022	497.76±241.95	31.1%	164,316	539.29±294.92	2.9%
Duke_PanIslets	pancreatic islets from 2 donors, the sources of these primary cells are cadavers from National Disease Research Interchange (NDRI) and another sample isolated as in Bucher, P. et al., Assessment of a novel two-component enzyme preparation for human islet isolation and transplantation. Transplantation 79, 917 (2005)	endoderm	pancreas	normal	M	GSM816660	GSE32970	19,679	775.89±423.26	14.3%	3,953	622.7±361.42	2.9%	3,225	590.61±338.28	2.3%	70,229	522.58±276.04	51.0%	40,734	505.84±255.68	29.6%	137,820	558.27±313.88	2.5%
Duke_ProgFib	fibroblasts, Hutchinson-Gilford progeria syndrome (cell line HGPS, HGADFN167, progeria research foundation)		skin		M	GSM816661	GSE32970	20,369	805.1±438.83	12.7%	4,176	618.51±364.85	2.6%	3,292	582.8±342.45	2.0%	83,531	516.57±272.81	52.0%	49,354	503.64±255.02	30.7%	160,722	553.17±313.91	2.9%
Duke_RWPE1	prostate epithelial		prostate	normal	M	GSM1008595	GSE32970	17,202	793.5±428.92	14.4%	2,828	624.07±362.3	2.4%	1,742	551.92±297.98	1.5%	59,193	507.16±251.88	49.5%	38,711	490.89±226.76	32.3%	119,676	546.47±299.21	2.2%
Duke_Stellate	hepatic stellate cells, liver that was perfused with collagenase and sellected for hepatic stellate cells by density gradient	endoderm	liver	normal	F	GSM816672	GSE32970	17,983	818.12±435.55	14.4%	3,379	624.12±363.3	2.7%	2,168	572.96±327.66	1.7%	65,235	517.18±268.58	52.2%	36,110	500.45±243.49	28.9%	124,875	559.54±315.08	2.3%
Duke_T47D	epithelial cell line derived from a mammary ductal carcinoma.		breast	cancer	F	GSM816673	GSE32970	17,475	822.24±435.3	16.0%	2,981	638.73±370.16	2.7%	2,136	580.46±329.53	2.0%	53,165	524.17±278.47	48.6%	33,679	503.02±251.08	30.8%	109,436	569.48±325.38	2.1%
Duke_Th0	CD4+ cells isolated from human blood and enriched for Th0 populations	mesoderm	blood	normal	B	GSM1008572	GSE32970	20,564	802.44±437.9	13.4%	4,327	617.77±369.4	2.8%	4,558	569.3±327.51	3.0%	79,641	525.25±285.98	51.7%	44,917	508.95±265.76	29.2%	154,007	561.41±323.78	2.8%
Duke_Urothelia	primary ureter cell culture of urothelial cells derived from a 12 year-old girl and immortalized by transfection with a temperature-sensitive SV-40 large T antigen gene		urothelium	normal	F	GSM1008605	GSE32970	17,543	827.11±430.44	16.5%	2,587	637.62±361.27	2.4%	1,860	558.37±307.88	1.7%	49,459	525.61±272.11	46.5%	34,894	513±253.78	32.8%	106,343	574.51±322.41	2.0%
Duke_UrotheliaUT189	primary ureter cell culture of urothelial cells derived from a 12 year-old girl and immortalized by transfection with a temperature-sensitive SV-40 large T antigen gene		urothelium	normal	F	GSM1008606	GSE32970	18,330	801.48±429	13.6%	3,069	615.84±355.53	2.3%	2,568	542.65±296.73	1.9%	65,230	523.55±274.48	48.4%	45,655	515.54±262.47	33.9%	134,852	561.08±314.09	2.5%
Duke_iPS	induced pluripotent stem cell derived from skin fibroblast	induced pluripotent stem cell derived from skin fibroblast	induced pluripotent stem cell		B	GSM816642	GSE32970	21,146	787.06±421.78	14.0%	4,514	642.41±374.4	3.0%	6,419	585.4±336.7	4.3%	72,812	534.1±292.23	48.2%	46,048	519.11±274.42	30.5%	150,939	570.39±326.01	2.8%
Duke_pHTE	primary tracheal epithelial cells	endoderm	epithelium		U	GSM816647	GSE32970	22,779	764.44±430.7	10.7%	5,308	609.87±358.6	2.5%	4,596	561.47±320.41	2.2%	110,564	522.42±282.84	51.9%	69,604	507.77±263.33	32.7%	212,851	546.55±309.01	3.8%
Th1_UW	primary Th1 T cells	mesoderm	blood		U	GSM736592	GSE29692	15,027	738.81±420.29	24.2%	1,893	660.52±381.56	3.0%	1,552	610.08±346.29	2.5%	29,126	584.27±325.31	46.9%	14,494	562.6±305.67	23.3%	62,092	619.58±355.63	1.3%
Th1wb33676984_UW	Th1 cells in vivo isolation	mesoderm	blood	normal donor is Asian, female 26 year old, primary pheresis of single normal subject	F	GSM1024749	GSE29692	13,296	729.44±415.96	15.9%	2,083	609.44±348.88	2.5%	1,251	571.05±312.55	1.5%	45,189	563.85±312.81	53.9%	22,014	546.56±294.33	26.3%	83,833	586.82±333.72	1.6%
Th1wb54553204_UW	Th1 cells in vivo isolation, donor is Caucasian, male 33 year old, primary pheresis of single normal subject	mesoderm	blood	normal	M	GSM1024753	GSE29692	14,439	804.57±442.1	16.3%	2,301	669.14±393.22	2.6%	1,358	629±369.67	1.5%	47,440	598.37±343.62	53.4%	23,278	566.25±316.7	26.2%	88,816	625.78±365.79	1.8%
Th2_UW	primary Th2 T cells	mesoderm	blood		U	GSM736502	GSE29692	15,459	747.77±421.24	22.4%	2,111	665.85±375.23	3.1%	2,062	614.62±351.8	3.0%	33,340	605.2±334.89	48.2%	16,179	586.29±317.42	23.4%	69,151	634.78±359.4	1.4%
Th2wb33676984_UW	Th2 cells in vivo isolation, donor is Asian, female 26 year old, primary pheresis of single normal subject	mesoderm	blood	normal	F	GSM1024740	GSE29692	14,220	769±430.36	17.8%	2,075	627.56±363.83	2.6%	1,411	561.43±313.29	1.8%	41,328	542.49±292.87	51.7%	20,937	522.25±270.53	26.2%	79,971	580.01±331.06	1.5%
Th2wb54553204_UW	Th2 cells in vivo isolation, donor is Caucasian, male 33 year old, primary pheresis of single normal subject	mesoderm	blood	normal	M	GSM1024739	GSE29692	15,436	790.53±438.68	15.5%	2,457	640.5±375.05	2.5%	1,857	585.32±331.85	1.9%	52,767	553.91±306.53	53.1%	26,921	530.77±279.96	27.1%	99,438	587.1±338.27	1.9%
Tregwb78495824_UW	T regulatory cells in vivo isolation	mesoderm	blood	normal donor is Causasian, female 35 year old, primary pheresis of single normal subject	F	GSM1024744	GSE29692	15,681	821.82±445.57	16.3%	2,545	660.2±388.71	2.6%	1,923	597.27±345.37	2.0%	51,235	565.92±317.84	53.2%	24,960	541.6±293.81	25.9%	96,344	604.38±352.65	1.9%
Tregwb83319432_UW	T regulatory cells in vivo isolation, donor is Caucasian, male 28 year old, primary pheresis of single normal subject	mesoderm	blood	normal	M	GSM1024741	GSE29692	17,339	855.03±452.7	14.0%	3,423	708.52±407.84	2.8%	2,228	671.79±387.47	1.8%	68,259	630.41±363.38	55.1%	32,542	608.2±348.38	26.3%	123,791	658.94±383.8	2.7%
UWDuke_A549	epithelial cell line derived from a lung carcinoma tissue. (PMID: 175022), "This line was initiated in 1972 by D.J. Giard, et al. through explant culture of lung carcinomatous tissue from a 58-year-old caucasian male." - ATCC, newly promoted to tier 2: not in 2011 analysis	endoderm	epithelium	cancer	M	GSM816649; GSM736506; GSM736580	GSE32970;GSE29692	17,509	874.86±443.49	14.4%	2,861	671.79±388.09	2.3%	2,059	583.84±334.3	1.7%	61,216	543.14±292.1	50.2%	38,336	532.08±277.99	31.4%	121,981	590.98±338.7	2.4%
UWDuke_GM12878	B-lymphocyte, lymphoblastoid, International HapMap Project - CEPH/Utah - European Caucasion, Epstein-Barr Virus	mesoderm	blood	normal	F	GSM816665; GSM736496; GSM736620	GSE32970;GSE29692	17,079	883.29±446.39	13.8%	3,300	685.13±400.96	2.7%	2,556	596.07±341.4	2.1%	68,003	564.6±316.1	54.8%	33,157	534.36±280.74	26.7%	124,095	604.23±350.67	2.5%
UWDuke_H1hESC	embryonic stem cells	inner cell mass	embryonic stem cell	normal	M	GSM816632; GSM736582	GSE32970;GSE29692	22,048	851.8±446.14	12.0%	4,689	665.93±394.2	2.6%	5,639	604.18±352.03	3.1%	91,605	541.52±299.48	49.9%	59,625	524.07±277.67	32.5%	183,606	578.21±335.1	3.5%
UWDuke_HMEC	mammary epithelial cells	ectoderm	breast	normal	U	GSM816669; GSM736552; GSM736634	GSE32970;GSE29692	24,290	793.75±436.42	9.5%	5,912	627.21±369.18	2.3%	5,858	599.08±351.89	2.3%	134,413	536.63±291.88	52.5%	85,640	521.16±272.52	33.4%	256,113	559.36±315.75	4.7%
UWDuke_HSMM	skeletal muscle myoblasts	mesoderm	muscle	normal	U	GSM816650; GSM736553; GSM736560	GSE32970;GSE29692	22,096	775.79±429.97	9.2%	5,130	610.73±358.08	2.1%	3,849	573.83±328.04	1.6%	131,453	522.24±275.85	55.0%	76,430	508.15±257.8	32.0%	238,958	543.91±300.75	4.3%
UWDuke_HSMMtube	skeletal muscle myotubes differentiated from the HSMM cell line	mesoderm	muscle	normal	U	GSM816651; GSM736530; GSM1024788	GSE32970;GSE29692	23,545	762.29±424.18	9.4%	5,743	610.41±356.71	2.3%	4,917	568.87±322.77	2.0%	135,944	527.64±283.65	54.5%	79,304	512.02±263.34	31.8%	249,453	547.54±304.84	4.5%
UWDuke_HUVEC	umbilical vein endothelial cells	mesoderm	blood vessel	normal	U	GSM816646; GSM736533; GSM736575	GSE32970;GSE29692	18,616	869.59±446.41	13.6%	3,528	650.21±378.03	2.6%	2,706	607.08±344.39	2.0%	73,077	546.86±295.91	53.6%	38,525	522.12±266.11	28.2%	136,452	587.77±336.42	2.6%
UWDuke_HeLaS3	cervical carcinoma	ectoderm	cervix	cancer	F	GSM816643; GSM736564; GSM736510	GSE32970;GSE29692	17,251	871.69±449.12	12.1%	2,995	650.45±380.41	2.1%	2,329	562.49±320.47	1.6%	70,805	532.95±280.55	49.6%	49,511	521.88±265.72	34.6%	142,891	572.95±324.23	2.7%
UWDuke_HepG2	hepatocellular carcinoma	endoderm	liver	cancer	M	GSM816662; GSM736637; GSM736639	GSE32970;GSE29692	19,767	840.39±440.17	14.1%	3,692	640.49±371.5	2.6%	3,613	552.95±305.88	2.6%	69,007	530.3±280.68	49.3%	43,934	519.86±267.25	31.4%	140,013	574.29±326.58	2.6%

UWDuke_K562	leukemia, "The continuous cell line K-562 was established by Lozzio and Lozzio from the pleural effusion of a 53-year-old female with chronic myelogenous leukemia in terminal blast crises." - ATCC	mesoderm	blood	cancer	F	GSM816655; GSM736566; GSM736629	GSE32970 ;GSE29692	19,739	816.8± 437.12	13.3%	3,763	630.43± 364.27	2.5%	3,235	565.55± 318.91	2.2%	78,210	531.66± 283.97	52.5%	43,959	519.97± 268.2	29.5%	148,906	569.24± 322.73	2.8%
UWDuke_LNCaP	prostate adenocarcinoma, "LNCaP clone FGC was isolated in 1977 by J.S. Horoszewicz, et al., from a needle aspiration biopsy of the left supraclavicular lymph node of a 50-year-old caucasian male (blood type B+) with confirmed diagnosis of metastatic prostate carcinoma." - ATCC. (Horoszewicz et al. LNCaP Model of Human Prostatic Carcinoma. Cancer Research 43, 1809-1818, April 1983.)	endoderm	prostate	cancer	M	GSM816637; GSM736565; GSM736603	GSE32970 ;GSE29692	22,442	798.69± 435.84	9.6%	5,595	643.12± 378.26	2.4%	5,598	594.1± 347.11	2.4%	124,289	535.2± 289.46	53.2%	75,563	511.33± 259.5	32.4%	233,487	556.8± 312.61	4.3%
UWDuke_MCF7	mammary gland, adenocarcinoma. (PMID: 4357757), newly promoted to tier 2: not in 2011 analysis	ectoderm	breast	cancer	F	GSM816627; GSM736581; GSM736588	GSE32970 ;GSE29692	19,121	754.66± 416.67	12.2%	3,561	585.78± 326.62	2.3%	2,696	544.95± 296.83	1.7%	79,657	505.84± 249.42	50.7%	52,157	496.68± 235.87	33.2%	157,192	535.55± 286.14	2.8%
UWDuke_NHEK	epidermal keratinocytes	ectoderm	skin	normal	U	GSM816635; GSM736545; GSM736556	GSE32970 ;GSE29692	19,646	818.37± 434.57	12.4%	3,688	645.14± 374.7	2.3%	3,129	578.86± 327.18	2.0%	81,944	539.1± 290.61	51.8%	49,912	521.53± 268.79	31.5%	158,319	571.47± 323	3.0%
UWDuke_Th1	primary Th1 T cells	mesoderm	blood		U	GSM1008604 ;GSM736592; GSM1024760	GSE32970 ;GSE29692	23,688	818.09± 446.31	10.6%	5,741	648.46± 386.62	2.6%	6,764	592.53± 350.28	3.0%	118,909	555.58± 317.01	53.3%	68,060	537.74± 299.03	30.5%	223,162	581.51± 341.37	4.3%
Uw_AG04449	fetal buttock/thigh fibroblast		skin	normal	M	GSM736562; GSM736590	GSE29692	15,684	611.9± 350.43	10.8%	2,976	518.96± 268.97	2.1%	1,642	474.9± 209.5	1.1%	80,140	488.99± 234.31	55.3%	44,459	484.75± 226.17	30.7%	144,901	501.45± 250.68	2.4%
Uw_AG04450	fetal lung fibroblast	endoderm	lung	normal	M	GSM736514; GSM736563	GSE29692	15,871	642.64± 367.55	12.5%	3,000	535.23± 283.78	2.4%	1,659	497.32± 241.23	1.3%	68,899	489.91± 230.54	54.2%	37,728	483.44± 217.54	29.7%	127,157	508.22± 254.95	2.1%
Uw_AG09309	adult toe fibroblast from apparently healthy 21 year old, "7% of the cells examined showing random chromosome loss/gain" -Coriell		skin		F	GSM736551; GSM736616	GSE29692	17,747	622.01± 358.53	10.2%	3,431	537.23± 291.63	2.0%	1,934	494.95± 237.45	1.1%	94,991	496.68± 245.2	54.7%	55,632	491.05± 236.17	32.0%	173,735	508.46± 260.15	2.9%
Uw_AG09319	gum tissue fibroblasts from apparently heathly 24 year old		gingival	normal	F	GSM736531; GSM736619	GSE29692	15,428	637.82± 365.2	12.8%	2,744	548.11± 300.42	2.3%	1,423	498.97± 240.92	1.2%	65,224	493.47± 236.56	54.0%	36,046	486.47± 223.38	29.8%	120,865	511.11± 259.36	2.0%
Uw_AG10803	abdominal skin fibroblasts from apparently heathly 22 year old, "8% of the cells examined showing random chromosome loss, 2% showing random chromosome gain, and 2% showing 69,XYY" -Coriell		skin		M	GSM736598; GSM736633	GSE29692	16,524	626.48± 361.03	11.3%	3,007	539.38± 290.99	2.1%	1,736	497± 244.82	1.2%	79,514	499.76± 249.22	54.3%	45,557	496.49± 243.38	31.1%	146,338	513.83± 266.47	2.5%
Uw_AoAF	aortic adventitial fibroblast cells	mesoderm	blood vessel	normal	F	GSM736505; GSM736583	GSE29692	16,976	619.28± 353.88	11.3%	3,162	535.22± 291.32	2.1%	1,788	494.01± 235.47	1.2%	81,733	493.13± 239.61	54.5%	46,390	486.27± 226.74	30.9%	150,049	506.18± 255.86	2.5%
Uw_BE2C	neuroblastoma, BE-C is a clone of the SK-N-BE neuroblastoma cell line (see ATCC CRL-2271) that was established in November of 1972 from a bone marrow biopsy taken from a 2-year-old individual with disseminated neuroblastoma after repeated courses of chemotherapy and radiotherapy.	ectoderm	brain	cancer	M	GSM736508; GSM736622	GSE29692	17,214	649.71± 371.51	11.6%	3,186	559.47± 314.28	2.2%	2,563	506.73± 259.69	1.7%	77,394	498.54± 246.88	52.3%	47,542	492.44± 237.7	32.1%	147,899	515.63± 268.1	2.5%
Uw_BJ	skin fibroblast, "The line was established from skin taken from normal foreskin." - ATCC. (PMID: 9916803)		skin	normal	M	GSM736518; GSM736596	GSE29692	16,003	570.75± 320.95	11.1%	2,933	511.07± 260.75	2.0%	1,372	476.18± 207.72	0.9%	79,493	483.26± 224.74	55.0%	44,774	479.43± 215.98	31.0%	144,575	492.26± 237.06	2.3%
Uw_CD20	B cells, caucasian, draw number 1, newly promoted to tier 2: not in 2011 analysis	mesoderm	blood	normal	F	GSM1024765 ;GSM1024766	GSE29692	15,579	808.4± 424.9	20.6%	2,276	655.85± 371.01	3.0%	1,676	596.9± 335.91	2.2%	36,362	534.21± 277.96	48.1%	19,748	527.59± 268.32	26.1%	75,641	594± 334.99	1.5%
Uw_CD34Mobilized	hematopoietic progenitor cells-mobilized, from donor RO01679.		blood		M	GSM1024770	GSE29692	19,425	725.3± 407.47	14.5%	3,343	570.73± 317.49	2.5%	2,620	523.73± 274.95	2.0%	69,492	491.49± 233.14	51.8%	39,315	480.72± 214.33	29.3%	134,195	524.78± 276.84	2.3%
Uw_CMK	acute megakaryocytic leukemia cells, "established from the peripheral blood of a 10-month-old boy with Down's syndrome and acute megakaryocytic leukemia (AML M7) at relapse in 1985" - DSMZ. (PMID: 3016165)	mesoderm	blood	cancer	M	GSM736607	GSE29692	15,969	741.73± 418.51	15.0%	2,858	599.54± 350.49	2.7%	2,208	548.67± 308.55	2.1%	54,152	515.1± 266.7	51.0%	31,080	496.71± 237.71	29.2%	106,267	546.75± 302.33	1.9%
Uw_Caco2	colorectal adenocarcinoma. (PMID: 1939345)	endoderm	colon	cancer	M	GSM736500; GSM736587	GSE29692	18,519	745.36± 417.98	20.3%	3,173	636.95± 370.49	3.5%	7,922	554.25± 312.66	8.7%	39,342	545.32± 300.76	43.0%	22,473	522.92± 272.53	24.6%	91,429	584.29± 335.87	1.8%
Uw_GM06990	B-lymphocyte, lymphoblastoid, International HapMap Project, CEPH/Utah, treatment: Epstein-Barr Virus transformed	mesoderm	blood		F	GSM736558; GSM736635	GSE29692	14,439	716.82± 407.72	20.7%	1,829	606.99± 341.58	2.6%	1,433	550.66± 294.94	2.1%	34,217	541.1± 288.8	49.2%	17,676	533.03± 277.42	25.4%	69,594	577.44± 324.22	1.3%
Uw_GM12864	B-lymphocyte, lymphoblastoid, International HapMap Project, CEPH/Utah pedigree 1459, treatment: Epstein-Barr Virus transformed	mesoderm	blood		M	GSM736525	GSE29692	15,413	748.06± 420.19	14.6%	2,501	615.35± 357.54	2.4%	1,823	530.71± 285.04	1.7%	56,124	535.01± 289.91	53.3%	29,495	520.25± 272.93	28.0%	105,356	563.88± 319.25	2.0%
Uw_GM12865	B-lymphocyte, lymphoblastoid, International HapMap Project, CEPH/Utah pedigree 1459, treatment: Epstein-Barr Virus transformed	mesoderm	blood		F	GSM736512; GSM736561	GSE29692	15,869	735.46± 414.68	14.0%	2,568	602.51± 352.76	2.3%	1,797	533.36± 287.13	1.6%	59,100	523.77± 278.21	52.2%	33,783	505.46± 253.4	29.9%	113,117	549.94± 306.14	2.0%
Uw_H7hESC	undifferentiated embryonic stem cells	inner cell mass			U	GSM736638; GSM736610	GSE29692	21,675	642.6± 371.11	9.5%	3,964	548.27± 306.15	1.7%	3,130	510.34± 264.93	1.4%	113,452	496.49± 246.01	49.5%	86,927	495.46± 244.58	37.9%	229,148	511.01± 264.81	3.9%
Uw_HAEpiC	amniotic epithelial cells		epithelium	normal	U	GSM736606; GSM736631	GSE29692	18,123	617.58± 354.15	10.3%	3,599	531.99± 282.11	2.0%	2,541	490.91± 233.15	1.4%	98,200	498.97± 247.21	55.5%	54,331	491.21± 235.14	30.7%	176,794	509.3± 259.95	3.0%
Uw_HAc	astrocytes-cerebellar	ectoderm	cerebellar	normal	U	GSM736538; GSM736586	GSE29692	16,958	619.25± 356.69	10.8%	3,314	530.9± 285.44	2.1%	2,088	483.17± 222.19	1.3%	85,670	488.73± 230.28	54.5%	49,026	479.68± 213.94	31.2%	157,056	500.81± 247.15	2.6%
Uw_HAh	astrocytes-hippocampal	ectoderm	brain hippocampus	normal	U	GSM736594; GSM736535	GSE29692	18,639	614.24± 356.44	10.6%	3,650	536.04± 295.25	2.1%	2,360	482.59± 224.97	1.3%	96,973	482.75± 224.66	55.0%	54,640	474.2± 207.24	31.0%	176,262	495.1± 242.4	2.9%
Uw_HAsp	astrocytes spinal cord	ectoderm	spinal cord	normal	U	GSM736537; GSM736625	GSE29692	16,647	590.54± 334.68	9.9%	3,306	518.33± 264.17	2.0%	1,660	496.7± 244.36	1.0%	95,429	486.94± 225.38	56.5%	51,947	480.88± 214.31	30.7%	168,989	495.99± 238.32	2.8%
Uw_HBMEC	brain microvascular endothelial cells	mesoderm	blood vessel	normal	U	GSM736509; GSM736554	GSE29692	17,927	594.15± 340.01	10.2%	3,429	518.33± 270.29	1.9%	2,216	480.78± 222.4	1.3%	96,301	485.75± 226.53	54.7%	56,021	478.89± 214.66	31.8%	175,894	495.19± 240.3	2.9%
Uw_HCF	cardiac fibroblasts	mesoderm	heart	normal	U	GSM736540; GSM736568	GSE29692	17,527	641.33± 370.17	11.7%	3,278	542.67± 290.45	2.2%	2,002	496.84± 244.74	1.3%	81,355	494.57± 240.89	54.4%	45,467	489.3± 231.17	30.4%	149,629	511.24± 262.28	2.5%
Uw_HCFaa	cardiac fibroblasts- adult atrial	mesoderm	heart	normal	F	GSM736494; GSM736601	GSE29692	15,847	569.91± 322.09	9.7%	3,180	511.92± 264.92	2.0%	1,818	483.8± 228.06	1.1%	91,119	486.51± 229.17	56.0%	50,886	480.73± 218.38	31.2%	162,850	493.29± 238.72	2.6%
Uw_HCM	cardiac myocytes	mesoderm	heart	normal	U	GSM736504; GSM736516	GSE29692	18,636	625.14± 360.34	11.1%	3,500	535.16± 287.24	2.1%	2,310	487.89± 231.68	1.4%	90,512	492.27± 238.21	54.1%	52,371	486.33± 226.26	31.3%	167,329	506.04± 255.87	2.8%
Uw_HCPEpiC	choroid plexus epithelial cells	ectoderm	epithelium	normal	U	GSM736569; GSM736597	GSE29692	18,255	631.49± 368.08	10.1%	3,853	536.79± 294.04	2.1%	2,638	496.94± 247.21	1.5%	100,569	498.81± 249.41	55.6%	55,404	490.09± 234.67	30.7%	180,719	510.32± 263.86	3.0%
Uw_HCT116	colorectal carcinoma (PMID: 7214343)	endoderm	colon	cancer	M	GSM736600; GSM736493	GSE29692	14,935	705.81± 396.47	16.2%	2,019	581.48± 325.14	2.2%	1,308	516.71± 263.56	1.4%	44,047	500.07± 240.85	47.8%	29,843	493.51± 230.46	32.4%	92,152	533.31± 282.18	1.6%
Uw_HConF	conjunctival fibroblast	ectoderm	eye		U	GSM736515; GSM736547	GSE29692	16,799	675.39± 388.53	13.0%	3,095	559.5± 314.39	2.4%	1,959	502.53± 246.45	1.5%	68,559	497.57± 245.17	53.2%	38,479	486.8± 225.58	29.9%	128,891	519.09± 271.88	2.2%
Uw_HEEpiC	esophageal epithelial cells	endoderm	epithelium	normal	U	GSM736532; GSM736585	GSE29692	18,622	618.81± 359.02	10.2%	3,393	536.86± 293.52	1.9%	2,467	485.21± 231.57	1.3%	94,889	490.38± 236.21	51.9%	63,545	483.43± 224.84	34.7%	182,916	501.84± 252.1	3.0%
Uw_HFF	foreskin fibroblast		foreskin	normal	M	GSM736572; GSM736602	GSE29692	18,210	646.71± 374.44	11.0%	3,628	538.59± 294.23	2.2%	2,204	494.02± 236.04	1.3%	90,392	490.06± 235.4	54.6%	51,253	483.2± 222.92	30.9%	165,687	506.27± 257.2	2.8%
Uw_HFFMyc	foreskin fibroblast cells expressing canine cMyc	fibroblast	foreskin	normal	M	GSM736524; GSM736605	GSE29692	18,702	616.7± 358.82	10.3%	3,860	533.49± 291.73	2.1%	2,417	487.84± 232.79	1.3%	98,570	492.78± 238.46	54.2%	58,338	487.31± 229.91	32.1%	181,887	504.57± 254.99	3.0%

Uw_HGF	gingival fibroblasts		gingiva	normal	U	GSM736576; GSM736579	GSE29692	15,461	645.61± 369.75	12.5%	2,797	542.71± 293.93	2.3%	1,560	489.71± 227.42	1.3%	66,793	496.94± 243.28	53.8%	37,521	489.59± 231.52	30.2%	124,132	514.18± 264.94	2.1%
Uw_HIPEpiC	iris pigment epithelial cells	ectoderm	epithelium	normal	U	GSM736589; GSM736615	GSE29692	18,299	630.8± 368.13	9.5%	3,874	532.59± 288.75	2.0%	2,567	496.11± 250.29	1.3%	109,307	504.88± 256.72	56.7%	58,625	496.66± 244.66	30.4%	192,672	514.78± 269.13	3.3%
Uw_HL60	promyelocytic leukemia cells (PMID: 276884)	mesoderm	blood	cancer	F	GSM736626; GSM736595	GSE29692	17,353	704.65± 407.61	13.4%	3,590	581.34± 336.31	2.8%	3,304	549± 311.41	2.6%	70,705	526.69± 285.68	54.8%	34,084	506.57± 255.05	26.4%	129,036	547.4± 306.45	2.3%
Uw_HMF	mammary fibroblasts		mammary		F	GSM736541; GSM736628	GSE29692	17,416	633.01± 363.89	11.4%	3,214	546.97± 302.66	2.1%	2,162	499.28± 249.24	1.4%	82,564	501.64± 249.98	54.1%	47,320	494.73± 238.58	31.0%	152,676	515.4± 266.8	2.6%
Uw_HMVECLBl	blood microvascular endothelial cells, lung-derived	mesoderm	blood vessel	normal	F	GSM736542; GSM736511	GSE29692	16,890	649.24± 375.36	11.7%	3,337	553.86± 310.08	2.3%	2,412	499.46± 249.65	1.7%	81,097	500.95± 246.96	56.2%	40,656	491.75± 233.2	28.2%	144,392	516.91± 267.8	2.5%
Uw_HMVECLLy	lymphatic microvascular endothelial cells, lung-derived	mesoderm	blood vessel	normal	F	GSM736507; GSM736627	GSE29692	16,339	703.03± 401.33	13.8%	2,970	588.28± 341.9	2.5%	2,233	518.22± 274.07	1.9%	64,212	509.39± 255.29	54.4%	32,318	493.16± 231.24	27.4%	118,072	533.89± 285.56	2.1%
Uw_HMVECdAd	adult dermal microvascular endothelial cells.		blood vessel	normal	F	GSM1024745 ;GSM102474 7	GSE29692	15,233	741.09± 412.1	15.4%	2,562	599.77± 342.87	2.6%	1,903	526.09± 277.66	1.9%	52,031	515.15± 260.29	52.7%	27,040	499.13± 237	27.4%	98,769	548.01± 298.07	1.8%
Uw_HMVECdBlAd	adult blood microvascular endothelial cells, dermal-derived	mesoderm	blood vessel	normal	F	GSM736609; GSM736523	GSE29692	17,067	658.48± 376.44	12.4%	3,143	550.03± 301.87	2.3%	2,358	499.03± 241.19	1.7%	75,554	503.48± 248.49	55.1%	39,049	493.59± 233.2	28.5%	137,171	520.94± 270.14	2.4%
Uw_HMVECdBlNeo	neonatal blood microvascular endothelial cells, dermal-derived	mesoderm	blood vessel	normal	M	GSM736571; GSM736521	GSE29692	17,548	674.35± 389.94	12.4%	3,430	548.9± 302.8	2.4%	2,541	502.81± 250.24	1.8%	78,858	502.52± 248.04	55.9%	38,782	490.57± 229.99	27.5%	141,159	521.73± 273.05	2.4%
Uw_HMVECdLyAd	adult lymphatic microvascular endothelial cells, dermal-derived	mesoderm	blood vessel	normal	F	GSM736599; GSM736591	GSE29692	15,446	692.66± 395.24	14.8%	2,620	582.39± 334.04	2.5%	1,859	518.74± 266.76	1.8%	55,588	510.67± 255.17	53.4%	28,591	497.51± 235.54	27.5%	104,104	536.01± 285.85	1.8%
Uw_HMVECdLyNeo	neonatal lymphatic microvascular endothelial cells, dermal-derived	mesoderm	blood vessel	normal	M	GSM736577; GSM736573	GSE29692	16,854	692.58± 392.72	13.3%	3,035	564.68± 316.95	2.4%	2,297	509.2± 256.37	1.8%	69,133	508.78± 255.58	54.6%	35,226	495.63± 237.16	27.8%	126,545	530.94± 282.16	2.2%
Uw_HMVECdNeo	neonatal microvascular endothelial cells (single donor), dermal-derived	mesoderm	blood vessel	normal	M	GSM736611; GSM736624	GSE29692	16,493	722.28± 408.44	14.4%	2,914	585.61± 335.58	2.5%	2,316	525.21± 274.61	2.0%	61,146	505.84± 250.46	53.4%	31,742	491.56± 228.55	27.7%	114,611	535.45± 287.09	2.0%
Uw_HNPCEpiC	non-pigment ciliary epithelial cells		epithelium	normal	U	GSM736550; GSM736621	GSE29692	18,045	618.12± 360.41	9.8%	3,617	533.09± 289.35	2.0%	2,340	493.73± 237.32	1.3%	101,174	499.81± 249.79	55.2%	58,239	492.75± 238.13	31.8%	183,415	509.78± 262.47	3.1%
Uw_HPAEC	pulmonary artery endothelial cells.	mesoderm	blood vessel	normal	F	GSM1024763	GSE29692	14,933	706.35± 398.9	15.0%	2,425	574.12± 321.56	2.4%	1,757	515.82± 268.49	1.8%	52,541	512.16± 256.39	52.7%	27,992	500.56± 242.96	28.1%	99,648	539.58± 289.66	1.8%
Uw_HPAF	pulmonary artery fibroblasts	mesoderm	blood vessel	normal	U	GSM736555; GSM736614	GSE29692	18,271	634.77± 363.43	11.2%	3,498	534.17± 281.73	2.1%	2,402	494.82± 243.62	1.5%	88,359	488.64± 232.32	54.2%	50,402	481.46± 218.65	30.9%	162,932	503.88± 252.33	2.7%
Uw_HPF	pulmonary fibroblasts isolated from lung tissue		lung	normal	U	GSM736503; GSM736574	GSE29692	16,667	643.14± 366.41	12.6%	3,133	541.52± 292.74	2.4%	1,768	504.73± 256.54	1.3%	70,982	491.13± 232.26	53.7%	39,716	480.31± 213.57	30.0%	132,266	508.41± 255.32	2.2%
Uw_HPdLF	periodontal ligament fibroblasts		epithelium	normal	M	GSM736528; GSM736632	GSE29692	16,505	618.42± 355.59	11.1%	3,196	524.73± 276.7	2.1%	1,693	484.92± 225.5	1.1%	82,426	487.12± 230.66	55.3%	45,324	481.29± 219.01	30.4%	149,144	500.66± 248.91	2.5%
Uw_HRCEpiC	renal cortical epithelial cells	mesoderm	epithelium	normal	U	GSM736549; GSM736557	GSE29692	17,784	611.26± 353.37	10.7%	3,129	524.36± 278.87	1.9%	1,802	481.38± 215.44	1.1%	90,423	496.44± 242.53	54.2%	53,788	489.44± 230.31	32.2%	166,926	506.78± 256.02	2.8%
Uw_HRE	renal epithelial cells	mesoderm	epithelium	normal	U	GSM736527; GSM736548	GSE29692	17,967	622.33± 359.71	11.1%	3,059	539.26± 295.91	1.9%	1,916	486.92± 220.64	1.2%	85,738	496.91± 242.05	53.1%	52,923	489.5± 229.95	32.7%	161,603	509.11± 258.21	2.7%
Uw_HRGEC	renal glomerular endothelial cells	mesoderm	kidney	normal	U	GSM736499; GSM736618	GSE29692	15,482	639.26± 373.33	13.4%	2,734	534.42± 289.24	2.4%	1,569	497.47± 244.27	1.4%	62,708	505.32± 250.81	54.3%	32,951	491.69± 228.68	28.5%	115,444	519.97± 269.9	2.0%
Uw_HRPEpiC	retinal pigment epithelial cells	ectoderm	epithelium	normal	U	GSM736623; GSM736630	GSE29692	18,725	629.61± 366.68	9.6%	3,455	537.82± 293.85	1.8%	1,954	500.56± 249.03	1.0%	105,248	501.8± 250.93	53.7%	66,471	492.37± 235.62	33.9%	195,853	511.44± 263.06	3.3%
Uw_HVMF	villous mesenchymal fibroblast cells		connective	normal	U	GSM736491; GSM736534	GSE29692	16,069	615.05± 352.64	10.9%	3,154	536.31± 288.63	2.1%	1,575	490.57± 234.3	1.1%	83,152	499.65± 246.56	56.7%	42,812	488.01± 227.63	29.2%	146,762	509.58± 258.81	2.5%
Uw_Jurkat	T lymphoblastoid derived from an acute T cell leukemia, "The Jurkat cell line was established from the peripheral blood of a 14 year old boy by Schneider et al., and was originally designated JM." - ATCC. (PMID: 68013)	mesoderm	blood	cancer	M	GSM736492; GSM736501	GSE29692	16,379	664.22± 385.03	12.2%	2,954	554.74± 310.1	2.2%	2,022	519.21± 275.26	1.5%	69,757	500.58± 248.17	52.1%	42,884	485.77± 225.85	32.0%	133,996	517.32± 270.01	2.3%
Uw_MonocytesCD14RO01746	Monocytes-CD14+ are CD14-positive cells from human leukapheresis production, from donor RO 01746 (draw 1 ID is RO 01746, draw 2 ID is RO 01826), Monocytes-CD14+_RO01746 and Monocytes-CD14+_RO01826 are being used as replicates, newly promoted to tier 2: not in 2011 analysis	mesoderm	monocytes	normal	F	GSM1024791	GSE29692	16,306	722.38± 405.45	15.3%	2,648	586.7± 331.57	2.5%	1,935	538.23± 286.84	1.8%	54,509	525.19± 278.4	51.1%	31,176	514.35± 264.87	29.3%	106,574	553.95± 307.89	1.9%
Uw_NB4	acute promyelocytic leukemia cell line. (PMID: 1995093)	mesoderm	blood	cancer	U	GSM736529; GSM736604	GSE29692	16,275	728.86± 415.27	14.1%	2,718	597.3± 351.5	2.3%	1,752	544.81± 304.65	1.5%	59,098	508.51± 256.94	51.0%	35,928	497.64± 241.05	31.0%	115,771	538.75± 294	2.1%
Uw_NHA	astrocytes (also called Astrocy)	ectoderm	brain	normal	U	GSM736544; GSM736584	GSE29692	17,341	601.98± 341.1	10.4%	3,126	530.43± 285.66	1.9%	2,071	487.34± 233.65	1.2%	91,624	497.07± 244.69	55.1%	52,020	489.14± 231.62	31.3%	166,182	506.04± 255.48	2.8%
Uw_NHDFAd	adult dermal fibroblasts	mesoderm	skin	normal	F	GSM736567; GSM736520	GSE29692	18,683	616.6± 359.31	9.4%	3,742	529.8± 285.78	1.9%	2,232	494.58± 243.85	1.1%	108,779	499.47± 251.57	54.8%	65,048	494.67± 243.19	32.8%	198,484	509.44± 263.9	3.3%
Uw_NHDFneo	neonatal dermal fibroblasts	mesoderm	skin	normal	U	GSM736498; GSM736546	GSE29692	17,024	614.93± 353.46	10.5%	3,300	532.06± 289.15	2.0%	1,643	493.76± 238.01	1.0%	88,922	494.68± 241.91	54.7%	51,818	491.03± 235.15	31.8%	162,707	506.85± 257.54	2.7%
Uw_NHLF	lung fibroblasts	endoderm	lung	normal	U	GSM736536; GSM736612	GSE29692	17,725	595.3± 338.64	9.9%	3,294	526.54± 281.09	1.8%	2,237	480.75± 218.39	1.2%	99,730	497.83± 246.67	55.6%	56,428	490.11± 233.58	31.5%	179,414	505.34± 255.49	3.0%
Uw_NT2D1	malignant pluripotent embryonal carcinoma (NTera-2), "The NTERA-2 cl.D1 cell line is a pluripotent human testicular embryonal carcinoma cell line derived by cloning the NTERA-2 cell line." - ATCC. (PMID: 6694356)	inner cell mass	testis	cancer	M	GSM1024751 ;GSM102479 5	GSE29692	19,274	709.91± 404.92	12.5%	3,214	572.35± 323.9	2.1%	2,459	513.97± 268.98	1.6%	73,585	488.62± 234.5	47.9%	55,168	481.91± 221.92	35.9%	153,700	516.12± 271.06	2.6%
Uw_PANC1	pancreatic carcinoma, (PMID: 1140870) PANC-1 was established from a pancreatic carcinoma, which was extracted via pancreatico-duodenectomy specimen from a 56-year-old Caucasian individual. Malignancy of this cell line was verified via in vitro and in vivo assays.	endoderm	pancreas	cancer	M	GSM736517; GSM736519	GSE29692	14,982	630.96± 370.75	15.3%	2,212	552.74± 317.82	2.3%	1,242	510.38± 271.64	1.3%	49,086	497.56± 248.02	50.2%	30,311	485.04± 229.14	31.0%	97,833	515.52± 271.89	1.7%
Uw_PrEC	prostate epithelial cell line	epithelial	prostate	normal	U	GSM1024742 ;GSM102474	GSE29692	15,931	609.9± 350.72	11.0%	2,618	532.87± 285.33	1.8%	1,814	477.07± 209.23	1.3%	73,222	493.69± 238.94	50.7%	50,908	490.39± 233.56	35.2%	144,493	505.84± 255.17	2.4%
Uw_RPTEC	renal proximal tubule epithelial cells		epithelium	normal	U	GSM736539; GSM736543	GSE29692	16,582	603.31± 349.14	11.2%	2,661	523.59± 275.64	1.8%	1,547	482.87± 216.86	1.0%	79,068	487.45± 229.14	53.5%	48,049	479.3± 213.84	32.5%	147,907	498.4± 244.7	2.4%
Uw_SAEC	small airway epithelial cells	endoderm	epithelium	normal	U	GSM736608; GSM736617	GSE29692	17,807	601.44± 346.05	10.3%	3,188	530.93± 287.28	1.8%	2,079	478.67± 213.34	1.2%	89,758	492.87± 239.41	51.9%	60,251	487.44± 229.58	34.8%	173,083	502.68± 252.22	2.9%
Uw_SKMC	skeletal muscle cells	mesoderm	muscle	normal	U	GSM736497; GSM736593	GSE29692	17,419	603.09± 344.79	9.8%	3,334	522.85± 275.38	1.9%	2,168	481.63± 218.21	1.2%	96,654	497.67± 247.91	54.1%	59,035	492.15± 238.59	33.1%	178,610	506.4± 258.25	3.0%
Uw_SKNMC	neuroepithelioma cell line derived from a metastatic supra-orbital human brain tumor, "SK-N-MC was isolated in September of l971 and was found to have moderate dopamine - beta - hydroxylase activity as well as formaldehyde induced fluorescence indicative of intracellular catecholamines." - ATCC. (Biedler, et al. Morphology and Growth, Tumorigenicity, and Cytogenetics of Human Neuroblastoma Cells in Continuous Culture. Cancer Research 33, 2643-2652, November 1973.)	ectoderm	brain	cancer	F	GSM736522; GSM736570	GSE29692	13,672	601.1± 340.19	11.0%	2,241	544.19± 300.01	1.8%	1,052	497.82± 245.4	0.8%	63,191	499± 243.17	50.7%	44,449	497.73± 242.11	35.7%	124,605	510.55± 258.4	2.1%
Uw_SKNSHRA	neuroblastoma cell line, treatment: differentiated with retinoic acid, (Biedler, et al. Morphology and Growth, Tumorigenicity, and Cytogenetics of Human Neuroblastoma Cells in Continuous Culture. Cancer Research 33, 2643-2652, November 1973.)	ectoderm	brain	cancer	F	GSM736559; GSM736578	GSE29692	15,208	747.41± 411.97	22.4%	1,894	644.76± 370.85	2.8%	2,027	572.11± 322.69	3.0%	31,244	531.9± 277.89	46.0%	17,570	509.27± 247.64	25.9%	67,943	578.63± 324.11	1.3%

Uw_Th2	primary Th2 T cells	mesoderm	blood		U	GSM736502; GSM1024792	GSE29692	15,259	704.54± 399.9	22.4%	2,096	604.1± 342.15	3.1%	1,911	552.19± 303.46	2.8%	32,740	538.59± 283.68	48.1%	16,084	528.71± 270.53	23.6%	68,090	575.84± 320.87	1.3%
Uw_WERIRb1	retinoblastoma (PMID: 844036)	ectoderm	eye	cancer	F	GSM736495; GSM736636	GSE29692	17,819	609.49± 344.82	10.8%	3,117	533.32± 286.91	1.9%	1,978	509.99± 259.56	1.2%	84,453	495.03± 240.94	51.1%	58,015	489.02± 232.1	35.1%	165,382	506.15± 255.03	2.8%
Uw_WI38	embryonic lung fibroblast cells, hTERT immortalized, includes Raf1 construct	endoderm	embryonic lung	normal	F	GSM736613; GSM736526	GSE29692	16,382	630.34± 361.38	11.5%	3,218	543.07± 296.27	2.3%	1,735	496.39± 242.5	1.2%	79,457	494.75± 240.16	55.6%	42,202	487.27± 227.42	29.5%	142,994	509.18± 258.71	2.4%
Uw_WI38TamoxifenTamoxifen	embryonic lung fibroblast cells, hTERT immortalized, includes Raf1 construct	endoderm	embryonic lung	normal	F	GSM931362; GSM931361	GSE29692	17,826	609.17± 351.58	10.2%	3,908	534.4± 292.17	2.2%	2,040	501.57± 249.86	1.2%	96,743	505.8± 258.38	55.4%	54,212	499.73± 249.47	31.0%	174,729	515.05± 269.36	3.0%

Table S2. Motif enrichments at varying complexity category, Related to Figure 2D

enrichment / TF name	(-log(Pvalue))									
	TC0	TC1	TC2	TC3	TC4	TC5	TC6	TC7	TC8	TC9
LHX1	0	0	0	0	0	0	0	0	0	5
HOMEZ	0	0	0	0	0	0	0	0	0	6
FOXI1	0	0	0	0	0	0	0	0	0	7
ESX1	0	0	0	0	0	0	0	0	0	20
FOXL1	0	0	0	0	0	0	0	0	0	20
LMX1A	0	0	0	0	0	0	0	0	0	20
HOXC4	0	0	0	0	0	0	0	1	0	12
IRF2	0	0	0	0	0	0	0	2	0	0
LHX3	0	0	0	0	0	0	0	3	0	19
HIF1A	0	0	0	0	0	0	0	4	3	0
MYBL2	0	0	0	0	0	0	2	1	2	1
ATF6	0	0	0	0	0	0	8	0	20	19
GABPB1	0	0	0	0	0	0	7	20	17	20
ZFHX3	0	0	0	0	0	0	9	2	20	2
FOXJ1	0	0	0	0	0	0	9	6	0	1
VSX2	0	0	0	0	0	1	0	0	5	13
PRDM1	0	0	0	0	0	1	1	0	1	20
POU3F4	0	0	0	0	0	1	3	4	4	20
ZNF263	0	0	0	0	0	2	0	0	0	0
ELK1	0	0	0	0	0	0	20	20	20	20
FOXA2	0	0	0	0	0	2	4	7	2	20
HOXA5	0	0	0	0	0	3	0	0	0	14
CDC5L	0	0	0	0	0	3	0	0	0	20
ZNF148	0	0	0	0	0	3	5	0	0	15
POU3F2	0	0	0	0	0	4	0	0	6	20
GBX2	0	0	0	0	0	4	0	17	0	20
EN2	0	0	0	0	0	4	0	20	0	20
LHX8	0	0	0	0	0	8	0	0	0	15
HOXA1	0	0	0	0	0	8	0	20	0	20
IL6	0	0	0	0	0	9	0	0	0	4
MSX2	0	0	0	0	0	15	0	5	0	20
ALX3	0	0	0	0	0	15	20	20	4	20
FOXO3	0	0	0	0	3	2	12	11	13	20
ATF2	0	0	0	0	2	20	20	13	1	13
DLX3	0	0	0	0	6	0	3	4	0	20
CDX1	0	0	0	0	7	10	6	6	20	20
HMX1	0	0	0	1	0	0	0	19	5	20
MTF1	0	0	0	1	0	1	2	3	2	1
ELF5	0	0	0	0	10	2	0	0	0	0
HMX3	0	0	0	1	0	2	3	3	8	20
DMRT2	0	0	0	2	0	0	0	0	0	0
DMRT3	0	0	0	2	0	1	0	0	0	0
IRF9	0	0	0	2	6	18	20	20	20	20
EVX2	0	0	0	4	0	11	0	20	20	20
LHX6	0	0	0	6	0	20	10	6	0	20
VDR	0	0	0	8	0	2	2	0	0	3
BARHL1	0	0	0	14	1	20	11	4	4	20
MEF2A	0	0	0	20	0	0	0	1	0	0
NR0B1	0	0	0	20	0	0	5	0	0	0

PRRX1	0	0	2	0	0	9	11	20	20	20
E4F1	0	0	0	20	0	11	20	7	20	20
HOXB4	0	0	2	3	6	18	13	20	5	20
MSX1	0	0	2	5	20	20	1	18	6	20
MNX1	0	0	2	8	13	9	0	10	20	20
ISX	0	0	1	20	0	20	0	20	0	20
HOXC5	0	0	1	20	20	20	20	20	20	20
GSC	0	0	3	4	0	20	13	0	0	0
HOXD13	0	0	3	3	16	20	20	20	17	20
DLX4	0	0	4	1	20	20	19	20	20	20
GZF1	0	0	5	0	3	6	0	0	0	0
HOXA3	0	0	3	20	20	20	12	20	20	20
HSF2	0	0	5	8	0	0	0	0	5	0
KLF12	0	0	7	0	0	0	0	0	0	0
GBX1	0	0	9	4	9	20	0	9	0	20
HOXB9	0	1	0	0	1	16	3	10	7	20
FLI1	0	1	0	0	4	0	1	20	20	6
MAX	0	1	0	0	10	20	20	9	14	20
EPAS1	0	1	0	0	16	14	0	0	0	0
ALX4	0	0	9	12	0	0	2	7	9	20
ALX1	0	0	12	14	0	11	8	20	6	20
LHX4	0	1	4	9	12	16	0	19	8	20
IRF8	0	0	18	0	0	0	6	0	0	0
LHX9	0	0	20	1	8	14	9	20	20	20
REL	0	0	20	3	20	3	0	0	0	0
ETS2	0	0	19	18	5	20	20	20	20	20
HSF1	0	0	20	20	20	0	0	0	20	5
DLX1	0	2	2	20	20	13	20	20	20	20
ETS1	0	1	15	6	3	0	20	20	0	2
IKZF1	0	3	0	9	0	0	0	0	0	0
T	0	4	12	0	14	20	7	10	20	0
LBX2	0	5	0	20	20	20	18	20	6	20
LHX2	0	5	9	0	6	20	20	20	12	20
HOXA6	0	8	9	13	20	20	20	20	20	20
DLX2	0	8	19	20	20	20	20	20	20	20
HOXB7	0	9	18	20	20	20	20	20	20	20
FOXD1	0	10	20	2	20	17	20	20	20	20
RFX1	1	0	20	10	14	0	3	9	9	0
ATF1	1	0	19	20	20	0	5	20	0	0
ARNT	0	11	20	20	20	20	20	20	20	20
HOXA9	0	12	16	20	20	20	20	20	20	20
HOXD1	0	14	20	20	20	20	20	20	20	20
KLF4	1	6	3	4	20	3	4	3	3	3
MYB	0	20	0	0	0	0	0	0	0	0
ZBTB7A	0	20	0	0	17	0	0	0	19	0
MAF	0	20	0	1	20	0	8	20	20	20
NR1H2	2	0	0	4	20	20	1	18	20	20
HOXD8	0	18	20	20	20	20	20	20	20	20
GLI2	0	20	16	0	0	0	2	20	0	20
GLI1	0	20	19	0	2	0	0	20	0	20
DLX5	0	20	20	7	20	20	4	20	6	20
ZNF354C	0	20	20	20	0	2	0	0	0	0
FOXJ2	0	20	20	20	20	20	4	20	0	12
ARID3A	0	20	20	20	20	20	20	20	20	20

BARX2	0	20	20	20	20	20	20	20	20	20
HOXB8	0	20	20	20	20	20	20	20	20	20
HOXA7	3	0	0	0	0	0	0	0	0	0
HES1	3	0	0	0	0	20	0	0	0	0
MZF1	3	1	6	0	1	17	0	0	17	0
FOXD3	1	20	20	20	20	20	20	20	20	20
HOXC8	1	20	20	20	20	20	20	20	20	20
IRX6	2	15	1	10	3	20	11	18	0	10
EN1	3	5	3	3	2	3	3	5	4	20
FOXO4	2	14	20	15	20	20	20	18	20	20
HOXA2	2	20	20	20	20	20	20	20	20	20
SPIB	4	5	4	2	3	5	5	4	7	13
ATF5	4	8	4	0	4	20	20	0	0	0
NR6A1	6	5	20	20	6	11	10	16	20	20
GSX2	6	11	20	20	20	20	20	20	20	20
ELF3	6	20	16	8	0	1	20	0	0	0
ESRRB	6	20	20	20	19	20	20	20	20	0
HLF	7	20	20	20	20	20	20	20	20	0
HOXB5	7	20	20	20	20	20	20	20	20	20
TFAP2C	9	2	14	0	0	0	0	0	0	0
HOXB13	8	12	20	20	20	20	20	20	20	20
TFCP2L1	8	20	10	0	20	20	20	0	7	20
DMRT1	9	20	0	3	0	2	0	0	0	0
ELF1	11	8	20	20	15	20	20	20	20	3
BRCA1	11	14	14	14	15	18	15	13	12	19
NFE2L2	12	12	7	2	7	4	3	5	5	2
EMX2	14	0	17	4	5	20	20	20	20	20
HOXA4	13	20	20	20	20	20	20	20	20	20
HOXC6	13	20	20	20	20	20	20	20	20	20
TFCP2	14	15	19	16	20	20	20	20	9	10
GFI1	14	18	20	5	11	0	6	9	0	5
CREB1	14	20	20	20	20	20	20	20	20	20
ATF4	15	20	20	20	20	20	20	20	14	20
E2F1	16	20	20	20	20	20	20	20	20	20
HOXC9	16	20	20	20	20	20	20	20	20	20
HIC1	17	20	2	20	0	20	0	0	0	0
E2F6	17	20	20	20	20	20	20	20	20	20
IRF6	17	20	20	20	20	20	20	20	20	20
CEBPD	20	1	20	10	7	3	2	20	3	18
EVX1	20	1	20	17	20	20	20	20	20	20
CTCF	20	5	20	4	20	20	20	20	20	20
USF2	20	9	10	0	1	0	20	0	20	0
ESR1	20	8	20	4	20	20	20	20	5	0
TFAP4	20	13	7	20	20	20	20	16	20	20
AR	20	17	20	20	20	20	20	20	20	20
BPTF	20	18	15	8	9	4	6	2	1	4
DMRTA2	20	20	2	1	2	15	0	8	5	20
AIRE	20	20	5	20	11	20	20	0	0	0
ESRRA	20	20	6	20	20	0	0	3	0	0
DDIT3	20	19	20	0	0	2	2	0	0	0
GCM1	20	20	11	20	20	5	20	20	3	18
BHLHE40	20	20	20	0	20	20	20	7	15	0
GFI1B	20	20	20	4	20	7	1	3	19	10
LMO2	20	20	20	6	20	20	6	20	5	0

GLI3	20	20	20	9	20	2	5	20	0	20
GATA2	20	20	20	11	18	20	16	5	20	20
GATA4	20	20	20	13	20	0	0	20	8	20
HNF4A	20	20	20	15	20	10	20	4	13	5
TFAP2A	20	20	20	17	20	18	20	4	2	0
MAFA	20	20	20	18	20	20	20	9	20	20
TOPORS	20	20	20	20	6	20	8	20	14	11
HBP1	20	20	20	20	6	20	9	20	20	20
HMBOX1	20	20	20	19	20	20	20	16	17	20
NR2F6	20	20	20	20	20	0	5	20	0	0
NFIC	20	20	20	20	20	9	20	20	20	20
RUNX3	20	20	20	20	20	15	20	20	0	3
HNF4G	20	20	20	20	20	20	3	20	20	2
LEF1	20	20	20	20	20	19	20	14	13	17
JUNB	20	20	20	20	20	20	16	10	6	11
IRX2	20	20	20	20	20	20	17	11	1	0
RUNX1	20	20	20	20	20	20	20	0	20	0
IRX3	20	20	20	20	20	20	20	2	1	2
BACH2	20	20	20	20	20	20	20	4	20	0
ZBTB33	20	20	20	20	20	20	20	5	17	20
NFIL3	20	20	20	20	20	20	20	8	20	20
NR5A2	20	20	20	20	20	20	20	13	20	0
TCF7	20	20	20	20	20	20	20	14	20	20
TCF4	20	20	20	20	20	20	20	20	14	2
BSX	20	20	20	20	20	20	20	20	16	20
CREM	20	20	20	20	20	20	20	20	20	2
DBP	20	20	20	20	20	20	20	20	20	11
ATF3	20	20	20	20	20	20	20	20	20	20
ATOH1	20	20	20	20	20	20	20	20	20	20
BARX1	20	20	20	20	20	20	20	20	20	20
BCL6	20	20	20	20	20	20	20	20	20	20
CDX2	20	20	20	20	20	20	20	20	20	20
CEBPA	20	20	20	20	20	20	20	20	20	20
CEBPB	20	20	20	20	20	20	20	20	20	20
CEBPG	20	20	20	20	20	20	20	20	20	20
CRX	20	20	20	20	20	20	20	20	20	20
CUX1	20	20	20	20	20	20	20	20	20	20
DBX1	20	20	20	20	20	20	20	20	20	20
DBX2	20	20	20	20	20	20	20	20	20	20
DMRTA1	20	20	20	20	20	20	20	20	20	20
DMRTC2	20	20	20	20	20	20	20	20	20	20
EBF2	20	20	20	20	20	20	20	20	20	20
FOSL1	20	20	20	20	20	20	20	20	20	20
FOXA1	20	20	20	20	20	20	20	20	20	20
FOXM1	20	20	20	20	20	20	20	20	20	20
FOXO1	20	20	20	20	20	20	20	20	20	20
FOXP3	20	20	20	20	20	20	20	20	20	20
GATA1	20	20	20	20	20	20	20	20	20	20
GATA3	20	20	20	20	20	20	20	20	20	20
GATA6	20	20	20	20	20	20	20	20	20	20
HLX	20	20	20	20	20	20	20	20	20	20
HMGA1	20	20	20	20	20	20	20	20	20	20
HMGA2	20	20	20	20	20	20	20	20	20	20
HNF1A	20	20	20	20	20	20	20	20	20	20

HNF1B	20	20	20	20	20	20	20	20	20	20
HOXA10	20	20	20	20	20	20	20	20	20	20
HOXA13	20	20	20	20	20	20	20	20	20	20
HOXB3	20	20	20	20	20	20	20	20	20	20
HOXB6	20	20	20	20	20	20	20	20	20	20
HOXD10	20	20	20	20	20	20	20	20	20	20
HOXD3	20	20	20	20	20	20	20	20	20	20
IKZF2	20	20	20	20	20	20	20	20	20	20
IRF1	20	20	20	20	20	20	20	20	20	20
IRX4	20	20	20	20	20	20	20	20	20	20
IRX5	20	20	20	20	20	20	20	20	20	20
ISL1	20	20	20	20	20	20	20	20	20	20
ISL2	20	20	20	20	20	20	20	20	20	20
JUN	20	20	20	20	20	20	20	20	20	20
MECOM	20	20	20	20	20	20	20	20	20	20
MEIS1	20	20	20	20	20	20	20	20	20	20
MEIS2	20	20	20	20	20	20	20	20	20	20
MEIS3	20	20	20	20	20	20	20	20	20	20
MEOX1	20	20	20	20	20	20	20	20	20	20
MYC	20	20	20	20	20	20	20	20	20	20
MYOD1	20	20	20	20	20	20	20	20	20	20
MYOG	20	20	20	20	20	20	20	20	20	20
NHLH1	20	20	20	20	20	20	20	20	20	20
NR1H4	20	20	20	20	20	20	20	20	20	20
NR2F1	20	20	20	20	20	20	20	20	20	20
NR2F2	20	20	20	20	20	20	20	20	20	20
NR3C1	20	20	20	20	20	20	20	20	20	20
PDX1	20	20	20	20	20	20	20	20	20	20
POU5F1	20	20	20	20	20	20	20	20	20	20
RAX	20	20	20	20	20	20	20	20	20	20
SOX2	20	20	20	20	20	20	20	20	20	20
TCF12	20	20	20	20	20	20	20	20	20	20
TCF3	20	20	20	20	20	20	20	20	20	20
ZEB1	20	20	20	20	20	20	20	20	20	20
BACH1	20	20	20	20	20	20	20	20	20	20

Table S3. GO term enrichments at varying complexity category, Related to Figure 2E

	GO term \ kind	TC0	TC1	TC2	TC3	TC4	TC5	TC6	TC7	TC8	TC9
	GO:0007610~behavior	6.58E-09	7.99E-07	1	1	1	1	1	1	1	1
	GO:0007267~cell-cell signaling	4.90E-12	9.22E-07	1	1	1	1	1	1	1	1
	GO:0006928~cell motion	5.76E-07	1.26E-08	2.27E-05	4.86E-07	1	1	1.73E-06	1	1	1
	GO:0016477~cell migration	1.03E-06	4.21E-08	1.30E-05	1	1	1	1.24E-07	1	1	1
	GO:0045597~positive regulation of cell differentiation	2.81E-09	1.04E-08	8.03E-06	1	1	1	6.90E-05	5.15E-05	1	1
	GO:0001822~kidney development	1.38E-05	5.95E-08	1	1	1	1	1	1	1	1
	GO:0048598~embryonic morphogenesis	1.28E-06	2.69E-09	5.71E-08	1.84E-07	1	1	2.62E-05	9.24E-06	5.33E-05	1
	GO:0001944~vasculature development	1.34E-05	1	1.09E-07	1	1	1	1	1	1	1
	GO:0035295~tube development	4.67E-08	1.59E-08	9.01E-08	1	1	1	6.96E-07	1	1	1
	GO:0043583~ear development	2.17E-05	9.23E-05	1	2.57E-07	1	1	1.21E-05	1	1	1
development	GO:0001501~skeletal system development	1.41E-08	1.14E-07	1	4.05E-06	1	1	1	1	1	1
	GO:0048732~gland development	4.00E-08	3.47E-05	1	5.11E-06	1	1	1	1	1	1
	GO:0000902~cell morphogenesis	3.12E-05	2.53E-08	1	6.37E-07	4.17E-05	1	1	1	1	1
	GO:0048858~cell projection morphogenesis	2.60E-06	3.29E-07	1	2.83E-08	4.55E-07	1.04E-05	1	1	1	1
	GO:0032990~cell part morphogenesis	9.08E-06	7.52E-07	1	2.98E-08	8.80E-07	1.69E-05	1	1	1	1
	GO:0048812~neuron projection morphogenesis	9.10E-06	9.46E-07	1	9.44E-06	4.51E-06	1.73E-05	1	1	1	1
	GO:0030182~neuron differentiation	5.65E-09	3.20E-10	1.47E-05	2.50E-07	6.28E-06	1.08E-05	1	1	1	1
	GO:0042127~regulation of cell proliferation	3.69E-08	1	4.42E-08	7.52E-07	1	1	1	2.63E-05	1.04E-06	1
	GO:0007389~pattern specification process	9.08E-07	4.67E-07	4.06E-09	8.40E-07	9.26E-06	7.40E-05	1	1	1	1
	GO:0030030~cell projection organization	4.58E-08	6.23E-07	1.70E-06	2.14E-09	5.75E-07	1.46E-07	4.10E-05	1	1	1
	GO:0006357~regulation of transcription from RNA polymerase II promoter	1	1	6.20E-06	2.81E-06	1	1.53E-08	5.79E-12	8.40E-12	2.40E-12	3.01E-13
transcription	GO:0006350~transcription	1	1	7.06E-05	2.94E-07	1.41E-06	3.89E-12	9.09E-10	1.63E-11	3.06E-14	3.10E-13
	GO:0045449~regulation of transcription	1	1	1	3.92E-07	9.39E-06	1.09E-10	1.89E-10	1.53E-11	2.54E-14	5.64E-12
	GO:0006468~protein amino acid phosphorylation	1	1.28E-05	3.80E-05	1.59E-08	6.48E-07	7.12E-06	5.83E-11	6.86E-11	2.22E-09	3.60E-10
phosphate	GO:0006796~phosphate metabolic process	1	1	1	3.49E-07	4.59E-06	8.32E-05	8.77E-09	6.87E-12	1.50E-09	9.99E-10
	GO:0006793~phosphorus metabolic process	1	1	1	3.49E-07	4.59E-06	8.32E-05	8.77E-09	6.87E-12	1.50E-09	9.99E-10
	GO:0043067~regulation of programmed cell death	1	9.95E-05	1	1	1	2.65E-05	2.38E-08	7.94E-11	3.34E-08	2.02E-06
cell death	GO:0010941~regulation of cell death	1	1	1	1	1	2.27E-05	3.16E-08	1.13E-10	4.45E-08	2.35E-06
	GO:0042981~regulation of apoptosis	1	8.69E-05	1	1	1	1.94E-05	4.38E-08	9.52E-11	4.02E-08	1.61E-06
	GO:0010605~negative regulation of macromolecule metabolic process	1	1	7.38E-08	1	1	3.05E-08	2.19E-10	3.19E-10	4.75E-13	3.14E-10
	GO:0030163~protein catabolic process	1	1	1	1	1	1	7.81E-05	1.38E-09	5.36E-12	6.19E-16
	GO:0051603~proteolysis involved in cellular protein catabolic process	1	1	1	1	1	1	3.60E-05	8.92E-10	5.46E-12	6.43E-16
metabolic	GO:0019941~modification-dependent protein catabolic process	1	1	1	1	1	1	6.10E-05	7.94E-10	8.01E-12	6.66E-16
	GO:0044257~cellular protein catabolic process	1	1	1	1	1	1	6.45E-05	7.83E-10	8.61E-12	4.41E-16
	GO:0043632~modification-dependent macromolecule catabolic process	1	1	1	1	1	1	6.10E-05	7.94E-10	8.01E-12	6.66E-16
	GO:0044265~cellular macromolecule catabolic process	1	1	1	1	1	1	1	1.24E-08	4.35E-09	9.33E-13
	GO:0009057~macromolecule catabolic process	1	1	1	1	1	1	1	2.00E-08	1.68E-08	2.39E-12
	GO:0044093~positive regulation of molecular function	1	1	1.39E-05	1	1	3.81E-07	4.91E-07	7.39E-08	1.14E-07	1
	GO:0051094~positive regulation of developmental process	2.56E-10	1.18E-09	5.73E-08	1	1	1	5.76E-06	7.49E-05	1	1.21E-05
	GO:0009891~positive regulation of biosynthetic process	2.41E-06	7.21E-06	5.72E-07	5.60E-07	1	8.12E-07	3.77E-11	6.94E-09	3.38E-10	4.17E-08
	GO:0010557~positive regulation of macromolecule biosynthetic process	5.24E-06	5.52E-05	2.88E-06	4.03E-06	1	5.60E-07	1.28E-10	8.08E-09	6.29E-10	1.57E-08
regulation	GO:0007242~intracellular signaling cascade	2.60E-06	1.78E-08	4.05E-08	3.24E-08	3.06E-07	4.10E-09	5.53E-12	3.38E-10	4.22E-10	8.66E-08
	GO:0010604~positive regulation of macromolecule metabolic process	4.77E-07	1.45E-06	1.92E-08	2.95E-07	3.93E-06	1.37E-07	5.13E-15	1.15E-11	1.43E-11	1.68E-09
	GO:0051173~positive regulation of nitrogen compound metabolic process	1.37E-05	3.45E-05	1.82E-07	1.54E-07	1.53E-06	1.51E-07	2.92E-12	5.53E-09	4.15E-10	1.08E-07
	GO:0031328~positive regulation of cellular biosynthetic process	4.41E-06	8.86E-06	6.09E-07	7.97E-07	7.29E-05	7.82E-07	1.45E-11	4.84E-09	2.25E-10	7.41E-08
	GO:0045935~positive regulation of nucleobase, nucleoside, nucleotide and nucleic acid me	2.18E-05	7.24E-05	4.68E-07	3.21E-07	9.34E-06	2.05E-07	3.50E-11	1.10E-08	1.41E-09	9.22E-08

Table S4. KEGG term enrichments at varying complexity category, Related to Figure S2

		KEGG term \ kind	TC0	TC1	TC2	TC3	TC4	TC5	TC6	TC7	TC8	TC9
Environmental Information Processing	Signaling molecules and interaction	hsa04080:Neuroactive ligand-receptor interaction	2.78E-07	9.82E-06	1	1	1	1	1	1	1	1
Environmental Information Processing	Signaling molecules and interaction	hsa04060:Cytokine-cytokine receptor interaction	1.01E-05	1	1	1	1	1	1	1	1	1
Environmental Information Processing	Signal transduction	hsa04350:TGF-beta signaling pathway	1	1.91E-04	1	1	1	1	1	1	1	1
Environmental Information Processing	Signal transduction	hsa04020:Calcium signaling pathway	1	8.42E-04	1.79E-04	1	2.92E-04	1	1	1	1	1
Environmental Information Processing	Signaling molecules and interaction	hsa04512:ECM-receptor interaction	1	1	1	1	2.30E-04	1	1	1	1	1
Human Diseases	Cancers: Specific types	hsa05219:Bladder cancer	1	1	1	8.92E-04	1	1	1	1	1	1
Organismal Systems	Circulatory system	hsa04270:Vascular smooth muscle contraction	1	2.82E-04	1	1	1	1	5.57E-06	1	1	1
Organismal Systems	Endocrine system	hsa04916:Melanogenesis	1	1	1	1	1.10E-05	5.98E-05	7.94E-04	1	1	1
Organismal Systems	Endocrine system	hsa04912:GnRH signaling pathway	1	1	1	1	1	1	6.44E-06	1	1	1
Cellular Processes	Cell motility	hsa04810:Regulation of actin cytoskeleton	1	1	1	1	1	6.16E-04	5.48E-04	1	1	1
Cellular Processes	Cell communication	hsa04540:Gap junction	1	1	1	1	9.51E-04	1	2.30E-05	1	4.89E-04	1
Cellular Processes	Cell communication	hsa04520:Adherens junction	1	1	1	1	1	1	1	8.62E-04	1	1.11E-04
Cellular Processes	Transport and catabolism	hsa04144:Endocytosis	1	1	1	1	1	1	3.11E-04	6.23E-04	5.53E-08	9.01E-05
Organismal Systems	Nervous system	hsa04722:Neurotrophin signaling pathway	1	1	1	1	1	1	2.02E-05	1.19E-06	3.66E-07	8.73E-07
Organismal Systems	Endocrine system	hsa04910:Insulin signaling pathway	1	1	1	1	1	1	1	5.61E-04	2.84E-05	8.71E-06
Organismal Systems	Immune system	hsa04666:Fc gamma R-mediated phagocytosis	1	1	1	1	1	1	1	1	1	4.13E-06
Genetic Information Processing	Folding, sorting and degradation	hsa04120:Ubiquitin mediated proteolysis	1	1	1	1	1	1	1	1	1	1.73E-06
Environmental Information Processing	Signal transduction	hsa04310:Wnt signaling pathway	1	1	1	1	2.26E-04	1	1	1	6.85E-04	1
Environmental Information Processing	Signal transduction	hsa04012:ErbB signaling pathway	1	1	1	1	1.79E-04	1	4.69E-05	9.98E-04	1	5.58E-05
Environmental Information Processing	Signal transduction	hsa04010:MAPK signaling pathway	1	1	1	6.85E-04	1	1	7.28E-07	7.80E-04	1.87E-05	2.96E-05
Cellular Processes	Cell communication	hsa04510:Focal adhesion	1	1	1	2.56E-04	3.41E-04	8.58E-04	4.41E-07	2.14E-04	5.47E-05	9.08E-06
Human Diseases	Cancers: Specific types	hsa05220:Chronic myeloid leukemia	1	1	1	1	4.94E-04	1	4.57E-04	1	3.60E-05	4.06E-05
Human Diseases	Cancers: Specific types	hsa05214:Glioma	1	1	1	1	1	1	2.52E-06	1	4.44E-05	1.95E-04
Human Diseases	Cancers: Specific types	hsa05210:Colorectal cancer	1	1	1	1	1	1	6.70E-06	2.17E-04	3.45E-06	1.56E-06
Human Diseases	Cancers: Specific types	hsa05215:Prostate cancer	1	1	1	1	1	8.92E-04	2.89E-06	1.02E-04	4.58E-06	2.35E-05
Human Diseases	Cancers: Overview	hsa05200:Pathways in cancer	1	1	2.20E-04	1.85E-05	2.39E-05	7.36E-06	5.04E-10	3.69E-05	6.95E-09	2.20E-07

Table S5. GSC statistical analysis of TFBS-clustered regions with TF peaks, Related to Figure 3 and Figure S3

		LOW				MID				HIG			
TF names	overlap percent	real stat	expected mean	bootstrap SD	Z Score	real stat	expected mean	bootstrap SD	Z Score	real stat	expected mean	bootstrap SD	Z Score
E2f6	84.6%	0.024011162	0.076950676	0.001342015	-39.44779434	0.065782972	0.076950676	0.001285011	-8.690748182	0.156423726	0.076950676	0.001552837	51.17927475
Cmyc	62.6%	0.046218583	0.094558446	0.001679662	-28.77951255	0.085826558	0.094558446	0.00162542	-5.372080587	0.170871884	0.094558446	0.001883505	40.5167096
Ctcf	74.0%	0.233108168	0.168377737	0.001894739	34.1632527	0.167925364	0.168377737	0.001844868	-0.245206114	0.106507978	0.168377737	0.002103975	-29.40612787
Rad21	69.6%	0.237129941	0.170441966	0.002247273	29.67506724	0.171454314	0.170441966	0.00217771	0.464868057	0.105640945	0.170441966	0.002383016	-27.19285755
Pou5f1	84.5%	0.008677328	0.011072834	0.000473227	-5.06206348	0.014091047	0.011072834	0.000435776	6.926064842	0.011116286	0.011072834	0.000517726	0.083929968
Nanog	86.0%	0.013442213	0.015299131	0.000691056	-2.687072146	0.019198144	0.015299131	0.000653571	5.965709346	0.014362058	0.015299131	0.000707418	-1.324640407
K562Nelfe	94.0%	0.000279951	0.001772127	0.000227977	-6.545299968	0.001364843	0.001772127	0.000207806	-1.959925132	0.004063243	0.001772127	0.000242509	9.447563939
Mafk	31.2%	0.033058966	0.04177964	0.001052231	-8.287798509	0.046823378	0.04177964	0.000997656	5.055589052	0.050497068	0.04177964	0.001104559	7.892227776
K562Brf1	91.9%	5.74E-05	3.22E-04	8.83E-05	-3.00E+00	0.000300567	0.000322086	8.33E-05	-2.58E-01	0.000641935	0.000322086	0.000100447	3.184263923
K562Znf274Ucd	6.3%	0.000128769	0.000155185	0.000100847	-0.261943281	4.37E-05	1.55E-04	8.99E-05	-1.24E+00	0.000373675	0.000155185	9.86E-05	2.22E+00
Atf2sc81188	92.7%	0.02781203	0.053424845	0.001674646	-15.29446908	0.053164631	0.053424845	0.001649323	-0.157770037	0.089154128	0.053424845	0.001827053	19.55569503
Atf3	89.5%	0.003464479	0.009317593	0.000347821	-16.82793621	0.007816216	0.009317593	0.000326487	-4.598577687	0.021547522	0.009317593	0.000433408	28.21804136
Creb1	93.0%	0.018298554	0.065009492	0.001573231	-29.69108933	0.053560711	0.065009492	0.001535973	-7.453762703	0.148067318	0.065009492	0.001843352	45.05802998
Egr1	88.8%	0.003886918	0.01129663	0.000424638	-17.44947642	0.009902518	0.01129663	0.000393394	-3.5438081	0.023366892	0.01129663	0.000500591	24.11203984
Fosl1sc183	84.3%	0.00169466	0.005194077	0.000260555	-13.4306366	0.003905056	0.005194077	0.000239343	-5.385662865	0.011888347	0.005194077	0.000319011	20.9844769
Gabp	91.2%	0.040511047	0.074884399	0.002504314	-13.72565719	0.065627433	0.074884399	0.002505471	-3.694700478	0.127089514	0.074884399	0.00272976	19.12443521
Hdac2sc6296	90.8%	0.006003518	0.009656258	0.000381645	-9.571041984	0.009789315	0.009656258	0.000351126	0.378944183	0.014542448	0.009656258	0.00042703	11.44226364
Jund	90.8%	0.006740811	0.015031856	0.000511659	-16.2042396	0.015659904	0.015031856	0.00048436	1.296655445	0.025936257	0.015031856	0.000596238	18.2886712
Max	88.6%	0.054588789	0.157222785	0.002132436	-48.12992249	0.131274109	0.157222785	0.002057361	-12.61260236	0.319344454	0.157222785	0.002437935	66.49959799
Nrsf	38.8%	0.017708836	0.018310091	0.000547779	-1.097624823	0.018485944	0.018310091	0.000511782	0.343608153	0.018028322	0.018310091	0.000636704	-0.442543074
P300	91.8%	0.005263814	0.010918788	0.00042035	-13.45300214	0.010627923	0.010918788	0.000388897	-0.74792279	0.019911727	0.010918788	0.000499121	18.0175358
Pol2	58.8%	0.053304429	0.166734855	0.003860524	-29.38212857	0.136177559	0.166734855	0.003744245	-8.161137015	0.33651143	0.166734855	0.004168598	40.72750113
Rxra	88.0%	0.001400175	0.002428269	0.000177565	-5.78994851	0.002738118	0.002428269	0.000164098	1.888186798	0.003740422	0.002428269	0.000214634	6.113438067
Sin3ak20	90.3%	0.021291625	0.074336354	0.002055487	-25.80640958	0.06022545	0.074336354	0.00202539	-6.967005509	0.158097623	0.074336354	0.002286157	36.63846186
Six5	85.6%	0.005649255	0.011191466	0.000484063	-11.44936434	0.010987573	0.011191466	0.00048785	-0.417942389	0.019950285	0.011191466	0.000665929	13.15278162
Sp1	92.9%	0.038626743	0.088136202	0.002050095	-24.14983122	0.084726242	0.088136202	0.002044246	-1.668077179	0.161493758	0.088136202	0.002402754	30.53061176
Sp2	92.3%	0.004898909	0.011319855	0.00046668	-13.75877432	0.01144198	0.011319855	0.000450564	0.271048655	0.020132495	0.011319855	0.000568786	15.49376021
Sp4v20	94.7%	0.006349633	0.034946702	0.001303085	-21.94566593	0.028273002	0.034946702	0.001302262	-5.12469744	0.083252323	0.034946702	0.001597435	30.23948834
Srf	58.8%	0.005576423	0.00938614	0.00035544	-10.71832518	0.009505178	0.00938614	0.000326886	0.364158263	0.015435299	0.00938614	0.00042492	14.23600814
Taf1	88.5%	0.03432254	0.129922141	0.00299087	-31.9638099	0.107441324	0.129922141	0.002989147	-7.520813968	0.279377802	0.129922141	0.003253313	45.93952494
Taf7sc101167	94.0%	0.004746428	0.022861099	0.000836979	-21.64292969	0.019390709	0.022861099	0.000797064	-4.353965129	0.05294951	0.022861099	0.000945589	31.81975813
Tcf12	86.1%	0.031635677	0.044974221	0.001064601	-12.52914433	0.049293948	0.044974221	0.001003	4.306804924	0.057573116	0.044974221	0.001162932	10.8337316
Tead4sc101184	78.7%	0.102941099	0.110202516	0.002266203	-3.204221969	0.117510065	0.110202516	0.002184645	3.34495931	0.118998459	0.110202516	0.002308177	3.810775771
Usf1	53.6%	0.029051078	0.047452063	0.000819252	-22.46070954	0.045484644	0.047452063	0.000746011	-2.637251924	0.080560802	0.047452063	0.000967268	34.22914272
Yy1sc281	78.9%	0.076024996	0.110994996	0.001969511	-17.75567545	0.106311811	0.110994996	0.001901899	-2.462373167	0.168597444	0.110994996	0.00211011	27.29831013
Bach1sc14700	67.0%	0.051486388	0.121922849	0.001691529	-41.64069319	0.108289554	0.121922849	0.001648719	-8.269019957	0.225910893	0.121922849	0.0019353	53.73226441
Brca1	77.8%	0.022393574	0.036817195	0.000957079	-15.07046401	0.034302249	0.036817195	0.000920433	-2.732350434	0.060020894	0.036817195	0.001081778	21.44960145
Cebpb	36.8%	0.067346088	0.082685552	0.001512365	-10.14270187	0.088149287	0.082685552	0.001448532	3.771912299	0.099843324	0.082685552	0.001596699	10.74577682
Chd1a301218a	70.4%	0.013510555	0.051961615	0.001566455	-24.54654171	0.042238504	0.051961615	0.001532025	-6.346574669	0.101823294	0.051961615	0.001668075	29.89175264
Chd2	83.7%	0.0667148	0.134961968	0.002333301	-29.24919077	0.126327926	0.134961968	0.002255774	-3.827529155	0.235110449	0.134961968	0.002527722	39.62005865
Cjun	46.9%	0.023193638	0.035764558	0.000782356	-16.06802449	0.038650166	0.035764558	0.000731948	3.942368249	0.050435876	0.035764558	0.000878075	16.70850567
Ctbp2	95.2%	0.028284186	0.107923786	0.002672228	-29.80269293	0.088583787	0.107923786	0.002563986	-7.542942063	0.211357527	0.107923786	0.002778207	37.23039266
Gtf2f1	75.5%	0.095779928	0.127896296	0.00225106	-14.26722259	0.122854251	0.127896296	0.002188319	-2.304072155	0.181611807	0.127896296	0.002433111	22.07688585
Mxi1	79.0%	0.052459053	0.111030819	0.001970279	-29.72765604	0.100144344	0.111030819	0.001929824	-5.641174541	0.198600327	0.111030819	0.002167082	40.40895532
Nrf1	59.8%	0.009506575	0.028726156	0.000768707	-25.00247887	0.025693078	0.028726156	0.000735753	-4.122411413	0.058563328	0.028726156	0.000895703	33.31145668
Rfx5200401194	64.8%	0.011713632	0.018415665	0.000646994	-10.35872023	0.018027357	0.018415665	0.000622082	-0.624206949	0.027502785	0.018415665	0.00075748	11.99652301
Sin3anb6001263	86.3%	0.112308663	0.258365843	0.003653759	-39.97449714	0.219029175	0.258365843	0.00363522	-10.82098822	0.470938023	0.258365843	0.004057079	52.39537377
Tbp	86.1%	0.066109119	0.163163483	0.002785774	-34.83928082	0.141935409	0.163163483	0.002751283	-7.715699268	0.307066664	0.163163483	0.003058976	47.04292317
Usf2	65.2%	0.03513782	0.067339485	0.001186824	-27.13264508	0.061266577	0.067339485	0.001149458	-5.283277615	0.120942487	0.067339485	0.001358769	39.44967425
Znf143	67.4%	0.219803137	0.234766471	0.002662322	-5.620408086	0.224585435	0.234766471	0.002555465	-3.984025477	0.2785756	0.234766471	0.002875261	15.23657649
Gm12878Bhlhe40c	77.5%	0.197788163	0.333132568	0.002501527	-54.10470588	0.312784465	0.333132568	0.002260794	-9.000421837	0.475192993	0.333132568	0.002516961	56.44124972
Gm12878Elk112771	73.4%	0.051191767	0.130007613	0.001962773	-40.15536425	0.108512142	0.130007613	0.001781152	-12.0682998	0.222248188	0.130007613	0.002027965	45.48429656
Gm12878Irf3	42.5%	0.015972405	0.028166626	0.00096368	-12.65380899	0.025625206	0.028166626	0.000906379	-2.803927138	0.040557961	0.028166626	0.00102847	12.04832215
Gm12878Whip	57.4%	0.209917738	0.284759173	0.002930854	-25.53571097	0.269374243	0.284759173	0.002725932	-5.643916285	0.339805301	0.284759173	0.002946814	18.67987845
Helas3Ap2alpha	96.9%	0.027435609	0.058483459	0.00140394	-22.11480555	0.054664632	0.058483459	0.001277291	-2.989786456	0.095919435	0.058483459	0.001469972	25.46713276
Helas3Ap2gamma	96.5%	0.037914024	0.083085306	0.00180813	-24.98232491	0.076179862	0.083085306	0.001677324	-4.11693995	0.139641504	0.083085306	0.0018492	30.58414575
Helas3Baf155	98.5%	0.013714459	0.062104404	0.001940555	-24.93613235	0.053969029	0.062104404	0.00183702	-4.428571486	0.123833785	0.062104404	0.002000463	30.85754596
Helas3Baf170	97.7%	0.002177806	0.01346001	0.000835472	-13.5039854	0.011366626	0.01346001	0.000780263	-2.68292163	0.028156954	0.01346001	0.000845746	17.37749817
Helas3E2f1	97.1%	0.002194404	0.028107536	0.000847584	-30.57293029	0.014556527	0.028107536	0.000747361	-18.13180757	0.075410088	0.028107536	0.000910827	51.93365526
Helas3Elk4Ucd	92.3%	0.011952588	0.051508908	0.001330524	-29.72988013	0.041680112	0.051508908	0.001205283	-8.154760937	0.106894428	0.051508908	0.001412941	39.19875354
Helas3Tcf7l2Ucd	90.9%	0.006629319	0.015345241	0.000566309	-15.39074766	0.014974777	0.015345241	0.000501453	-0.738781553	0.024767436	0.015345241	0.000605728	15.55514984
Helas3Zkscan1hpa006672	75.6%	0.048255795	0.121597829	0.002393211	-30.64587155	0.099784179	0.121597829	0.002228191	-9.789847983	0.229851325	0.121597829	0.002411339	44.89351971
Hepg2ErraForskln	93.7%	0.000667196	0.001923004	0.000197393	-6.361985372	0.001777753	0.001923004	0.000173013	-0.839538754	0.003589947	0.001923004	0.000219146	7.60653825
Hepg2Hnf4aForskln	96.5%	0.020943494	0.042669739	0.000907118	-23.95083833	0.043505352	0.042669739	0.000796409	1.049226105	0.065989686	0.042669739	0.001021629	22.82623284
Hepg2Hsf1Forskln	92.9%	0.001326515	0.003347911	0.000451936	-4.472745339	0.003239315	0.003347911	0.00042214	-0.257250706	0.005824716	0.003347911	0.000458591	5.400906715
Hepg2Pgc1aForskln	94.3%	6.68E-05	3.63E-04	9.10E-05	-3.25E+00	0.000275878	0.00036281	8.02E-05	-1.08E+00	0.000842598	0.00036281	1.00E-04	4.80E+00
Hepg2Srebp1Insln	95.8%	0.000964478	0.004786998	0.000320778	-11.91640455	0.003861377	0.004786998	0.000281114	-3.292689046	0.010657872	0.004786998	0.000365102	16.0801019
Hepg2Srebp1Pravast	92.1%	0.000237129	0.000593046	9.95E-05	-3.58E+00	0.000524709	0.000593046	8.59E-05	-7.95E-01	0.001110269	0.000593046	0.000112249	4.607809173
Hepg2Srebp2Pravast	94.2%	0.000233025	0.000531602	9.11E-05	-3.28E+00	0.000416862	0.000531602	7.75E-05	-1.48E+00	0.001060128	0.000531602	0.000104536	5.055934756
K562Arid3asc8821	75.9%	0.107044038	0.14530107	0.002394186	-15.97914288	0.150449053	0.14530107	0.002225246	2.313443734	0.177428414	0.14530107	0.002401216	13.37961267
K562Atf106325	65.4%	0.088040039	0.130315138	0.001884574	-22.43218245	0.133370259	0.130315138	0.001743071	1.752723548	0.170312371	0.130315138	0.001966155	20.34286752
K562Bhlhe40nb100	69.3%	0.148671201	0.24108802	0.002171343	-42.56206106	0.222298338	0.24108802	0.001987102	-9.455823567	0.372001327	0.24108802	0.002337675	56.00150407
K562Brg1	98.1%	0.002139921	0.005260698	0.000396794	-7.864981236	0.00548555	0.005260698	0.000350963	0.640672341	0.00821448	0.005260698	0.00043485	6.792651957
K562Ccnt2	77.7%	0.080260553	0.19093231	0.002206348	-50.16060328	0.165582802	0.19093231	0.002011258	-12.60380807	0.352566972	0.19093231	0.002319032	69.69918493
K562Cfos	93.5%	0.009062847	0.021432819	0.000650732	-19.00931661	0.020660328	0.021432819	0.000580171	-1.331488603	0.035980982	0.021432819	0.000710179	20.48521535
K562Corestab24166	73.6%	0.096032321	0.164930486	0.002005693	-34.35130308	0.155149857	0.164930486	0.001826847	-5.353831501	0.255312016	0.164930486	0.002112827	42.77752952
K562E2f4Ucd	97.7%	0.006061533	0.054293041	0.001347105	-35.80383029	0.034552844	0.054293041	0.001220177	-16.17813628	0.139573488	0.054293041	0.001489799	57.24293642
K562Gata1Ucd	92.5%	0.004678398	0.007493544	0.000352467	-7.986983817	0.00825757	0.007493544	0.000294422	2.594999785	0.009200061	0.007493544	0.000379692	4.494471964
K562Gtf2b	88.9%	0.010359289	0.039925502	0.001026805	-28.79438872	0.032871644	0.039925502	0.000953075	-7.401155181	0.083587414	0.039925502	0.001086066	40.20190909
K562Hmgn3	75.0%	0.055756549	0.160846858	0.002373403	-44.27833168	0.12967793	0.160846858	0.002242193	-13.90109265	0.326446342	0.160846858	0.002578303	64.228087
K562Ini1	98.0%	0.001348266	0.004006473	0.000341256	-7.789469203	0.004069186	0.004006473	0.000300031	0.209022355	0.006742324	0.004006473	0.000387585	7.058705151
K562Maff	38.9%	0.063190177	0.090698119	0.00137951	-19.94037334	0.097029597	0.090698119	0.001259558	5.026746538	0.109309196	0.090698119	0.001448308	12.85021751
K562Mazab85725	82.6%	0.207461224	0.317448342	0.003222706	-34.12880409	0.282830595	0.317448342	0.003012651	-11.49079122	0.494172215	0.317448342	0.003292314	53.67770698
K562Nfe2	94.3%	0.001427836	0.005079546	0.000266793	-13.68744305	0.004472873	0.005079546	0.000217719	-2.786489706	0.010020613	0.005079546	0.000286001	17.27640393
K562Znf263Ucd	75.7%	0.003182157	0.010883381	0.000543166	-14.17839102	0.008029065	0.010883381	0.000485475	-5.879424722	0.023992192	0.010883381	0.00061298	21.38537581
K562Nfya	69.6%	0.014501279	0.028079556	0.000822286	-16.51284487	0.02602655	0.028079556	0.000743331	-2.761899141	0.046105896	0.028079556	0.000900788	20.01174639
K562Nfyb	48.8%	0.022628663	0.041825645	0.000985889	-19.47175643	0.038014293	0.041825645	0.000898805	-4.240467985	0.068862693	0.041825645	0.001083868	24.94496035
K562Rfx5	32.4%	0.021679661	0.042281457	0.001107289	-18.60562619	0.038546784	0.042281457	0.001056233	-3.535840992	0.070690033	0.042281457	0.00120291	23.6165343
K562Setdb1Ucd	63.2%	0.00332055	0.011763445	0.000594338	-14.20554976	0.009074497	0.011763445	0.00053166	-5.057648976	0.025383171	0.011763445	0.000645344	21.10460494
K562Tblr1ab24550	79.4%	0.058134178	0.126908832	0.001976048	-34.80414307	0.110754186	0.126908832	0.001925771	-8.388662862	0.228039084	0.126908832	0.002160877	46.80055739
K562Tf3c110	87.1%	0.00326985	0.009123841	0.000970162	-6.034033275	0.008355993	0.009123841	0.000970521	-0.791171318	0.016695348	0.009123841	0.001010236	7.494793996
K562Ubfsc13125	77.6%	0.04445284	0.117214613	0.001733871	-41.96493721	0.095292021	0.117214613	0.001580742	-13.86854803	0.232455239	0.117214613	0.00185502	62.12365771
K562Ubtfsab1404509	82.3%	0.099282799	0.200191399	0.002327262	-43.35936182	0.170715142	0.200191399	0.002177555	-13.53640113	0.35842913	0.200191399	0.002507191	63.11354794
Bcl11aPcr1x	90.4%	0.005697061	0.007198004	0.000407044	-3.687424435	0.009028808	0.007198004	0.000382271	4.78928637	0.007682504	0.007198004	0.000432463	1.120327938
K562Gata2Ucd	93.5%	0.01894451	0.027663597	0.000689434	-12.64673295	0.030751365	0.027663597	0.000584988	5.278341274	0.031717513	0.027663597	0.00075499	5.369496503
K562Rpc155	78.6%	0.002170166	0.003170602	0.000567958	-1.761460436	0.003208724	0.003170602	0.000571989	0.066648223	0.004175474	0.003170602	0.000572432	1.755445995
Gm12878Ebf1sc137065	59.5%	0.28406682	0.365101906	0.003178093	-25.49801874	0.369742081	0.365101906	0.002996334	1.54861723	0.431760066	0.365101906	0.003260784	20.44237238
Gm12878NfkbTnfa	91.0%	0.02956686	0.055408193	0.001623053	-15.92144016	0.059488413	0.055408193	0.001480196	2.756540663	0.071380609	0.055408193	0.001618358	9.869521862
Gm12878Stat3	73.3%	0.074149221	0.10956045	0.002146384	-16.49808934	0.114688503	0.10956045	0.001988252	2.579177201	0.135989269	0.10956045	0.00209945	12.58845028
Helas3Prdm19115	63.3%	0.017925622	0.031913567	0.000930199	-15.03758327	0.034143294	0.031913567	0.000861489	2.588225721	0.042659006	0.031913567	0.00097427	11.02921904
K562Smc3ab9263	80.7%	0.157512086	0.125255129	0.001411948	22.84570472	0.11451059	0.125255129	0.001232894	-8.714888678	0.10909841	0.125255129	0.001493921	-10.81497683
K562Tr4Ucd	92.5%	0.00034444	0.001579638	0.000174283	-7.087321837	0.001217509	0.001579638	0.000153409	-2.360546209	0.003518836	0.001579638	0.00018677	10.38280078
Hepg2Grp20Forskln	94.9%	0.00015289	0.000925302	0.000113818	-6.786377648	0.00069537	0.000925302	9.89E-05	-2.32E+00	0.002182319	0.000925302	0.000132285	9.502320661
Gm12878Stat1	79.1%	0.128668445	0.215931578	0.002260825	-38.59792135	0.191935772	0.215931578	0.002070564	-11.58901826	0.317149353	0.215931578	0.002345247	43.15867603
K562Brf2	89.5%	6.32E-05	1.21E-04	5.61E-05	-1.04E+00	5.67E-05	1.21E-04	5.00E-05	-1.29E+00	0.000293968	0.000121362	6.35E-05	2.72E+00
K562Bdp1	90.6%	0.000911134	0.001649055	0.000459045	-1.607514249	0.001653351	0.001649055	0.000454649	0.009449957	0.002430915	0.001649055	0.000459867	1.700189085
K562Pol3	88.9%	0.000127228	0.000401649	0.000152536	-1.799055837	0.00030871	0.000401649	0.000146995	-0.632260929	0.00085379	0.000401649	0.000156524	2.88863483
K562Xrcc4	93.3%	1.81E-05	1.71E-04	9.37E-05	-1.63E+00	0.00011484	0.000170711	8.45E-05	-6.62E-01	0.000429279	0.000170711	9.47E-05	2.73E+00
Gm12878Spt20	10.4%	0.003087777	0.005910207	0.000469563	-6.010758428	0.004973127	0.005910207	0.000426637	-2.19643523	0.009265252	0.005910207	0.000462875	7.248275959
Gm12878Zzz3	60.3%	0.001684039	0.002997976	0.000368975	-3.561050533	0.002711058	0.002997976	0.000328067	-0.874570144	0.004002093	0.002997976	0.000361853	2.774934332

Table S6. GSC statistical analysis of TFBS-clustered regions with Histone peaks, Related to Figure 4 and Figure S4

Histone name		LOW				MID				HIG			
	overlap percent	real stat	expected mean	bootstrap SD	Z Score	real stat	expected mean	bootstrap SD	Z Score	real stat	expected mean	bootstrap SD	Z Score
H2az	34.3%	0.41787126	0.456907616	0.007496941	-5.206971383	0.45398819	0.456907616	0.007356055	-0.396873881	0.511690043	0.456907616	0.007905967	6.929251026
H3k4me1	55.5%	0.438704779	0.52898703	0.004794031	-18.83222227	0.522850681	0.52898703	0.004674022	-1.312862757	0.607193199	0.52898703	0.005009784	15.61068675
H3k4me2	77.3%	0.231267596	0.443531138	0.004601942	-46.12477405	0.403422262	0.443531138	0.004524755	-8.86431975	0.704164271	0.443531138	0.005030704	51.80848499
H3k4me3	88.6%	0.105727233	0.30424457	0.004586149	-43.28628333	0.249773477	0.30424457	0.004571403	-11.91561927	0.564453134	0.30424457	0.004887216	53.24269981
H3k09me3	10.4%	0.139583602	0.122881973	0.004879348	3.42292227	0.12911382	0.122881973	0.00473468	1.31621305	0.098165446	0.122881973	0.00496232	-4.980840544
H3k9ac	66.5%	0.326023811	0.455281865	0.006937309	-18.63230318	0.421273975	0.455281865	0.006690042	-5.083359736	0.623760202	0.455281865	0.006800607	24.77401555
H3k27ac	68.7%	0.386009938	0.459162968	0.005963728	-12.26632671	0.447921186	0.459162968	0.005909768	-1.902237452	0.55605305	0.459162968	0.006096807	15.8919379
H3k27me3	59.9%	0.187450121	0.267974422	0.00681745	-11.81149885	0.247353338	0.267974422	0.006591036	-3.128655808	0.347745891	0.267974422	0.006762895	11.79546118
H3k36me3	27.1%	0.237901571	0.242237179	0.005916441	-0.732806666	0.237896732	0.242237179	0.005675141	-0.764817371	0.250585785	0.242237179	0.005864649	1.423547478
H3k79me2	37.8%	0.167241073	0.214573621	0.003248039	-14.57265193	0.196068972	0.214573621	0.003032199	-6.102716334	0.28354975	0.214573621	0.003500734	19.70333112
H4k20me1	44.1%	0.374600602	0.45560637	0.007217457	-11.22358828	0.420893489	0.45560637	0.007112387	-4.8806231	0.556367994	0.45560637	0.007475324	13.47923196

Table S7. Percentage of the number of TFBS-clustered regions in each category

Cell type \ kind	TC0	TC1	TC2	TC3	TC4	TC5	TC6	TC7	TC8	TC9
Uw_HFF	19.0%	12.4%	12.4%	11.2%	9.4%	7.6%	8.7%	7.8%	6.9%	4.6%
Uw_SKMC	19.1%	12.3%	12.5%	11.4%	9.7%	7.7%	8.7%	7.8%	6.7%	4.1%
Duke_T47D	20.1%	12.0%	11.2%	9.9%	8.4%	7.0%	8.0%	7.9%	8.0%	7.6%
UWDuke_LNCaP	17.7%	11.4%	11.4%	10.4%	9.1%	7.5%	8.8%	8.2%	8.4%	7.2%
Duke_H9ES	19.3%	12.0%	11.5%	10.4%	8.7%	7.2%	8.2%	7.9%	8.2%	6.6%
Uw_HMVECdNeo	14.7%	10.8%	11.1%	10.5%	9.2%	8.1%	9.4%	9.3%	9.6%	7.4%
Duke_GM12891	17.5%	10.9%	10.4%	9.7%	8.4%	7.1%	8.8%	9.0%	9.8%	8.4%
Uw_BE2C	19.6%	12.4%	12.1%	10.9%	9.3%	7.5%	8.4%	7.6%	7.1%	5.1%
Uw_HBMEC	18.4%	12.3%	12.5%	11.5%	9.8%	7.9%	8.9%	7.9%	6.9%	4.0%
Uw_NB4	16.2%	10.7%	11.0%	10.1%	8.7%	7.3%	8.9%	9.6%	10.0%	7.5%
Uw_BJ	18.4%	12.7%	12.7%	11.5%	9.8%	7.8%	8.6%	7.7%	6.8%	4.1%
Uw_AG10803	19.4%	12.5%	12.5%	11.2%	9.4%	7.5%	8.6%	7.5%	6.9%	4.5%
Uw_AG09319	18.3%	12.1%	12.1%	11.1%	9.4%	7.7%	8.8%	8.0%	7.4%	5.1%
Duke_8988T	20.1%	12.1%	11.7%	10.0%	8.6%	7.3%	8.2%	7.8%	7.7%	6.6%
Tregwb78495824_UW	13.7%	10.0%	10.0%	9.6%	8.6%	7.5%	9.7%	10.5%	11.2%	9.2%
Duke_PanIslets	18.1%	11.2%	11.1%	10.2%	9.0%	7.4%	8.7%	8.2%	8.5%	7.6%
Uw_AG04450	18.4%	12.1%	12.0%	11.1%	9.3%	7.8%	8.9%	8.1%	7.3%	5.0%
UWDuke_H1hESC	19.0%	11.6%	11.2%	10.2%	8.7%	7.3%	8.5%	8.1%	8.4%	7.2%
Uw_WI38	18.0%	11.9%	12.0%	11.1%	9.6%	7.8%	9.0%	8.4%	7.4%	4.8%
Uw_SAEC	18.6%	12.0%	12.0%	11.0%	9.5%	7.8%	8.8%	8.2%	7.6%	4.6%
Uw_PrEC	19.3%	11.9%	11.9%	10.8%	9.3%	7.5%	8.7%	8.2%	7.4%	4.8%
UWDuke_MCF7	19.6%	11.9%	11.6%	10.3%	9.0%	7.2%	8.5%	7.9%	7.7%	6.3%
Duke_MCF7Hypoxia	20.6%	12.1%	11.5%	10.2%	8.7%	7.2%	8.1%	7.5%	7.7%	6.2%
Duke_Medullo	20.3%	12.6%	12.1%	10.7%	9.0%	7.2%	8.1%	7.1%	6.9%	5.9%
Uw_NHDFneo	19.5%	12.8%	12.7%	11.4%	9.5%	7.6%	8.6%	7.4%	6.4%	4.1%
UWDuke_HSMMtube	18.3%	12.2%	12.1%	10.9%	9.4%	7.6%	8.6%	7.8%	7.4%	5.7%
Uw_HIPEpiC	18.3%	12.3%	12.3%	11.3%	9.5%	7.9%	8.9%	8.1%	7.2%	4.2%
Uw_NHA	18.0%	12.0%	12.3%	11.3%	9.8%	7.9%	9.0%	8.0%	7.1%	4.4%
Uw_GM06990	14.2%	9.7%	10.5%	10.0%	9.0%	7.7%	9.8%	9.8%	10.6%	8.5%
Duke_HTR8svn	17.1%	11.0%	11.3%	10.3%	9.1%	7.5%	8.7%	8.7%	8.9%	7.4%
Uw_Caco2	17.0%	10.3%	10.2%	10.0%	9.0%	7.9%	9.5%	9.3%	9.6%	7.2%
Duke_pHTE	19.2%	11.8%	11.5%	10.6%	9.1%	7.4%	8.5%	7.9%	7.9%	6.1%
UWDuke_GM12878	15.9%	10.4%	10.3%	9.7%	8.4%	7.0%	8.9%	9.1%	10.1%	10.1%
UWDuke_HSMM	17.8%	12.2%	12.2%	11.3%	9.3%	7.7%	8.6%	7.8%	7.3%	5.8%
Uw_HRGEC	16.5%	11.5%	11.8%	10.8%	9.5%	7.7%	9.2%	8.9%	8.5%	5.6%
Duke_GM12892	18.2%	11.3%	10.8%	10.1%	8.6%	7.3%	8.6%	8.6%	9.1%	7.6%
Th2wb54553204_UW	15.0%	10.3%	10.5%	9.9%	8.9%	7.7%	9.2%	9.8%	10.5%	8.4%
UWDuke_Th1	20.4%	12.0%	11.5%	10.0%	8.5%	6.8%	8.0%	7.6%	8.0%	7.2%
Uw_HMVECdLyAd	14.5%	10.7%	11.2%	10.6%	9.3%	7.9%	9.5%	9.5%	9.6%	7.2%
Duke_Melano	22.0%	13.3%	12.7%	10.9%	9.0%	6.9%	7.6%	6.7%	6.2%	4.8%
Uw_MonocytesCD14RO01746	14.9%	10.1%	10.6%	9.7%	8.6%	7.2%	9.2%	10.1%	11.0%	8.6%
Uw_WERIRb1	19.7%	13.1%	12.7%	11.1%	9.4%	7.4%	8.3%	7.2%	6.6%	4.6%
Uw_HCM	18.0%	12.0%	12.2%	11.1%	9.7%	7.8%	8.9%	8.2%	7.5%	4.6%
Duke_Huh7.5	19.1%	11.8%	11.2%	10.3%	8.7%	7.3%	8.4%	7.8%	8.2%	7.3%
Duke_CLL	15.5%	10.1%	9.5%	9.3%	8.2%	7.0%	9.0%	9.8%	11.1%	10.6%
Uw_HMVECdLyNeo	14.1%	10.6%	11.2%	10.6%	9.5%	8.0%	9.7%	9.6%	9.7%	6.9%
Duke_GM19240	19.8%	11.7%	11.2%	10.0%	8.5%	6.9%	8.1%	7.9%	8.4%	7.5%
Duke_GM19238	17.4%	10.9%	10.4%	9.7%	8.5%	7.0%	8.8%	8.9%	9.5%	8.8%
Uw_HAc	18.7%	12.6%	12.6%	11.4%	9.6%	7.7%	8.7%	7.7%	6.7%	4.3%
Tregwb83319432_UW	16.4%	10.6%	10.4%	9.8%	8.5%	7.2%	9.2%	9.4%	10.0%	8.6%
Uw_HL60	16.2%	11.1%	11.1%	10.2%	8.9%	7.4%	9.0%	9.3%	9.8%	7.1%
Uw_HCPEpiC	19.0%	12.5%	12.4%	11.1%	9.6%	7.6%	8.7%	7.9%	7.0%	4.4%
Uw_NT2D1	20.2%	12.7%	12.2%	10.7%	9.0%	7.3%	8.3%	7.6%	7.0%	4.8%
Uw_HPF	17.8%	11.7%	11.8%	11.0%	9.5%	7.8%	9.1%	8.3%	7.6%	5.2%
Duke_HPDE6E6E7	16.8%	10.9%	10.9%	10.3%	9.0%	7.4%	9.1%	8.8%	9.2%	7.6%
Duke_FibroP	20.7%	12.7%	12.1%	11.0%	9.0%	7.3%	8.2%	7.1%	6.8%	5.1%
Uw_AoAF	18.9%	12.2%	12.2%	11.1%	9.6%	7.8%	8.7%	7.9%	7.0%	4.5%
Uw_SKNSHRA	14.1%	9.7%	10.5%	10.2%	9.3%	8.1%	10.0%	9.8%	10.1%	8.2%
Uw_HPAEC	15.3%	10.7%	11.1%	10.4%	9.2%	7.7%	9.4%	9.4%	9.7%	7.1%
Duke_HeLaS3IFNa4h	17.8%	10.9%	10.6%	10.0%	8.7%	7.4%	8.8%	8.7%	9.0%	8.0%
Uw_HCF	18.0%	11.9%	11.9%	10.9%	9.5%	7.8%	8.9%	8.3%	7.6%	5.1%
Uw_RPTEC	19.0%	12.6%	12.4%	11.1%	9.5%	7.5%	8.6%	7.8%	7.0%	4.4%
Uw_HRPEpiC	17.5%	12.5%	12.4%	11.4%	10.0%	8.0%	9.0%	7.8%	7.0%	4.5%
Duke_Chorion	18.9%	11.7%	11.3%	10.4%	8.9%	7.4%	8.7%	7.9%	8.0%	6.7%
Uw_CD34Mobilized	15.2%	10.6%	10.9%	10.2%	9.1%	7.6%	9.2%	9.5%	10.1%	7.6%
Duke_Gliobla	18.1%	11.5%	11.3%	10.4%	8.9%	7.3%	8.6%	8.4%	8.4%	7.0%
UWDuke_HMEC	19.0%	11.7%	11.5%	10.5%	8.9%	7.4%	8.5%	7.9%	7.8%	6.7%

Uw_AG09309	19.3%	12.8%	12.7%	11.4%	9.6%	7.6%	8.4%	7.5%	6.6%	4.1%
Duke_Myometr	17.7%	11.5%	11.4%	10.6%	9.1%	7.4%	8.9%	8.3%	8.3%	6.9%
Uw_HGF	19.3%	12.4%	12.1%	10.9%	9.2%	7.6%	8.5%	7.9%	7.1%	4.8%
Uw_AG04449	20.5%	12.8%	12.6%	11.3%	9.3%	7.3%	8.2%	7.4%	6.5%	4.1%
Uw_HVMF	17.1%	11.8%	12.2%	11.3%	9.9%	8.1%	9.3%	8.4%	7.4%	4.5%
Duke_Th0	20.3%	11.8%	11.3%	10.1%	8.4%	6.9%	8.3%	7.7%	8.1%	7.2%
Duke_PanIsletD	18.8%	11.8%	11.7%	10.7%	9.1%	7.4%	8.6%	7.9%	7.7%	6.2%
Uw_HMVECdAd	14.2%	10.5%	10.8%	10.4%	9.2%	7.9%	9.5%	9.5%	10.1%	7.9%
Duke_UrotheliaUT189	18.9%	11.3%	11.2%	10.3%	8.7%	7.2%	8.6%	8.1%	8.4%	7.2%
Uw_NHDFAd	20.1%	12.9%	12.5%	11.5%	9.4%	7.5%	8.4%	7.4%	6.4%	3.9%
UWDuke_HepG2	17.9%	11.2%	10.7%	9.7%	8.6%	7.4%	8.8%	8.5%	8.9%	8.4%
UWDuke_NHEK	17.2%	11.1%	11.0%	10.2%	9.0%	7.5%	8.9%	8.7%	8.7%	7.7%
Duke_ProgFib	19.8%	12.2%	11.7%	10.8%	8.9%	7.2%	8.2%	7.6%	7.5%	6.1%
Uw_HAEpiC	18.2%	12.1%	12.2%	11.2%	9.6%	7.8%	9.2%	8.1%	7.1%	4.4%
Duke_IshikawaTamoxifen	17.3%	11.3%	11.1%	10.1%	8.7%	7.1%	8.7%	8.6%	9.0%	8.1%
UWDuke_HeLaS3	16.6%	10.6%	10.7%	9.8%	8.8%	7.5%	8.8%	8.9%	9.3%	9.1%
Uw_HMVECdBlNeo	15.2%	11.1%	11.4%	10.8%	9.5%	8.0%	9.4%	9.2%	9.1%	6.3%
Duke_LNCaPAndrogen	18.0%	11.2%	10.9%	9.8%	8.6%	7.2%	8.5%	8.5%	9.0%	8.3%
Uw_HFFMyc	18.4%	12.3%	12.4%	11.1%	9.3%	7.6%	8.8%	8.0%	7.4%	4.6%
Uw_HCFaa	19.5%	12.7%	12.7%	11.4%	9.6%	7.5%	8.6%	7.7%	6.6%	3.7%
Uw_HAh	19.7%	13.2%	12.8%	11.4%	9.6%	7.6%	8.5%	7.2%	6.3%	3.8%
Uw_WI38TamoxifenTamoxifen	18.5%	12.2%	12.3%	11.1%	9.5%	7.7%	8.9%	8.2%	7.3%	4.3%
Th1wb54553204_UW	16.3%	10.7%	10.6%	9.9%	8.7%	7.3%	9.1%	9.4%	9.9%	8.0%
Uw_H7hESC	19.3%	12.4%	12.0%	10.9%	9.3%	7.7%	8.7%	7.9%	7.3%	4.6%
Uw_HAsp	21.5%	13.8%	13.1%	11.5%	9.3%	7.2%	7.9%	6.7%	5.6%	3.3%
Uw_HNPCEpiC	18.3%	12.2%	12.3%	11.4%	9.6%	7.9%	9.0%	8.0%	7.1%	4.2%
Uw_HMF	17.7%	11.8%	12.1%	11.1%	9.6%	7.9%	9.0%	8.3%	7.5%	5.0%
Uw_CMK	15.4%	10.7%	11.0%	10.2%	8.9%	7.5%	9.1%	9.5%	10.0%	7.5%
Uw_HCT116	16.9%	11.1%	11.2%	10.4%	9.1%	7.7%	9.1%	8.8%	8.6%	7.1%
Th1_UW	12.7%	8.9%	9.6%	9.5%	8.9%	7.7%	10.0%	10.6%	12.1%	10.0%
Uw_SKNMC	19.0%	13.0%	12.7%	11.4%	9.4%	7.2%	8.2%	7.6%	7.0%	4.7%
Duke_IshikawaEstradiol	17.7%	11.3%	11.2%	10.2%	8.7%	7.2%	8.7%	8.3%	8.8%	7.9%
Duke_GM18507	15.0%	10.0%	10.2%	9.4%	8.3%	7.2%	9.0%	9.6%	10.9%	10.3%
UWDuke_A549	16.7%	10.7%	10.5%	9.7%	8.5%	7.2%	8.7%	9.0%	9.6%	9.4%
Duke_Stellate	17.8%	11.4%	11.4%	10.5%	8.9%	7.3%	8.6%	8.4%	8.6%	7.1%
Uw_HPdLF	20.0%	12.8%	12.7%	11.4%	9.5%	7.5%	8.3%	7.4%	6.4%	4.0%
Uw_GM12864	17.0%	10.6%	10.8%	9.9%	8.7%	7.2%	9.2%	9.3%	9.8%	7.6%
Uw_PANC1	20.2%	12.5%	12.0%	10.7%	9.0%	7.3%	8.5%	7.9%	7.2%	4.7%
Uw_GM12865	16.9%	11.2%	11.1%	10.1%	8.9%	7.5%	9.1%	9.1%	9.2%	7.1%
Uw_HMVECLLy	15.0%	11.0%	11.5%	10.6%	9.3%	8.0%	9.3%	9.1%	9.3%	7.0%
Duke_Hepatocytes	18.3%	11.6%	11.3%	10.1%	8.8%	7.2%	8.4%	8.1%	8.4%	7.8%
Uw_HPAF	17.8%	11.8%	12.0%	11.0%	9.6%	7.8%	8.9%	8.4%	7.7%	5.0%
Th2_UW	13.0%	9.0%	9.4%	9.3%	9.0%	7.8%	10.0%	10.6%	11.9%	10.0%
Th1wb33676984_UW	15.8%	10.7%	10.6%	9.9%	9.0%	7.5%	9.3%	9.5%	10.0%	7.6%
Uw_HConF	18.9%	12.1%	12.0%	11.0%	9.4%	7.7%	8.8%	7.9%	7.3%	5.0%
UWDuke_HUVEC	13.9%	10.0%	10.4%	10.0%	8.8%	7.7%	9.4%	9.6%	10.4%	9.7%
Duke_RWPE1	16.1%	10.8%	11.2%	10.3%	9.2%	7.7%	9.3%	8.8%	9.0%	7.6%
Duke_Osteobl	20.6%	12.7%	12.3%	10.8%	9.1%	7.2%	8.2%	7.2%	6.7%	5.1%
UWDuke_K562	15.9%	10.5%	10.7%	10.1%	9.0%	7.7%	9.1%	9.0%	9.5%	8.5%
Uw_NHLF	18.2%	12.2%	12.5%	11.3%	9.7%	7.9%	9.0%	8.0%	7.0%	4.2%
Duke_Huh7	18.1%	11.1%	11.0%	9.8%	8.7%	7.3%	8.6%	8.4%	8.9%	8.2%
Uw_HMVECdBlAd	13.7%	10.4%	11.4%	10.6%	9.6%	8.1%	9.6%	9.8%	9.9%	6.9%
Duke_Urothelia	16.1%	10.4%	10.7%	10.1%	8.6%	7.5%	9.0%	9.0%	9.9%	8.7%
Uw_HMVECLBl	15.0%	11.0%	11.6%	10.7%	9.5%	7.9%	9.5%	9.2%	9.3%	6.2%
Uw_Th2	14.8%	10.2%	10.3%	10.0%	8.8%	7.8%	9.5%	9.7%	10.3%	8.6%
Duke_iPS	19.7%	11.9%	11.4%	10.3%	8.7%	7.3%	8.4%	8.0%	8.0%	6.3%
Duke_Fibrobl	20.9%	12.9%	12.4%	10.9%	9.1%	7.2%	8.1%	7.0%	6.6%	4.8%
Uw_HRE	17.3%	12.0%	12.2%	11.2%	9.6%	8.0%	9.0%	8.2%	7.6%	4.8%
Uw_CD20	14.6%	9.8%	9.9%	9.2%	8.3%	7.1%	9.0%	9.8%	11.1%	11.1%
Duke_HSMMemb	16.3%	10.9%	11.0%	10.2%	9.1%	7.7%	9.3%	8.9%	9.2%	7.4%
Uw_HRCEpiC	17.4%	12.0%	12.2%	11.2%	9.7%	7.8%	9.0%	8.2%	7.6%	4.9%
Uw_Jurkat	16.7%	11.9%	11.6%	10.5%	8.9%	7.4%	8.5%	8.9%	9.0%	6.6%
Th2wb33676984_UW	15.7%	10.7%	10.6%	10.0%	8.7%	7.6%	9.3%	9.5%	10.0%	8.1%
Duke_GM19239	16.7%	10.5%	10.4%	9.6%	8.4%	7.2%	8.8%	9.2%	9.9%	9.2%
Uw_HEEpiC	19.5%	12.3%	12.1%	11.0%	9.5%	7.5%	8.7%	7.9%	7.1%	4.5%
Duke_AoSMC	18.3%	11.6%	11.7%	10.9%	9.2%	7.5%	8.7%	8.2%	7.8%	6.1%
masterlist	12.5%	9.0%	9.7%	9.8%	9.1%	8.2%	10.2%	10.3%	11.0%	10.2%
